# Non‐Invasively Quantitative Raman Imaging for Calculation of Lymphatic Transport Kinetics of Micelles in Mice

**DOI:** 10.1002/advs.77634

**Published:** 2026-09-08

**Authors:** Yunlu Li, Hongzheng Lin, Sheng Yu, Yunhui Liao, Teng Huang, Zeyu Xiao, Wei Lu

**Affiliations:** ^1^ School of Pharmaceutical Sciences Minhang Hospital Key Laboratory of Smart Drug Delivery Ministry of Education State Key Laboratory of Molecular Engineering of Polymers Fudan University Shanghai China; ^2^ Department of Pharmacology and Chemical Biology Institute of Molecular Medicine Shanghai Jiao Tong University School of Medicine Shanghai China; ^3^ Hangzhou Institute of Medicine (HIM) Chinese Academy of Sciences Hangzhou Zhejiang China; ^4^ Quzhou Fudan Institute Quzhou Zhejiang China

**Keywords:** lymphatic transport, non‐invasive Raman imaging, pharmacokinetic model, quantitative analysis, vaccine

## Abstract

Raman spectroscopy based on surface enhanced Raman scattering (SERS) has received great interest in biological and medical applications owing to its high specificity and sensitivity. However, a reliable and quantitative SERS analysis in vivo is a great challenge since SERS is affected by the unevenly distributed field enhancement in hot spots of nanoparticle substrate and unpredictable nanoparticle aggregation in the complex biological environment. Here, we present non‐invasively quantitative Raman imaging through stacking‐induced intermolecular charge transfer‐enhanced Raman scattering (SICTERS) without relying on substrate. Based on the Raman imaging with SICTERS micelles, a quantitative method is established to accurately calculate the concentration of micelles in draining lymph nodes (DLNs) following subcutaneous administration. A physiologically based pharmacokinetic model is generated to fit the concentration‐time curve of the SICTERS micelles in DLNs (R^2^ > 0.99), which calculates the pharmacokinetic parameters of the lymphatic transport of micelles with different sizes and surface charges. The optimized SICTERS micelles loading ovalbumin (OVA) as model antigen exhibits significant anti‐tumor effect in mice bearing B16‐OVA melanoma following vaccination. This study demonstrates a quantitative in vivo Raman imaging platform based on SICTERS for image‐based pharmacokinetic modeling of micellar transport in lymphatics.

## Graphical abstract

Non‐invasively quantitative Raman imaging with stacking‐induced charge transfer‐enhanced Raman scattering (SICTERS) enables accurate measurement of concentrations and pharmacokinetic modeling of lymphatic transport of micelles in mice. This platform allows dynamic analysis of nanoparticle transport and underpins the design of effective lymph node‐targeted vaccines with enhanced antitumor efficacy.

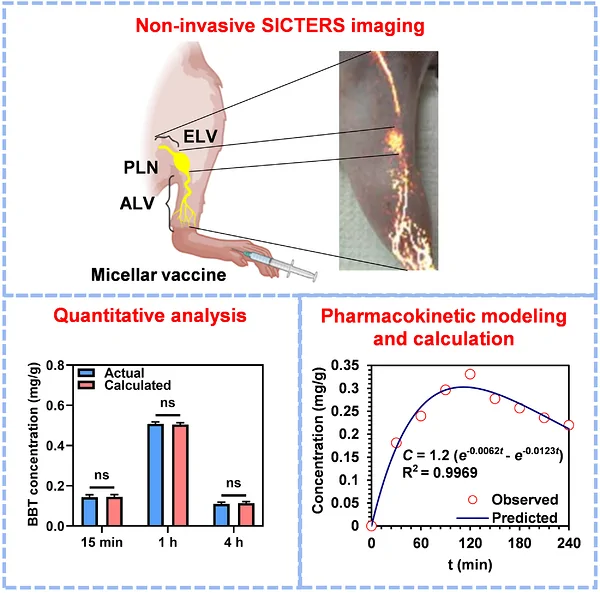

## 1 Introduction

Raman imaging has advantages of high specificity and low susceptibility to photobleaching, showing great potentials for biomedical application [1, 2, 3, 4, 5]. By using the surface enhanced Raman scattering (SERS), small Raman molecules are adsorbed onto inorganic Au or Ag substrate whose Raman scattering signal can be remarkably amplified [6, 7, 8]. However, the quantitative SERS remains a great challenge. Due to the inherently near‐field phenomenon of SERS, the overall Raman signals are determined by coherent collective electron oscillations, the so‐called localized surface plasmon resonances, which induce enhanced electromagnetic fields (hot spots) surrounding the metallic nanostructure. The field enhancement in the hot spots may vary by several orders of magnitude depending on the local structure like nanoparticle size [9], shape [9, 10], crystal face [11], surface roughness [12], dispersing state [13], and interparticle spacing [14, 15], even if the Raman molecules are uniformly distributed on the metal surface [16, 17]. Moreover, the uncontrollable aggregation of nanoparticles resulted in the random distribution of hot spots with subsequent signal heterogeneity [18, 19, 20, 21, 22]. Through the incorporation of Raman internal standard, a reliable and direct quantitative SERS analysis has been achieved in vitro [23]. Nevertheless, the much more complex biological environment in vivo causes nanoparticles to produce unpredictable nano distance and surface deformation, resulting in fluctuations in Raman signal [24, 25, 26, 27]. To date, in vivo quantitative SERS imaging has not been accomplished.

Recently, we have reported the stacking‐induced charge transfer‐enhanced Raman scattering (SICTERS), which can enhance the Raman scattering signal of small molecules by self‐stacking without relying on an external metal substrate [28, 29]. Compared with the traditional SERS gold nanoprobe, the Raman scattering cross‐section per SICTERS nanoparticle is 1350 times higher [28]. A side‐by‐side comparison of in vivo imaging between SICTERS nanoparticles with 4‐nitrobenzenethiol (4‐NBT)‐labeled gap‐enhanced Raman tags (Au GERTs) has been performed [28]. The intradermal injection of Au GERTs at a dose of 80 µg of Au does not yield transdermal Raman signals in axillary lymph nodes (ALNs), while the injection of the SICTERS nanoparticles at a dose of 20 µg SICTERS molecules offers noninvasive transdermal imaging of ALNs. Besides, unlike SERS dependent on the hot spots formed by local metal structure and the distribution patterns of Raman molecules onto metal surface, the SICTERS molecules stack in the core of the micellar structure through nano‐assembly. The particle size of SICTERS nanoparticles does not cause changes in the intermolecular distance of BBT stacks, or not interfere with the Raman signal [28], which provides a rationale for in vivo quantitative analysis of SICTERS.

Vaccines play a pivotal role in preventing and controlling human infectious diseases and cancer [30, 31, 32, 33]. The SICTERS nanoprobes offer non‐invasive and highly sensitive Raman imaging of lymphatic drainage in mice, which has not been achieved by SERS [28]. However, there is a lack of an approach to quantitative analysis of nanoparticle transport from lymphatic vessels to lymph nodes based on real‐time Raman imaging and their pharmacokinetic modeling and calculation.

**FIGURE 1:**
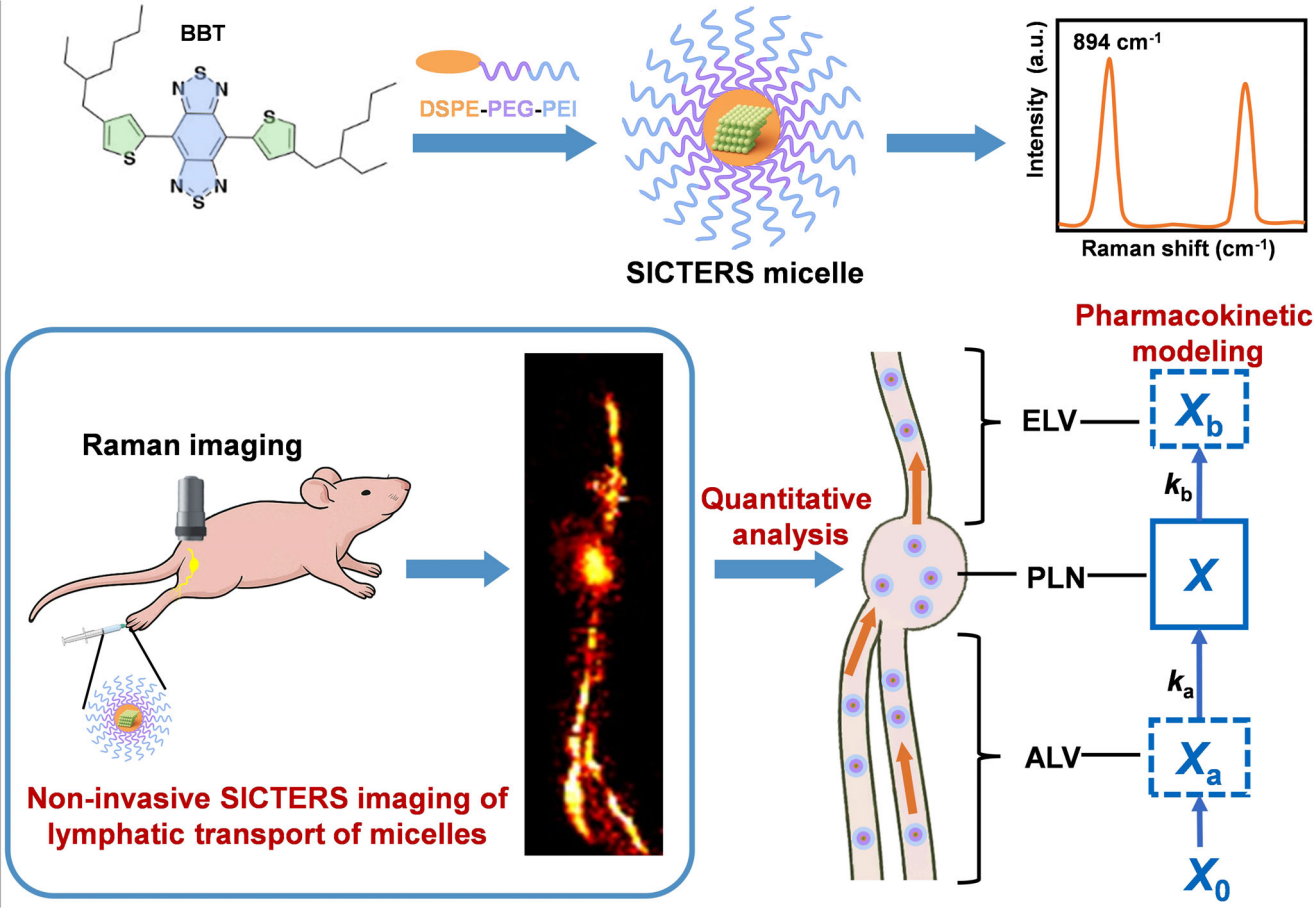
Illustration of SICTERS micelles for non‐invasive Raman imaging of lymphatic transport for quantitative analysis and PBPK modeling.

Here, we use 1,2‐distearoyl‐sn‐glycero‐3‐phosphoethanolamine‐polyethyleneimine (DSPE‐PEG‐PEI) to encapsulate SICTERS molecule 4,7‐bis(4‐(2‐ethylhexyl)thiophen‐2‐yl)benzobis [*c*] [1,2,5] thiadiazole (BBT) to prepare micelles for non‐invasively continuous Raman imaging of their lymphatic transport following subcutaneous injection into the footpad of mice. A standard curve is established by the quantitative method, that calculates the concentration of SICTERS micelles in the popliteal lymph node (PLN), afferent lymphatic vessel (ALV), or efferent lymphatic vessel (ELV) of mice based on the SICTERS images. The accuracy of the calculated concentration in PLN based on the Raman imaging is verified by the concentration directly measured through tissue extraction. The concentration‐time curve of SICTERS micelles in PLN is acquired to calculate *C*_max_ and area under the curve (AUC). Moreover, we generate a physiologically based pharmacokinetic (PBPK) model to calculate pharmacokinetics, including influx rate constant *k*_a_ and efflux rate constant *k*_b_ (Figure 1). Four types of micelles with different particle sizes and surface charges were compared to demonstrate differences in pharmacokinetics. Lastly, the optimized SICTERS micelles loading antigen ovalbumin (OVA) as micellar vaccines significantly inhibit the growth of B16‐OVA melanoma in mice.

## 2 Results

### 2.1 Quantitative Analysis of SICTERS Micelles in Lymphatics Through Non‐Invasive Raman Imaging

**FIGURE 2:**
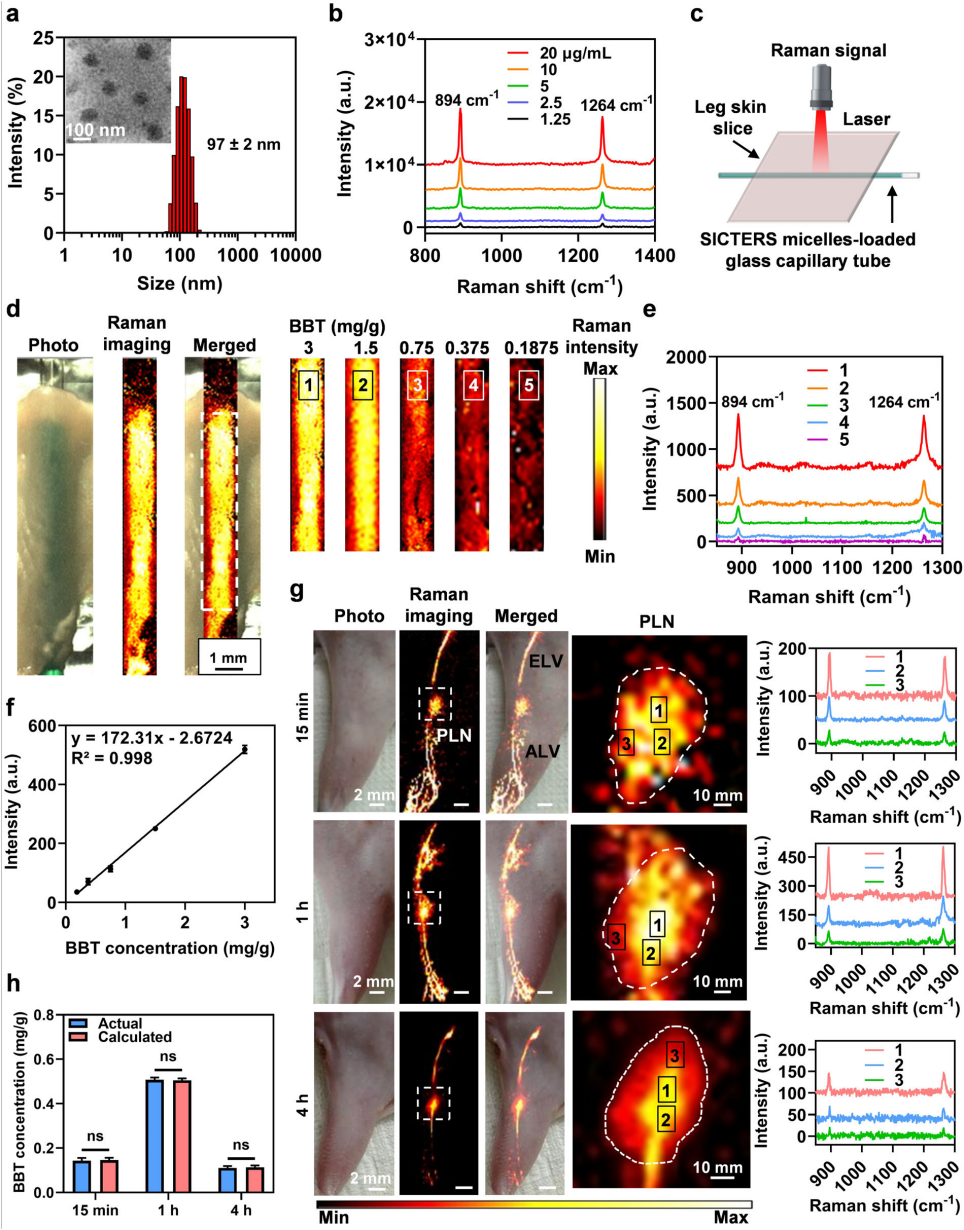
Quantitative analysis of SICTERS micelles in lymphatics through non‐invasive Raman imaging. (a) Size distribution of PEI‐100 micelles. Insert: transmission electron micrograph. (b) Raman spectra of PEI‐100 micelles with different concentrations of BBT. (c) Schematic diagram of the quantitative method. (d) Raman imaging of capillary glass tube (inner diameter = 1 mm) containing PEI‐100 micelles covered with the leg skin of mice on the top. Right, Raman imaging of PEI‐100 micelles at different concentrations. (e) Raman spectra in (d). (f) Standard curve fitted between Raman intensity (894 cm^−1^) in (e) and BBT concentration. (g) Non‐invasive Raman imaging (894 cm^−1^) of PLN, ALV and ELV of mice after subcutaneous injection PEI‐100 micelles (3 mg/mL, 50 µL) for different times. Right, Raman spectra. (h) The comparison between the BBT concentrations calculated from the average Raman intensity (894 cm^−1^) of all the pixels in the circled PLN area in (g) based on the standard curve (f) and those measured by fluorescence analysis of the extracts from the PLN homogenate (actual). (f) and (h), mean ± SD (*n* = 3). ns, no significant difference analyzed by two‐way ANOVA with Tukey's post‐hoc test.

Four types of SICTERS micelles were prepared. DSPE‐PEG‐PEI was used to encapsulate BBT for preparing PEI‐100 micelles and PEI‐170 micelles with average diameters of 97 and 168 nm, and average zeta potentials of 11.47 and 9.97 mV, respectively (Figures 2a and S1). The negatively charged COOH micelles were prepared using 1,2‐distearoyl‐sn‐glycero‐3‐phosphoethanolamine‐N‐[carboxy (polyethylene glycol)] (DSPE‐PEG‐COOH) to encapsulate BBT, characterized by an average zeta potential of ‐32 mV. The PEI‐100 micelles were further used to prepare micellar vaccines through electrostatic adsorption of antigen OVA and adjuvant oligodeoxynucleotides containing CpG motifs (CpG) (Figure S1). When the ratio of DSPE‐PEG‐PEI to total lipid was 0.3, the optimal micellar vaccines were obtained with OVA and CpG encapsulation efficiency of 52.15% and 51.53%, respectively, and an average zeta potential of −1.5 mV close to neutral (Figure S1). Both COOH micelles and micellar vaccines had an average diameter of 100 nm. The raw Raman spectrum of PEI‐100 micelle showed characteristic peaks at 894 cm^−^^1^ and 1264 cm^−^^1^ with a gradually increasing fluorescence background. After baseline correction, the Raman shifts of both peaks remained unchanged, the Raman peak intensity variation relative to the baseline at 894 cm^−^^1^ was merely 0.6% compared with the raw spectrum (Figure S2), indicating that the fluorescence background did not significantly influence the Raman shift or intensity of the characteristic peak at 894 cm^−^^1^. The Raman spectra of all SICTERS micelles showed characteristic peaks at 894 and 1264 cm^−1^ with similar signal‐to‐noise ratios (Figures 2b and S3). The Raman intensity (894 cm^−1^) of all the micelles showed a good linear relationship with BBT concentration.

The PLNs and their draining lymphatic vessels are located under the mouse leg skin and on the surface of superficial muscle tissue at a depth of approximately 0.3–0.5 mm [34, 35]. Based on such anatomic structure, a quantitatively analytical method was developed through which a capillary glass tube containing different concentrations of PEI‐100 micelles was covered with depilated mouse leg skin (Figure 2c). Raman imaging of the micelles in the glass was performed from the top of the skin excited at 785 nm (Figure 2d,e). At a concentration ranging from 0.1875 to 3 mg/g of BBT, the Raman intensity of the micelles showed a good linear relationship with their concentrations (R^2^ = 0.9980, Figure 2f). After subcutaneous injection of PEI‐100 micelles into the mouse footpad, the maximum penetration depth of SICTERS Raman imaging was 2.0 mm by covering exposed PLNs with various pieces of porcine skin slices, demonstrating the ability of non‐invasive Raman imaging of superficial lymphatic structures in mice (Figure S4). To calculate the concentrations in vivo, the mice were divided into 3 groups, non‐invasive Raman imaging was conducted at 15 min, 1 h, and 4 h post subcutaneous injection of PEI‐100 micelles into the mouse footpad, respectively. The images clearly delineated the location and morphology of PLNs and lymphatic vessels with a high signal‐to‐noise ratio (Figure 2g). The average value of Raman intensity (894 cm^−1^) of all the pixels in the circled PLN area in Figure 2g was substituted into the standard curve in Figure 2f to obtain the calculated BBT concentrations. To verify the accuracy of the calculated values, the PLNs of mice following the imaging at each time point post injection were collected. BBT were extracted from the PLN homogenate, whose actual concentrations were measured by fluorescence analysis. The results confirmed that the calculated BBT concentrations in PLN were consistent with the actual concentrations (Figure 2h). Besides, the minimum injection dose of SICTERS micelles for the non‐invasively quantitative analysis of PLN was 25 µg of BBT (Figure S5).

### 2.2 Continuously Quantitative Imaging and Non‐Compartmental Analysis of Lymphatic Transport of SICTERS Micelles

**FIGURE 3:**
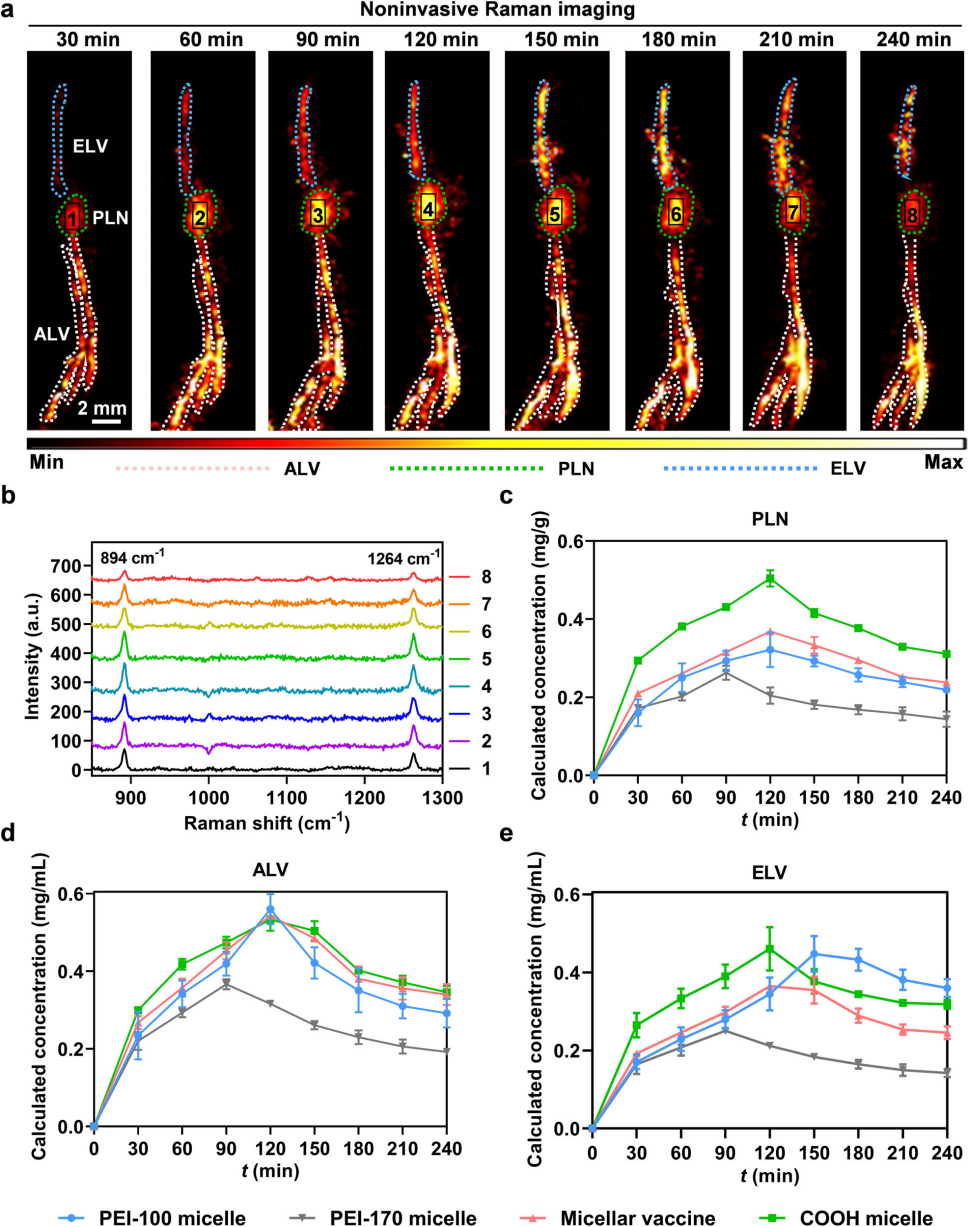
Continuously quantitative imaging and non‐compartmental analysis of lymphatic transport of SICTERS micelles. (a) After subcutaneous injection of PEI‐100 micelles (3 mg/mL, 50 µL) into the footpad of Mouse 1, non‐invasive Raman imaging (894 cm^−1^) was conducted at predetermined time points. (b) Raman spectra in (a). (c–e) The BBT concentration‐time curves of different micelles in PLN (c), ALV (d) or ELV (e). The BBT concentration was calculated by substituting the averages of Raman intensity (894 cm^−1^) of all pixels in the dotted line‐circled area at each time point in (a) into Figure 2f. Mean ± SD (*n* = 3).

We then performed continuous Raman imaging to track the lymphatic transport of different SICTERS micelles in mice (Figures 3a,b and S6–S16). The Raman intensity of pixels that exceeded the sensitivity (15 a.u.) in the location of lymphatic vessels or lymph nodes was circled to calculate the average intensity. These average values were used to calculate the concentrations of BBT based on the standard curve in Figure 2f to obtain the concentration‐time curve of PLN, ALV, and ELV using non‐compartment model, respectively (Figure 3c–e). Compared with PEI‐100 micelles, PEI‐170 micelles with larger particle size exhibited significantly lower *C*_max_ and reduced AUC values in PLN, ALV, and ELV, indicating less lymphatic transport for larger‐sized micelles (Figures 3c–e and S17). With similar particle sizes, the negatively charged COOH micelles showed significantly higher *C*_max_ and AUC in PLN and ALV than the positively charged PEI‐100 micelles, respectively, suggesting that a negatively surface charge facilitates lymphatic transport of micelles (Figures 3c,d and S18–S20). The micellar vaccines following loading the antigen and adjuvant did not significantly change *C*_max_ and AUC in PLN or ALV compared with PEI‐100 micelles (Figures 3c,d and S18–S20). During continuous Raman imaging, the skin surface temperature increased from 32.1°C to 33.9°C (Figure S21a), which complies with clinical safety standards for contact optical imaging systems (< 43°C) [36, 37]. After 240 min of the entire imaging study, the mouse skin, PLNs, ALVs, and ELVs were collected for hematoxylin and eosin (H&E) staining, respectively (Figure S21b). The epidermal and dermal morphology of skin tissue remained intact without obvious inflammatory infiltration, necrosis, or tissue damage. PLNs, ALVs, or ELVs did not show evident histologic damage or inflammatory response. Next, we evaluated the photostability of SICTERS micelle after repeated Raman imaging (Figure S22a). The Raman intensity at 894 cm^−^^1^ maintained 97.5%–99% (Figure S22b), suggesting that repeated Raman imaging did not substantially affect quantitative signal readout. UV–vis absorption (Figure S22c) and fluorescence spectra (Figure S22d) were nearly unchanged, indicating minimal photodegradation under the experimental conditions [38]. Moreover, SICTERS micelles did not show significant changes in particle sizes (Figure S22e), and the structure was relatively intact (Figure S22f).

### 2.3 Pharmacokinetics of SICTERS Micellar Lymphatic Transport via PBPK Model

**FIGURE 4:**
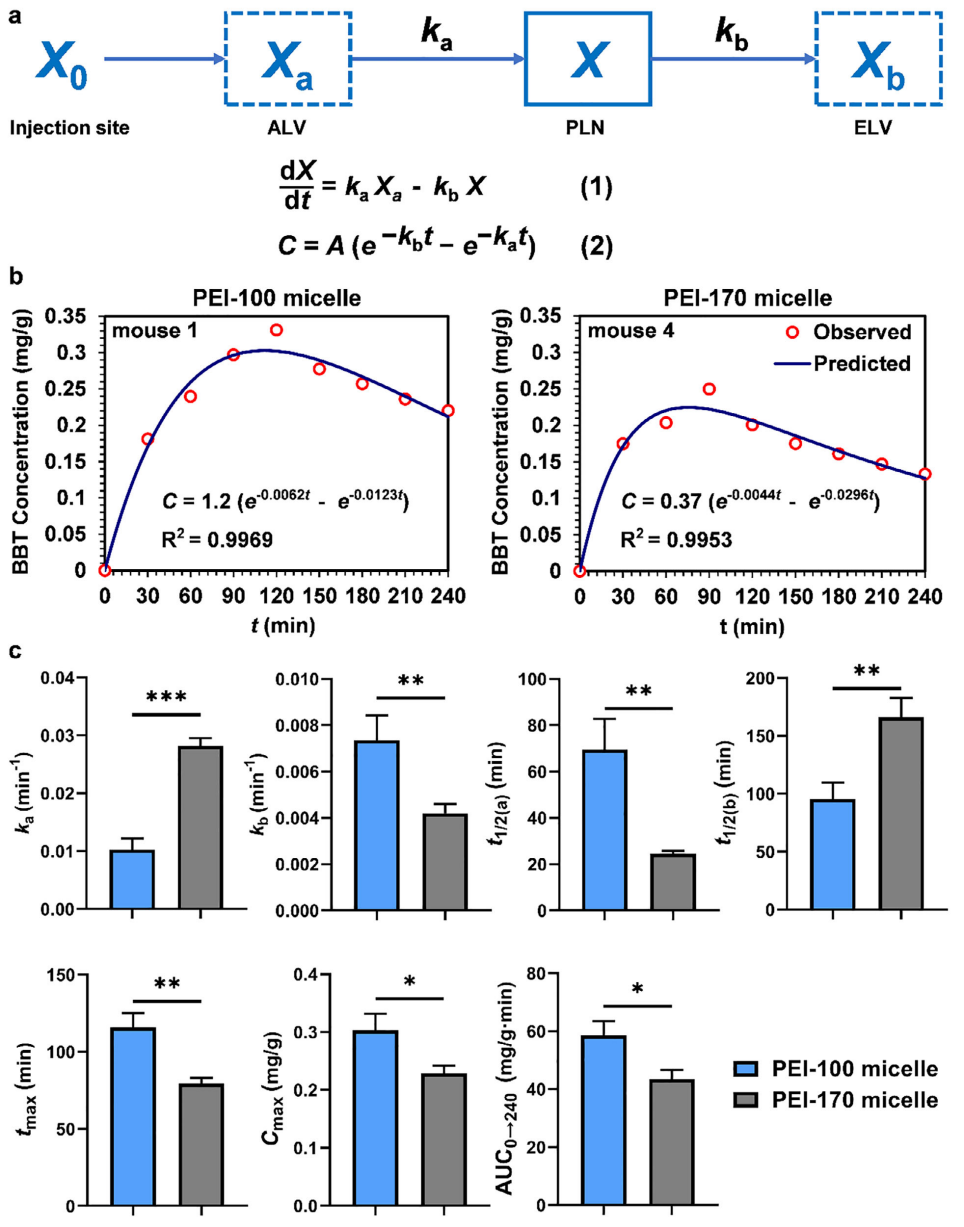
Pharmacokinetics of SICTERS micellar lymphatic transport via PBPK model. (a) Compartmental modeling of lymphatic transport of micelles following extra lymphatic administration. *X*_0_, dose amount of administration; *X*_a_, amount of micelles in ALVs; *X*, amount of micelles in PLN; *X*_b_, amount of micelles in ELV; *k*_a_, influx rate constant; *k*_b_, efflux rate constant; *C*, concentration of micelles in PLN. (b) The concentration‐time curves (predicted) of PEI‐100 micelles (mouse 1) and PEI‐170 micelles (mouse 4) in PLN by fitting the data of Figures 3a and S8 (observed) to Equation (2), respectively. (c) Pharmacokinetic parameters of PEI‐100 micelles and PEI‐170 micelles obtained from Figures 4b and S6–S10. *t*_1/2(a)_, influx half‐life; *t*_1/2(b)_, efflux half‐life; *t*_max_, time of the maximum concentration in PLN; *C*_max_, maximum concentration in PLN; AUC_0→240_, AUC from 0 to 240 min. Mean ± SD (*n* = 3). *p < 0.05, **p < 0.01, ***p < 0.001 analyzed by two‐tailed Student's *t*‐test.

Thanks to the valves in lymphatic vessels, the drainage of lymph fluid from ALV to PLN and then to ELV is unidirectional [39, 40]. Assuming the amount of SICTERS micelles in PLN (*X*) follows the first‐order rate (linear) process, a PBPK model is generated considering PLN as one single compartment (Figure 4a). The rate of change of *X* in PLN (d*X*/d*t*) conforms to the differential Equation (1):

(1) $$\frac{dX}{dt}=k_{a}X_{a}-k_{b}X$$

*k*_a_ and *k*_b_ indicate the influx rate constant from ALV and efflux rate constant to ELV, respectively (Figure 4a). Through formula derivation and calculation (see Supporting Information), the kinetic Equation (2) for lymphatic transport is obtained, where *C* is the concentration of SICTERS micelles in PLN and *A* is a constant.

(2) $$C=A\left(e^{-k_{b}t}-e^{-k_{a}t}\right)$$

The data of concentrations over time (Figure 3c) of all different micelles were well fitted with this kinetic model, evidenced by that the R^2^ values analyzed through least‐squares curve fitting algorithms of three mice were all above 0.99 (Figures 4b and S6–S16). The *t*_max_, *C*_max_, and AUC values of PLN were calculated without significant difference between using this PBPK model and by the non‐compartmental model above, further validating the feasibility of the PBPK model (Figure S23). The kinetic results revealed *k*_a_ > *k*_b_, supporting the observation that the lymphatic transport of SICTERS micelles in ALVs was faster than that in ELVs (Figures 4 and S24). Compared with PEI‐100 micelles, the lager‐sized PEI‐170 micelles showed significantly lower *C*_max_ and AUC in PLN attributed to quicker influx of smaller *t*_1/2(a)_ value and slower efflux of larger *t*_1/2(b)_ value (Figure 4c). These results demonstrated that the particle size had a pronounced impact on all the investigated pharmacokinetic parameters. However, with the same particle size of about 100 nm, the micellar surface charge did not significantly affect *k*_a_ and *k*_b_, but influenced *C*_max_ and AUC (Figure S24). Compared with the PEI‐100 micelles, the negatively charged COOH micelles exhibited significantly higher *C*_max_ and AUC, respectively. Whereas, the micellar vaccines of nearly neutral surface charge showed similar pharmacokinetic profiles with the PEI‐100 micelles, indicating that loading antigens and adjuvants did not significantly affect the lymphatic transport.

### 2.4 The Anti‐Tumor Immune Effect of Micellar Vaccines

**FIGURE 5:**
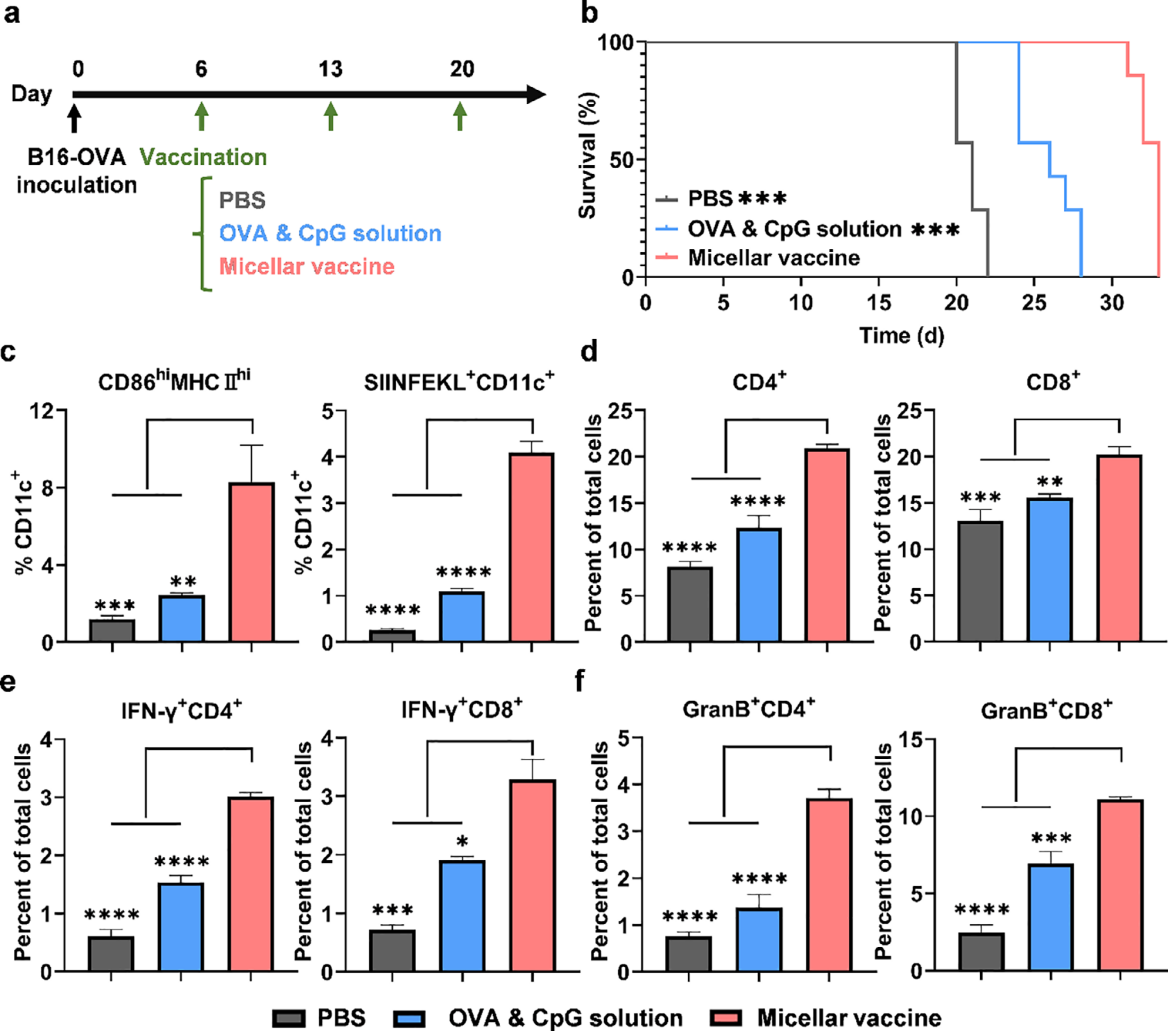
The anti‐tumor immune effect of micellar vaccines. (a) Regimen of vaccination. The details refer to the Supporting Information. (b) Kaplan‐Meier survival plots of (b) (*n* = 7). Statistical significance was calculated by log‐rank (Mantel‐Cox) test. (c–f) Flow cytometry analysis of different immune cells in PLNs of B16‐OVA‐bearing mice at Day 21 after tumor inoculation. Mean ± SD (*n* = 3). Statistical significance was calculated by one‐way analysis of variance (ANOVA) with Tukey's post‐hoc test. *p < 0.05, **p < 0.01, ***p < 0.001, ****p < 0.0001 compared with micellar vaccine group.

Micellar vaccines were immunized into mice bearing B16‐OVA melanoma according to the regimen shown in Figure 5a. The micellar vaccines significantly prolonged the median survival time of the tumor‐bearing mice (33 days) compared with the OVA & CpG solution group (26 days) (Figures 5b and S25). The immune cells in PLNs, spleen, or tumor of mice following different treatments were subjected to flow cytometric analysis (Figure S26). Compared with the OVA & CpG solution, the micellar vaccines significantly induced the activation and antigen presentation of dendritic cells (DCs) in PLNs (Figures 5c and S27). Moreover, the micellar vaccines increased the proportion of CD4^+^ T cells and CD8^+^ T cells in PLNs and spleen, promoting the secretion of interferon‐γ (IFN‐γ) and granzyme B (GranB), respectively (Figures 5d–f, S27 and S28). The micellar vaccines also significantly promoted the maturation and activation of DCs and the infiltration of CD8^+^ T cells in tumors, respectively (Figure S29). In the micellar vaccine group, the mouse body weights (Figure S30), organ coefficients (Figure S31), hematologic analysis indicators, and liver and kidney function indicators (Table S2) were all within normal ranges. The results of histopathological analysis by H&E staining did not show significant pathologic changes in major organs (Figure S32), preliminary confirming biocompatibility and biosafety of the micellar vaccines.

## 3 Discussion

The penetration depth of SICTERS imaging (2.0 mm) is superior to that of many reported SERS‐based Raman imaging (Table S3). SICTERS Raman imaging exhibits superior performance in quantitative analysis in vivo that SERS may not achieve [28]. SERS relies on the strong localized surface plasmon resonance generated on the surface of metal nanoparticles such as gold and silver. Moreover, the SERS effect usually depends on the random aggregation state of nanoparticles [16, 17]. When Raman reporter molecules are close to the hotspot regions, their Raman signals are dramatically amplified [41, 42]. However, due to the assembly of nanoparticles and the influence of the biological environment, the hotspots are distributed unevenly. In practical applications, biological fluids, proteins, and ions can influence the distribution and number of the hotspots, leading to poor signal reproducibility and unstable quantitative correlations [18, 19, 20, 21, 22, 26]. In contrast, SICTERS performs through electron charge transfer processes induced by the intermolecular stacking of Raman reporter molecules themselves, which does not require metal nanostructures. In the aggregated state through nano‐assembly, the molecules stack in the core of the nanoparticles or micelles, leading to more uniform and stable signal gain. Moreover, the size, surface charge, and surface adsorption of the micelles do not affect the aggregation of BBT molecules within the micellar core, thus resulting in relatively consistent Raman signals and making them comparable (Figure S3). The structure of molecular assembly provides a theoretical basis for the consistency and reproducibility of SICTERS imaging and precise quantitative analysis in vivo.

Fluorescence imaging has been widely adopted for in vivo biodistribution and lipid nanoparticle (LNP) tracking due to its high sensitivity, rapid imaging capability, and relatively deep tissue penetration [43, 44, 45]. The near‐infrared window II (NIR‐II) fluorescent lymphatic mapping was first pioneered using carbon nanotube‐derived probes [46]. Highly bright NIR‐II fluorophores were further developed for high‐resolution visualization of lymphatic vessels and sentinel lymph nodes [47]. However, for quantitative analysis in vivo, fluorescence intensity may not accurately reflect the intact LNPs, as fluorescence signal may be derived from dissociated or free fluorophores rather than nanoparticle‐associated labels [48, 49]. In a complex biological environment, the dissociation, leakage, or metabolic cleavage of fluorophores from nanocarriers leads to signals that misrepresent the actual LNP distribution [50]. Second, the fluorophores are susceptible to quenching and photobleaching under prolonged excitation, severely compromising long‐term continuous monitoring [51, 52]. Yet, to the best of our knowledge, fluorescence imaging has not been reported for quantitative pharmacokinetic modeling of lymphatic transport kinetics of LNPs.

Comparatively, the SICTERS Raman signal depends on the intermolecular stacking of BBT molecules within the micellar core. Once the micellar structure is disrupted, the ordered molecular stacking collapses and the Raman signal disappears [28]. Thus, the SICTERS signal reports the concentration of intact micelles. Second, Raman scattering is inherently resistant to photobleaching and quenching, enabling stable long‐term acquisition [1, 53]. However, compared with fluorescence imaging, Raman imaging is constrained by limited penetration depth, which is advantageous primarily in superficial layers and transparent tissues [41]. Moreover, because Raman imaging requires point‐by‐point acquisition of Raman signals, its imaging speed is much slower than that of fluorescence imaging [54, 55]. In recent years, the development of high‐penetration Raman microscope [56, 57] and high‐speed Raman instrument [58, 59] is expected to further broaden the applications of SICTERS in quantitative in vivo Raman imaging, extending beyond quantification of lymphatic transport. Furthermore, fluorescence imaging enables high‐sensitivity whole‐body visualization of LNP biodistribution, while Raman‐based SICTERS provides quantitative pharmacokinetic modeling of lymphatic transport. The two imaging modalities are highly complementary for integrated in vivo analysis of nanoparticle dynamics.

Inter‐individual variability in Raman imaging mainly arises from differences in skin thickness, tissue hydration, lipid/protein composition, lymphatic anatomy, thereby affecting signal intensity and spatial distribution [1, 40]. Our method used a skin‐covered quantification approach that mimicked the in vivo optical barrier to reduce errors caused by tissue attenuation mismatch [60]. Although our method improved the reliability of quantitative in vivo comparisons, it cannot fully eliminate biological heterogeneity. Future studies should employ larger study cohorts alongside mixed‐effects statistical models to separate biological variability from measurement variability [61]. Besides, Raman imaging combined with ultrasound imaging could provide anatomical depth correction and improve the accuracy of concentration estimation in heterogeneous tissues [62].

The efficacy of vaccines hinges upon the targeted delivery of antigens to lymph nodes to elicit sufficiently protective and therapeutic immune response [63, 64, 65, 66, 67]. Real‐time tracking of lymphatic transport of vaccines and analysis of their transport kinetics are imperative to evaluate and optimize the lymph node‐targeted delivery of vaccines, especially various nanovaccines [68]. A three‐compartment pharmacokinetic model has been generated to describe the transport efficiency of nanoparticles from tissues into lymphatic vessels [69]. However, quantitative analysis of nanoparticle transport from lymphatic vessels to lymph nodes based on real‐time imaging and their pharmacokinetic modeling and calculation has not yet been reported.

Here, we employ the first‐order kinetics of PBPK modeling to fit the concentration‐time curves of different micelles in DLNs with satisfactory fitting outcomes (Figure 4). The micelles with smaller particle size and higher negative surface charge exhibit greater accumulation (AUC), which is consistent with previous reports [70, 71, 72]. The micelles with smaller diameters and higher negative surface charges are more likely to enter the lymphatic circulation from the interstitial space and are more readily transported along with the lymphatic fluid [73, 74]. Beyond the extent of transport, the PBPK model partitions the lymphatic system into well‐defined structural compartments (e.g., ALV, PLN, and ELV) and analyzes the dynamic concentrations of micelles in these regions, respectively. As a result, the key parameters, including *C*_max_, *t*_max_, *t*_1/2(a)_, *t*_1/2(b)_, *k*_a,_ and *k*_b_ have been obtained to provide guidance for the precise design of the lymph node‐targeted delivery of nanovaccines (Figure 4). Our results demonstrate that the micellar vaccine with optimized lymph node‐targeting ability exhibits significant antitumor efficacy in a murine tumor model (Figure 5). The efficient transport to lymph nodes promotes the intranodal enrichment of antigens and adjuvants, further enhancing the DC‐mediated antigen presentation and the T cell‐mediated cytotoxic immune activity [75].

This study demonstrates a quantitative in vivo Raman imaging platform based on SICTERS for image‐based pharmacokinetic modeling of micellar transport in lymphatics in animals. Nevertheless, there remain substantial gaps before its clinical translation and practical application. The thickness of human skin significantly varies across anatomical sites. Human skin, including epidermis and dermis exhibits variable thickness, mainly ranging from 1 to 2.2 mm [76, 77]. High‐frequency ultrasound and optical coherence tomography studies have demonstrated that the skin thickness in the inguinal region of adults is 1.1–2.2 mm, while in the axillary region it is 1.0–1.8 mm [78, 79]. The cutaneous lymphatic plexus originates in the dermal papillary layer at a depth of only 0.3–0.8 mm. Whereas collecting lymphatic vessels are typically located in the superficial subcutaneous fat at depths of approximately 1–2 mm [80]. Inguinal superficial lymph nodes are located within subcutaneous tissue below the inguinal ligament. Ultrasound measurements indicate that some superficial lymph nodes are located at depths < 2 mm in certain individuals [81].

In humans, vaccines are normally administered into the deltoid muscle, and the primary lymphatic drainage is directed to the axillary lymph node [39]. These axillary lymph nodes are typically located several millimeters to centimeters beneath the skin surface depending on body habitus [82]. Axillary level I lymph nodes (low axillary lymph nodes) are within a range of 1–2 mm below the skin surface, particularly sentinel lymph nodes located near the lateral border of the pectoralis major muscle [83]. Accordingly, a penetration depth of approximately 2 mm achieved by SICTERS imaging may be used for imaging dermal lymphatic plexuses and partial superficial axillary lymph nodes. However, this technique under current imaging condition may be insufficient to visualize human lymph nodes located at depths more than 2 mm.

Clinically deployed Raman spectroscopy systems are mainly fiber‐optic‐probe‐based configurations designed for in vivo or intraoperative point measurements [84]. Representative Raman spectroscopy systems include fiber‐optic endoscopic systems that have demonstrated feasibility for real‐time diagnosis of gastrointestinal and epithelial cancers [85]. Handheld or intraoperative Raman probes have been used for neurosurgical margin assessment and dermatological imaging [86]. In our study, the Raman imaging system is a preclinical Raman confocal microscopy (Renishaw inVia system). The setup includes a 785 nm diode laser excitation source, a Raman microscope equipped with objectives, and a thermoelectrically cooled charged coupled device (CCD) for spectral acquisition. This configuration allows spatially resolved Raman imaging with step sizes down to the micrometer scale, enabling mapping of nanoparticle distribution in murine lymphatic tissues. While this instrument shares fundamental optical principles with clinically used fiber‐optic Raman probes, this preclinical imaging system has not yet been optimized for clinical application. Future clinical translation would require integration into sterilizable handheld or endoscopic probes, enhanced photon collection efficiency via optimized fiber bundles and transmission optics, and validation in large‐scale human cohorts [87].

Polymeric micelles such as PEI‐based or amphiphilic self‐assembled systems have been extensively investigated with favorable translational potential when administered at low doses with biodegradable or renally/hepatically excretable components [88]. However, clinical translation of micellar Raman probes requires comprehensive evaluation of reproducibility, batch‐to‐batch consistency, pharmacokinetics, immunogenicity, long‐term toxicity, and clearance pathways (hepatic and/or renal). Moreover, Food and Drug Administration (FDA) guidance for nanomedicine emphasizes systematic Good Laboratory Practice (GLP) toxicology studies and scalable Good Manufacturing Practice (GMP) manufacturing processes prior to human application [89, 90].

## 4 Conclusion

In summary, we developed a non‐invasive Raman imaging for quantitative analysis of micellar lymphatic transport based on SICTERS. Such a quantitative Raman imaging method achieved accurate measurement of dynamic micellar concentrations in lymphatics over time. A PBPK model was developed to calculate the pharmacokinetics of micellar lymphatic transport with different particle sizes and surface charges. To the best of our knowledge, this is the first report to use non‐invasively quantitative Raman imaging for PBPK modeling and calculation of lymphatic transport of nanoparticles that opens an avenue for Raman image‐based design and evaluation of lymph node‐targeted nanovaccines and delivery systems.

## 5 Experimental Section

### 5.1 Materials

The chemical reagents used in this study were purchased from Sinopharm Chemical Reagent Co., Ltd and Bide Pharmatech Co., Ltd. The solvents were purchased from Titan. 1,2‐Distearoyl‐sn‐glycero‐3‐phosphoethanolamine‐Polyethylene Glycol (DSPE‐PEG_2000_) was purchased from AVT Pharmaceutical Tech Co., Ltd. 1,2‐distearoyl‐sn‐glycero‐3‐phosphoethanolamine‐polyethyleneimine (DSPE‐PEG_2000_‐PEI_1800_) was purchased from SUNLipo NanoTech Co., Ltd. 1,2‐distearoyl‐sn‐glycero‐3‐phosphoethanolamine‐N‐[carboxy (polyethylene glycol)] (DSPE‐PEG_2000_‐COOH) was purchased from Yusiyi Pharmaceutical Technology Co., Ltd. CpG ODN 1826 was purchased from HongXun Biotechnology Co., Ltd. Ovalbumin (OVA) was purchased from Yuanye Bio‐Technology Co., Ltd. 4,7‐bis(4‐(2‐ethylhexyl)thiophen‐2‐yl)benzobis[*c*][1,2,5] thiadiazole (BBT) was synthesized according to our previously reported procedure [28].

### 5.2 Cell Lines and Animals

Murine melanoma B16‐F10 cells stably expressing OVA (B16‐OVA) were kindly provided by Dr. Fubin Li from Shanghai Jiao Tong University School of Medicine. Female C57BL/6 mice (6–8 weeks old) and female BALB/c mice (6–8 weeks old) were purchased from Shanghai Lingchang Biotech Co., Ltd. The mice were allowed to adapt to the environment for 1 week before the experiments and housed under specific pathogen‐free conditions with free access to food and water. All animal experiments were carried out in compliance with the guidelines established by the Institutional Animal Care and Use Committee (IACUC) of the School of Pharmacy, Fudan University.

### 5.3 Instruments

The nanoparticle size was measured by Zetasizer Nano ZS90 Laser Particle Size Analyzer (Malvern, UK). Raman spectra were collected by InVia Raman microscope (Renishaw, UK), which was equipped with 5 × and 20 × objective lens, 532 and 785 nm lasers, and a 1040 × 256 pixel charge‐coupled device detector. Fluorescence was determined by QM40 Steady‐State—Transient Fluorescence Spectrometer (PTI, USA). Immune cell flow cytometry analysis was determined by CytoFlex flow Cytometer (Beckman, USA). Absorption spectra were determined by Lambda 365 Ultraviolet‐Visible‐Near‐Infrared Spectrophotometer (PerkinElmer, USA).

### 5.4 Preparation of SICTER Micelles

PEI‐100 micelles: BBT (1 mg) and DSPE‐PEG_2000_ (0.7 mg) were dissolved in 0.5 mL tetrahydrofuran (THF). DSPE‐PEG_2000_‐PEI_1800_ (0.3 mg) was dissolved in 0.5 mL of methanol. The two solutions were mixed under ultrasonication (Scientz) and added to 9 mL of deionized water. The mixtures were then sonicated for 30 min at 20 W of the output power. After THF and methanol evaporation by stirring the mixture in fume hood for 8 h, the resulting SICTERS micelles were obtained by filtration through a 0.22‐µm filter and washed three times with an ultrafiltration tube (MWCO 100 kDa, Merck, Germany).

PEI‐170 micelles: BBT (1 mg) and DSPE‐PEG_2000_ (0.7 mg) were dissolved in 0.5 mL THF. DSPE‐PEG_2000_‐PEI_1800_ (0.3 mg) was dissolved in 0.5 mL of methanol. The two solutions were mixed under ultrasonication (Scientz) and added to 9 mL of deionized water. The mixtures were then sonicated for 10 min at 20 W of the output power. After THF and methanol evaporation by stirring the mixture in fume hood for 8 h, the resulting SICTERS micelles were obtained by filtration through a 0.22‐µm filter and washed three times with an ultrafiltration tube (MWCO 100 kDa, Merck, Germany).

Micellar vaccines: The PEI‐100 micelles were mixed with an equal volume of aqueous solution containing 0.4 mg of ovalbumin (OVA) and 0.1 mg of oligodeoxynucleotides containing CpG motifs (CpG ODN) for 10 min to obtain the micellar vaccines.

COOH micelles: BBT (1 mg) was dissolved in 0.5 mL THF. DSPE‐PEG_2000_‐COOH (1 mg) was dissolved in 0.5 mL of methanol. The two solutions were mixed under ultrasonication (Scientz) and added to 9 mL of deionized water. The mixtures were then sonicated for 20 min at 20 W of the output power. After THF and methanol evaporation by stirring the mixture in fume hood for 8 h, the resulting SICTERS micelles were obtained by filtration through a 0.22‐µm filter and washed three times with an ultrafiltration tube (MWCO 100 kDa, Merck, Germany).

### 5.5 Characterization of SICTERS Micelles

The particle size and zeta potential of SICTERS micelles were measured by a dynamic light scattering (DLS) instrument (Malvern Nanozetasizer, Worcester, UK). Morphology of these SICTERS micelles was observed by transmission electron microscope (FEI Tecnai G2 20 TWIN, Hillsboro, USA). The fluorescence spectrophotometer (PTI QM40, USA) absorbance was used to quantify the concentration of BBT in the micelles.

### 5.6 Raman Imaging

All Raman imaging measurements were performed by an inVia Raman microscope with a 1040 × 256 pixel charge‐coupled device detector (Renishaw, UK) with 785‐nm laser excitation, 5 × objective lens, and a laser power of 62.6 mW.

For Raman scattering signal detection of SICTERS micelle solution, 5 µL of SICTERS micellar solution was dropped on the glass slide covered with aluminum foil. The measurement was conducted with spectral acquisition accumulated three times and acquisition time of 0.3 s.

For the establishment of quantitative methods and standard curves for lymphatic transport Raman imaging, the PEI‐100 micellar solution was sealed in a glass capillary (inner diameter of 1 mm), which was covered with depilated mouse leg skin. Raman measurement was performed with a single accumulation mode, an acquisition time of 0.6 s, and a step size of 15 µm. A standard curve was obtained by fitting the Raman scattering intensity (894 cm^−1^) and different concentrations (mg/g) of SICTERS micelle solutions.

Repeated Raman imaging of a capillary glass tube containing PEI‐100 micelles covered with depilated mouse leg skin was performed for 8 cycles over a 4 h period. Each cycle consisted of 15 min of Raman imaging with laser irradiation followed by another 15 min without irradiation.

For in vivo Raman imaging of micellar lymphatic transport, 50 µL of BBT micellar solution (3 mg/mL BBT) was subcutaneously injected into the footpad of mice. The mice were anesthetized with isoflurane. The body temperature of the mice was maintained with a warming pad. After depilated, the mice were placed on the imaging stage for imaging at 15 min, 1 h, and 4 h, respectively. Since the duration of Raman imaging was 15 min for each image, the imaging stated at 7.5 min before and ended at 7.5 min after the above indicated time points. Raman measurements were performed with a single accumulation mode, a step size of 15 µm and an acquisition time of 0.6 s.

For the quantitative Raman imaging of SICTERS micelles at different injection doses of BBT in lymphatics, 50 µL of PEI‐100 micellar solution containing 25, 50, or 75 µg of BBT was subcutaneously injected into the footpad of mice, respectively. The in vivo Raman imaging was performed using the same method.

For continuously quantitative Raman imaging of micellar lymphatic transport, 50 µL of BBT micellar solution (3 mg/mL BBT) was subcutaneously injected into the footpad of mice. The mice were imaged with the same method at 30, 60, 90, 120, 150, 180, 210, and 240 min, respectively.

To measure the maximum imaging depth of SICTERS micelle in biological tissues, the porcine skin was sectioned into slices with a thickness of ∼ 0.4 mm each. Depilated mice were subcutaneously injected in the footpad of mice under anesthesia with PEI‐100 micellar solution (150 µg of BBT). Sixty minutes after injection, PLNs were surgically exposed and covered with various pieces of porcine skin slices. Raman measurement was performed with a single accumulation mode, an acquisition time of 0.6 s.

### 5.7 Determination of SICTERS Micellar Concentrations In Vivo Through the Raman Imaging

The Raman images were generated and analyzed by a signal to baseline algorithm (WiRE 4.3 software, Renishaw). In brief, each spectrum was first pre‐processed through baseline correction and cosmic ray removal followed by smoothing. A weighted least squares processing method was used for baseline correction, and a second order, 3‐point window Savitzky‐Golay algorithm was used for signal smoothing [87]. All the spectra were normalized to remove instrument error and make them comparable. The Raman intensity (894 cm^−1^) of pixels that exceeded the sensitivity (15 a.u.) in the location of lymphatic vessels or lymph nodes were delineated through bright‐field images and Raman images to determine the X and Y values of the tissue sites. The corresponding Raman spectra were exported from the image database. The Raman scattering signal value at 894 cm^−1^ was averaged that was substituted into the above standard curve to calculate the BBT concentration (mg/g).

To verify the BBT concentration, the above mice were euthanized after imaging. Lymph nodes were collected, weighted, and mixed with phosphate buffered saline (PBS) (20 times the tissue weight). The mixture was homogenized by a tissue homogenizer for 3 min. To extract BBT from the homogenate, toluene was added. The mixture was sonicated by ultrasonic cell crusher (Scientz) and centrifuged for 10 min at 21 130 × g. The supernatant was collected, and measured fluorescence intensity (Excitation = 830 nm, Emission = 1020 nm) for measurement of BBT actual concentration (mg/g) on a fluorescence spectrophotometer (PTI QM40, USA).

### 5.8 Physiologically Based Pharmacokinetic (PBPK) Model

After subcutaneous administration of the mouse foot, SICTERS micelles diffused into the afferent lymphatic vessels (ALVs), along which the micelles were drained to the popliteal lymph node (PLN). Subsequently, the micelles flowed out from the PLN through the efferent lymphatic vessels (ELVs). Due to the presence of valves in the lymphatic vessels, the lymphatic drainage is unidirectional [39, 40]. Assuming the amount of SICTERS micelles in PLN (*X*) follows the first‐order rate (linear) process, and each of ALV, PLN, and ELV is considered as separate compartment, a physiologically based pharmacokinetic model describing SICTERS micelles transport to the PLN is as follows:


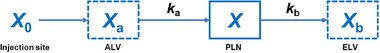
 where *X*_0_ is the dose amount of administration; *X*_a_ is the amount of micelles in the ALV; *k*_a_ is the influx rate constant; *X* is the amount of micelles in the PLN; *k*_b_ is the efflux rate constant, *X*_b_ is the amount of micelles in the ELV; *C* is the concentration of micelles in the PLN.

The rate of change of *X* (the amount of SICTERS micelles in PLN) can be calculated from the following differential equation:

(3) $$\frac{dX}{dt}=k_{a}\;X_{a}-k_{b}X$$

Taking the Laplace transform, it yields:

(4) $$S\;\bar{X}=k_{a}\;\bar{X_{a}}-k_{b}\bar{X}$$

The rate of change of *X*_a_ is proportional to *X*_a_:

(5) $$\frac{dX_{a}}{dt}=-k_{a}X_{a}$$

Taking the Laplace transform, it yields:

(6) $$S\bar{X_{a}}-F\;X_{0}=-k_{a}\bar{X_{a}}$$

where *F* is the fraction of the dosage of administration absorbed into the ALVs. Because subcutaneous absorption may be incomplete, the bioavailable amount from the injection site is the product of *X*_0_ and *F*. By solving $\bar{Xa}$ in Equation (6) and substituting it into Equation (4), the following equation is obtained:

(7) $$\bar{X}=\frac{k_{a}FX_{0}}{\left(S+k_{b}\right)\left(S+k_{a}\right)}\;$$

Applying the inverse Laplace transform to Equation (7), it gives the following equation:

(8) $$X=\frac{k_{a}FX_{0}}{k_{a}-k_{b}}\;\left(e^{-k_{b}t}-e^{-k_{a}t}\right)$$

To convert the amount *X*(*t*) into concentration *C*(*t*) within the PLN by dividing *X*(*t*) by the PLN volume (*V*), it gives the following equation:

(9) $$C=\frac{k_{a}FX_{0}}{V\left(k_{a}-k_{b}\right)}\;\left(e^{-k_{b}t}-e^{-k_{a}t}\right)$$

Equation (9) can be further simplified to the following equation:

(10) $$C=A\left(e^{-k_{b}t}-e^{-k_{a}t}\right)$$

where *A* is constant.

The nonlinear equation of the concentration over time data was fitted using MATLAB (R2021b). Based on the nonlinear least square method, the “LsqCurvefit” function was utilized to obtain the required fitting parameters and goodness‐of‐fit of Equation (10).

### 5.9 Experiment of Immunization

For the mice bearing B16‐OVA model, C57BL/6 mice were inoculated with 5 × 10^5^ of B16‐OVA cells subcutaneously on the right side of the abdomen at Day 0. (1) Micellar vaccine treatment group: C57BL/6 mice were injected with 50 µL of micellar vaccine containing 100 µg of OVA and 25 µg of CpG ODN subcutaneously into the footpad of mice at Day 6, 13 and 20, respectively. (2) OVA & CpG solution positive control group: C57BL/6 mice were injected subcutaneously with 50 µL of aqueous solutions containing 100 µg of OVA and 25 µg of CpG ODN into the footpad of mice at Day 6, 13, and 20, respectively. (3) PBS control: C57BL/6 mice were injected subcutaneously with 50 µL of PBS solution into the footpad of mice at Day 6, 13, and 20, respectively. The tumor sizes were measured every 3 days by using electronic calipers.

### 5.10 Flow Cytometry Analysis of Immune Cells

The vaccinated mice were euthanized at Day 21 after tumor inoculation. Tumor tissues, spleens, and lymph nodes were collected. The tissues were processed into single‐cell suspensions according to previous procedure [91]. The cells were stained with antibodies shown in Table S1 according to the manufacture's instruction and analyzed using a flow cytometer.

### 5.11 Biosafety Evaluation

Safety evaluation of mice following repeated noninvasive Raman imaging according to the Method Experiment of Raman imaging, which involved 8 cycles, each cycle consisting of 15 min of Raman imaging followed by another 15 min without irradiation. After each Raman imaging, the skin of the mouse's leg was photographed, and the surface temperature of the skin was measured using an infrared thermal imaging camera. At 240 min, the mice were euthanized and skin, PLNs, ALVs and ELVs were collected for sectioning and H&E staining.

Anti‐tumor safety of micellar vaccines. The healthy BALB/c mice received same immunization regimen according to the Method Experiment of immunization. The body weight of each group of mice was monitored and recorded every 3 days after tumor inoculation. At Day 15 post‐primary immunization, the mice were euthanized and hearts, livers, spleens, lungs, and kidneys were collected for sectioning and H&E staining. Blood was collected for hematologic analysis and serum biochemistry analysis.

### 5.12 Statistical Analysis

All statistical differences were calculated using Graphpad Prism 7.0 software (San Diego, USA). Data were presented as mean ± standard deviation (SD) for all results. Statistical significance was determined by using a two‐tailed Student's *t*‐test, one‐way analysis of variance (ANOVA) with Tukey's multiple comparisons post‐hoc test, two‐way analysis of variance (ANOVA) with Tukey's multiple comparisons post‐hoc test. The Kaplan‐Meier method was used to analyze the differences in animal survival. The curves were compared with the log‐rank (Mantel‐Cox) test. Statistical significant difference was defined as *p < 0.05, **p < 0.01, ***p < 0.001, ****p < 0.0001. ns, no significant difference.

## Author Contributions

**Yunlu Li**: methodology, software, data curation, investigation, validation, conceptualization, formal analysis, visualization, writing – original draft, writing – review and editing. **Hongzheng Lin**: methodology, software, formal analysis. **Sheng Yu**: visualization, software. **Yunhui Liao**: methodology, formal analysis. **Teng Huang**: investigation, validation. **Zeyu Xiao**: conceptualization, project administration, resources, funding acquisition, writing – review and editing. **Wei Lu**: conceptualization, funding acquisition, writing – review and editing, project administration, resources, supervision.

## Funding

This work was supported by the National Natural Science Foundation of China (22595413, 22595411, 22595410 and 81991493).

## Conflicts of Interest

The authors declare no conflicts of interest.

## Supporting information




**Supporting File**: advs77634‐sup‐0001‐SuppMat.pdf.

## Data Availability

The data that supports the findings of this study are available in the supplementary material of this article.
