# Supplementary material for: Non‐Invasively Quantitative Raman Imaging for Calculation of Lymphatic Transport Kinetics of Micelles in Mice
Source: Adv Sci (Weinh). 2026 Sep 8:e77634. Online ahead of print. doi: 10.1002/advs.77634 (PMC13554503; doi:10.1002/advs.77634)
Supplement: Supplementary file 1 [file ADVS-9999-e77634-s001.pdf]

Supporting Information

Non-Invasively Quantitative Raman Imaging for Calculation of Lymphatic Transport Kinetics of Micelles in Mice

Yunlu Li[a], Hongzheng Lin[a], Sheng Yu[a], Yunhui Liao[a], Teng Huang[a], Zeyu Xiao[b,c,], Wei Lu[a,d,*]*

[a]School of Pharmaceutical Sciences, Minhang Hospital, Key Laboratory of Smart Drug Delivery Ministry of Education, State Key Laboratory of Molecular Engineering of Polymers, Fudan University, Shanghai 201203, China.

[b]Department of Pharmacology and Chemical Biology, Institute of Molecular Medicine, Shanghai Jiao Tong University School of Medicine, Shanghai 200025, China.

[c]Hangzhou Institute of Medicine (HIM), Chinese Academy of Sciences, Hangzhou Zhejiang 310022, China.

[d]Quzhou Fudan Institute, Quzhou, Zhejiang 324002, China

*Corresponding authors: Zeyu Xiao and Wei Lu

Email: zxiao@sjtu.edu.cn (Z.X.), wlu@fudan.edu.cn (W.L.)

Figures

a

	PEI-100 micelle	PEI-170 micelle	COOH micelle
Diameter (nm)	97.28 ± 2.11	167.67 ± 3.58	93.85 ± 3.29
PDI	0.14 ± 0.06	0.16 ± 0.01	0.15 ± 0.02
Zeta-potential (mV)	11.47 ± 0.61	9.97 ± 0.61	-32.13 ± 1.1
BBT encapsulation efficiency(%)	56.79 ± 3.33	57.12 ± 1.00	76.36 ± 4.8

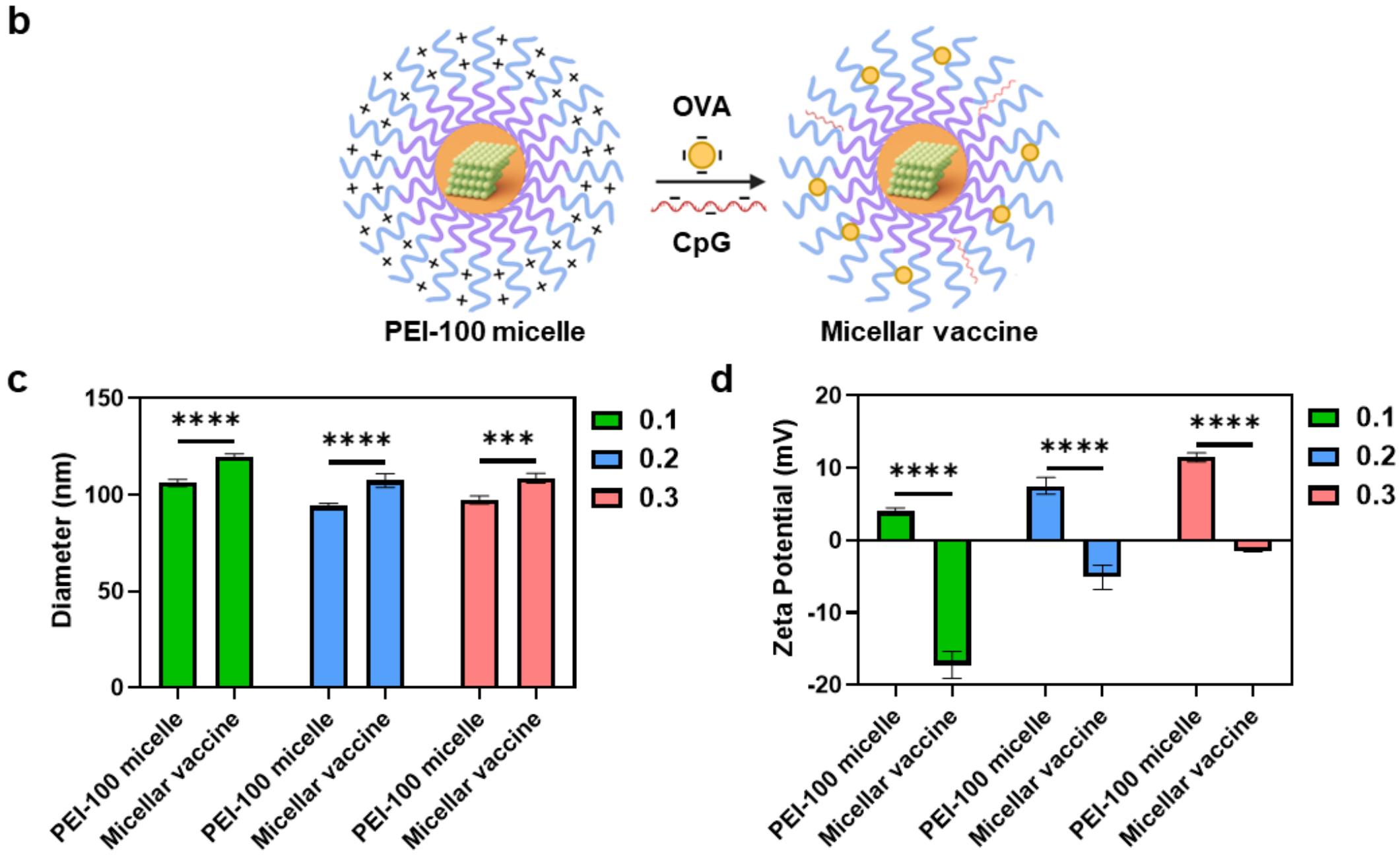

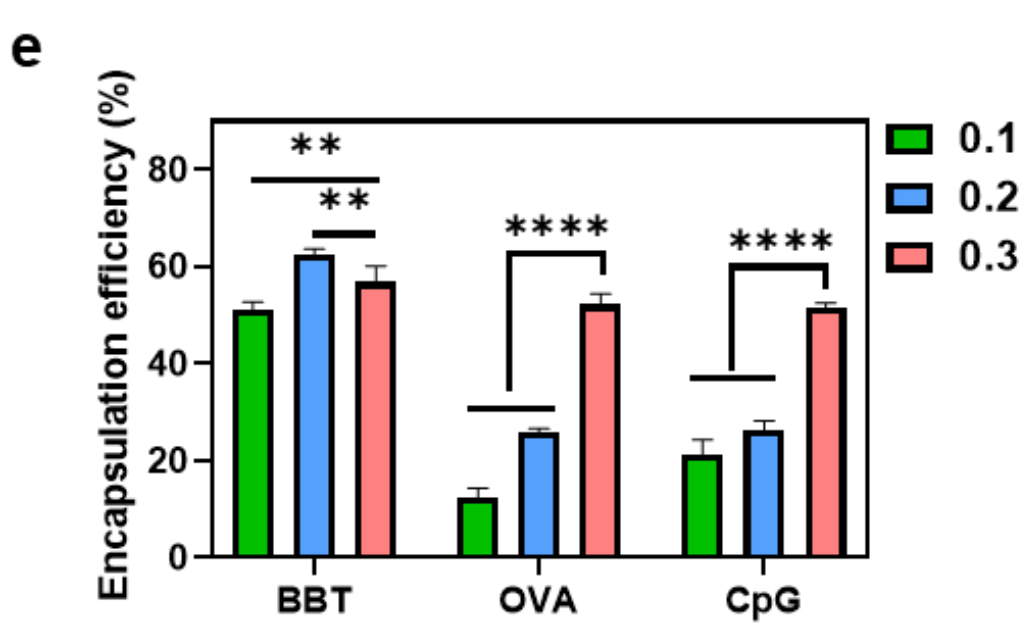

f

	Micellar vaccine (DSPE-PEG-PEI = 0.3)
Diameter (nm)	107.37 ± 3.40
PDI	0.12 ± 0.04
Zeta-potential (mV)	-1.5 ± 0.08
BBT encapsulation efficiency (%)	56.79 ± 3.33
OVA encapsulation efficiency (%)	52.15 ± 2.23
CpG encapsulation efficiency (%)	51.53 ± 0.91

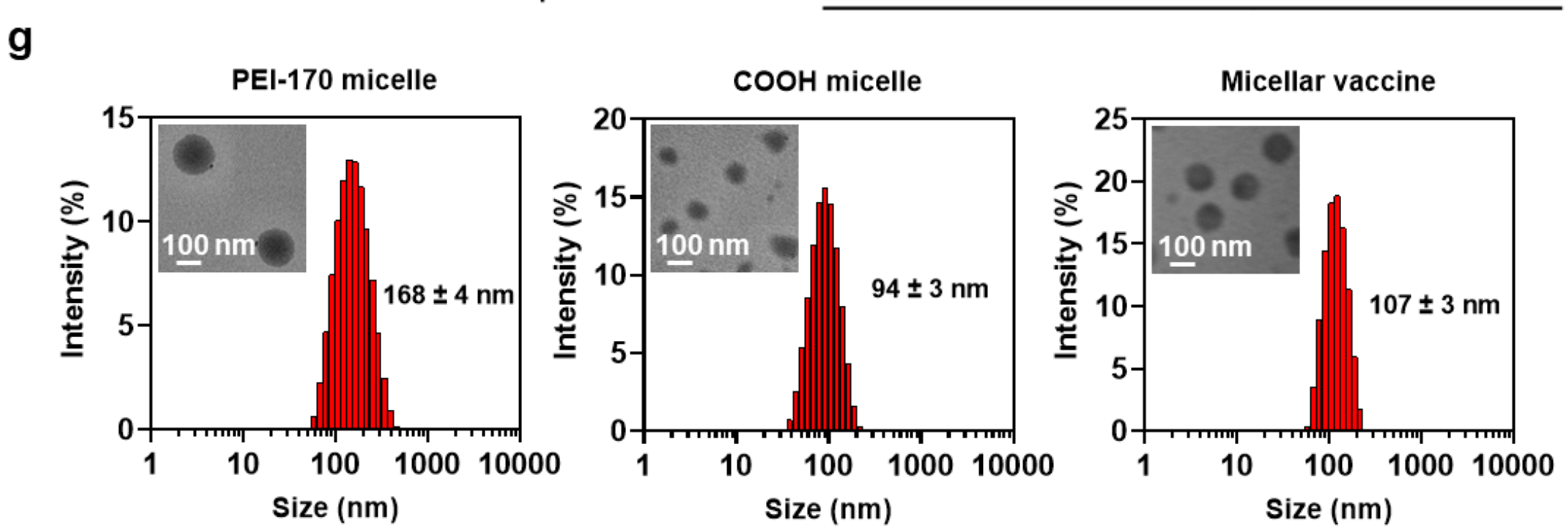

Figure S1. Characterization of SICTERS micelles and micellar vaccines. (a) Diameter, PDI, Zeta-potential and BBT encapsulation efficiency of different SICTERS micelles,

respectively. (b) Schematic diagram of micellar vaccines loading OVA and CpG. (c) The particle size of PEI-100 micelles and micellar vaccines prepared at different ratios of DSPE-mPEG$_{2000}$-PEI$_{1800}$ to total lipid mass. (d) The Zeta potential of PEI-100 micelles and micellar vaccines prepared at different ratios of DSPE-mPEG$_{2000}$-PEI$_{1800}$ to total lipid mass. (e) The encapsulation efficiency of BBT, OVA and CpG ODN of micellar vaccines prepared at different ratios of DSPE-mPEG$_{2000}$-PEI$_{1800}$ to total lipid mass. (f) Diameter, PDI, Zeta-potential and encapsulation efficiency of micellar vaccines when the ratio of DSPE-mPEG$_{2000}$-PEI$_{1800}$ to total lipid mass was 0.3. (g) Size distribution of different SICTERS micelles and micellar vaccines. Data are expressed as mean ± SD (n = 3). $^{*}P < 0.05$, $^{**}P < 0.01$, $^{***}P < 0.001$, $^{****}P < 0.0001$ between the compared groups analyzed by two-way ANOVA with Tukey's post-hoc test.

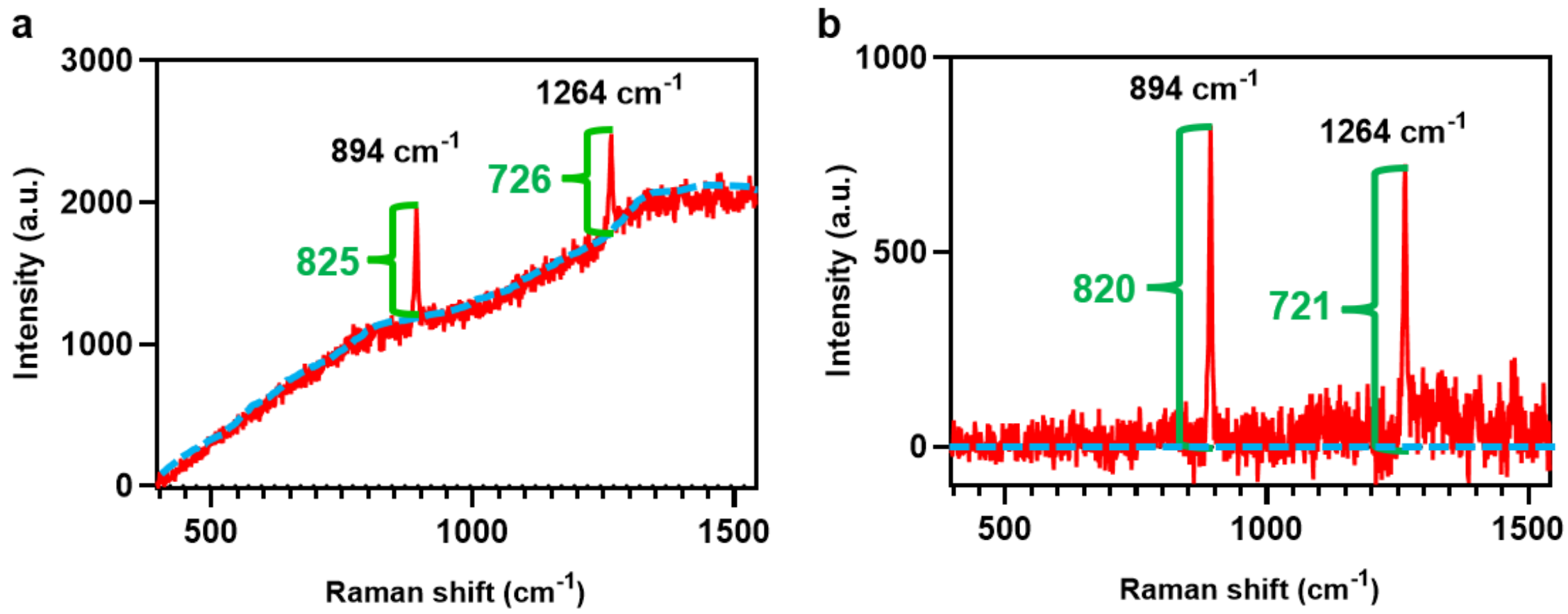

Figure S2. Representative raw (a) and baseline-corrected (b) Raman spectra of PEI-100 micelle solution (2 μg/mL). Blue dashed lines represent the baseline. Green brackets indicate the peak Raman intensity relative to the baselines at 894 cm^{-1} and 1264 cm^{-1}, respectively. Raman measurement was carried out with a 5 × objective, 785-nm laser excitation, a laser power of 62.6 mW, acquisition time of 0.3 s and 3 times accumulation.

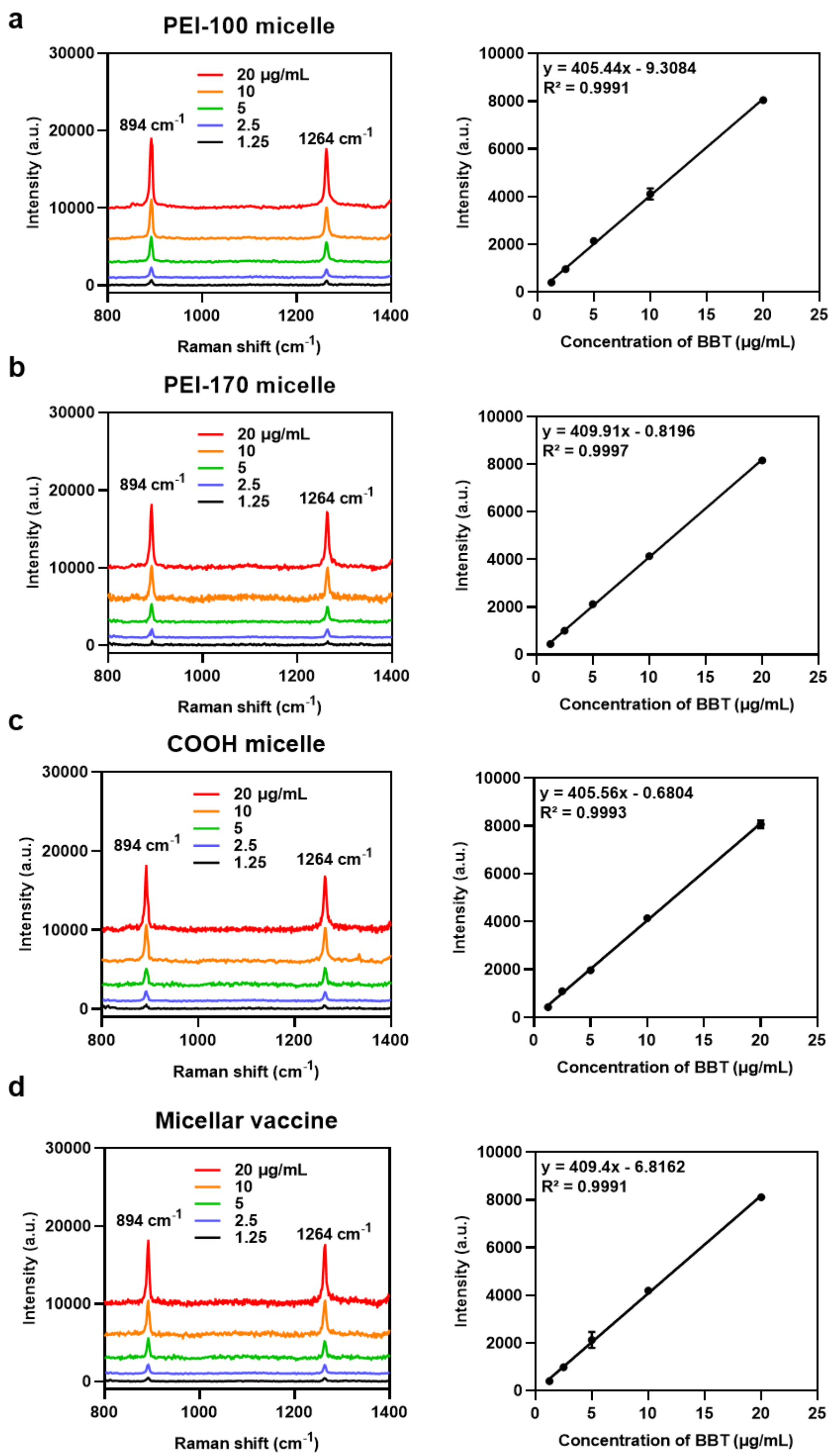

Figure S3. Raman intensity of SICTERS micelles. (a) Raman spectra of PEI-100 micelles at different concentrations of BBT and linear regression curve between Raman intensity (894 cm^{-1}) and PEI-100 micelles at different concentrations of BBT. (b) Raman spectra of PEI-170 micelles at different concentrations of BBT and linear regression curve between

Raman intensity (894 cm^{-1}) and PEI-170 micelles at different concentrations of BBT. (c) Raman spectra of COOH micelles at different concentrations of BBT and linear regression curve between Raman intensity (894 cm^{-1}) and COOH micelles at different concentrations of BBT. (d) Raman spectra of micellar vaccines at different concentrations of BBT and linear regression curve between Raman intensity (894 cm^{-1}) and micellar vaccines at different concentrations of BBT. Raman measurement was carried out with 785-nm laser excitation, a ×5 objective, a laser power of 62.6 mW, three times accumulation and an acquisition time of 0.3 s. Data are expressed as mean ± SD (n = 3).

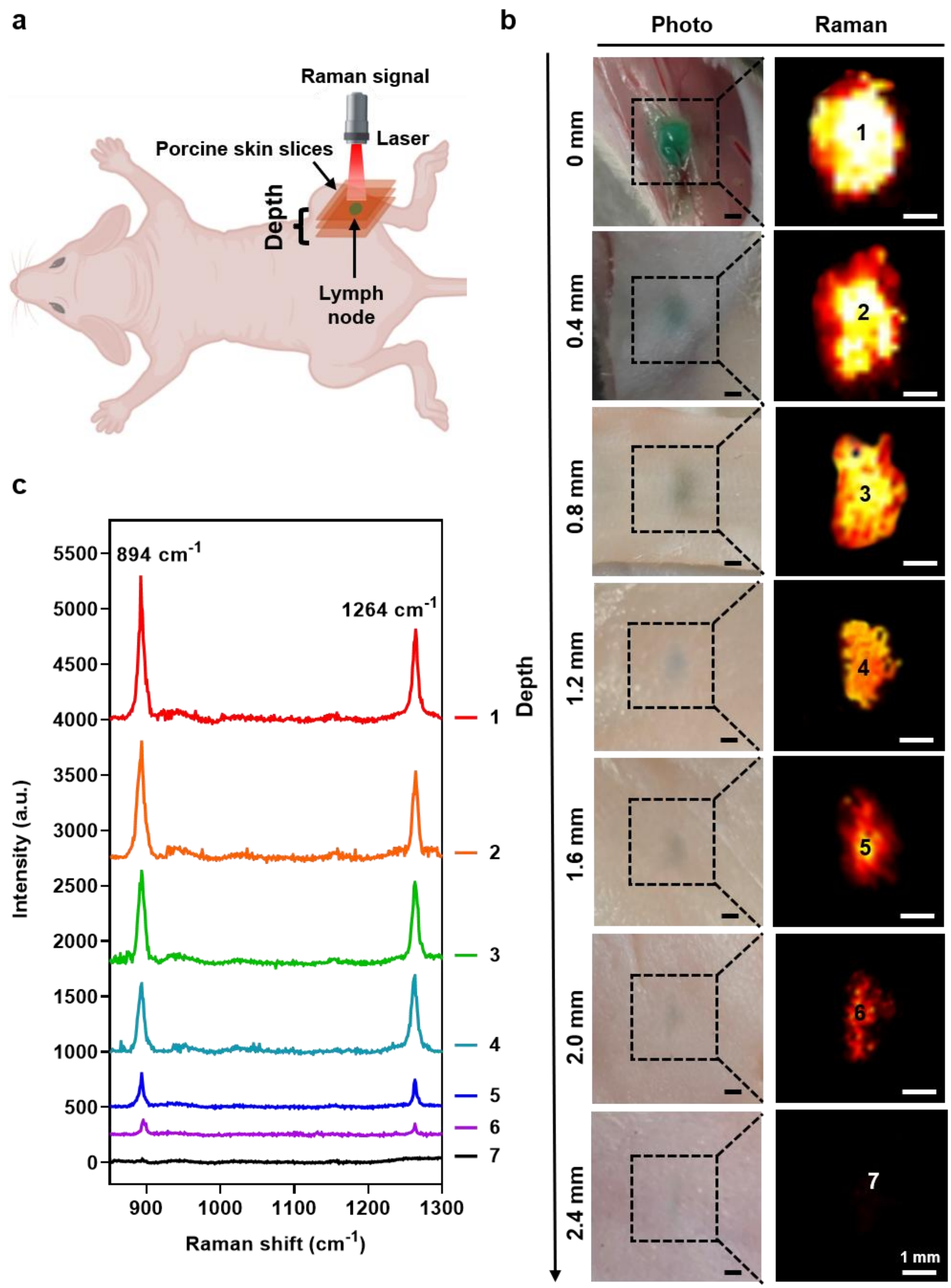

Figure S4. Depth analysis of SICTERS imaging by covering BBT micelles-bearing PLN with porcine skin slices. (a) Illustration of the experimental setup. PEI-100 micelles (150 μg of BBT) were subcutaneous injected through the footpad of mice. At 60 min after injection, PLN was exposed and then covered with different pieces of porcine skin slices

for Raman imaging. The thickness of each piece of porcine skin slice was 0.4 mm. (b) The SICTERS-based Raman imaging (894 cm^{-1}) of BBT micelles-bearing PLNs, covered with porcine skin slices with thicknesses of 0.4 mm, 0.8 mm, 1.2 mm, 1.6 mm, 2.0 mm or 2.4 mm, or without porcine skin slice (0 mm). (c) Raman spectra of different number-labeled regions in (b). Raman measurement was carried out with a 5 × objective, 785-nm laser excitation, a laser power of 62.6 mW, acquisition time of 0.6 s and one time accumulation.

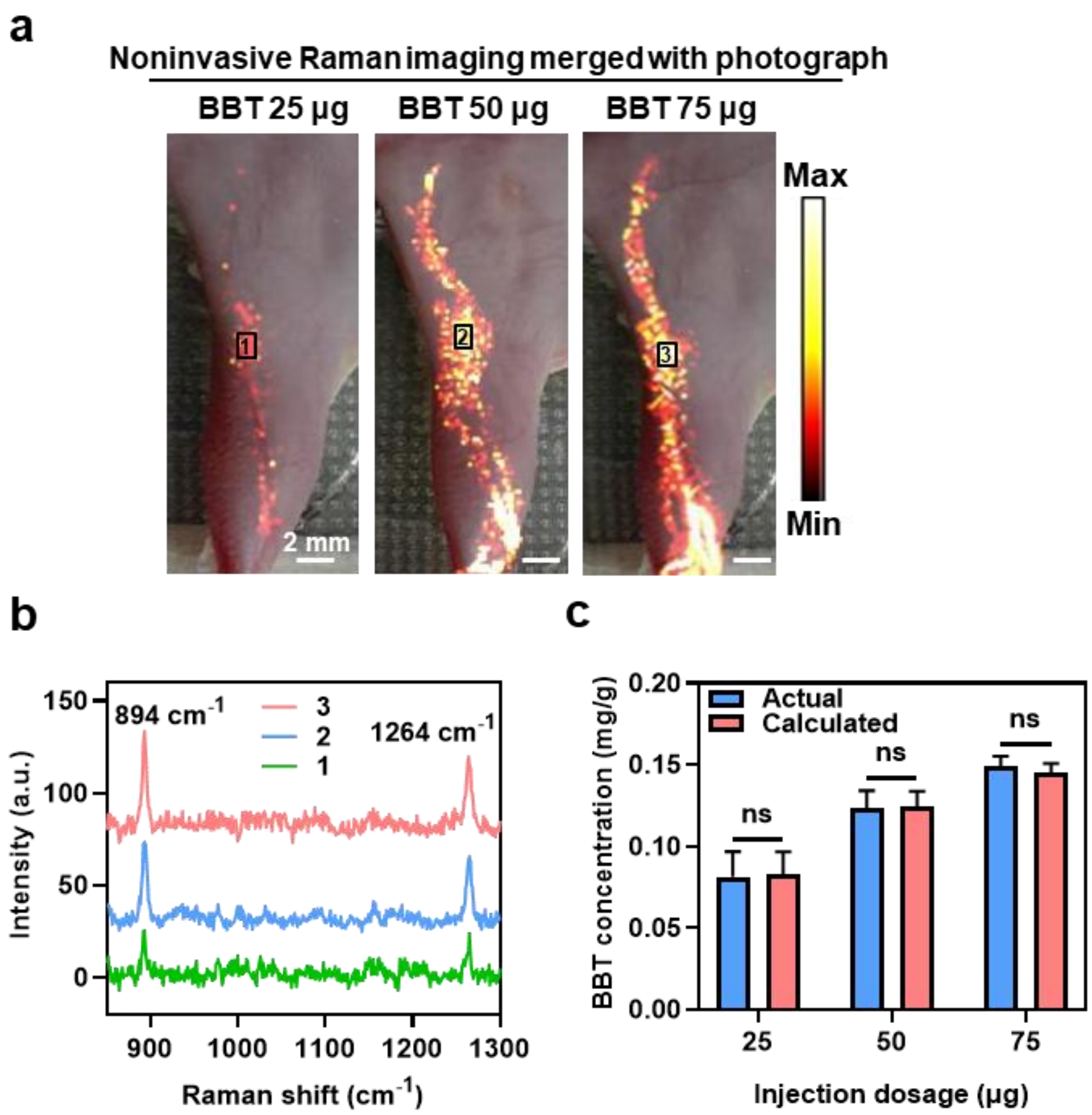

Figure S5. The quantitative Raman imaging of PEI-100 micelles at different injection doses of BBT in lymphatics. (a) The mice were subcutaneously injected with 50 µL of solution containing 25 µg, 50 µg and 75 µg BBT micelles, respectively. Non-invasive Raman imaging (894 cm^{-1}) was performed at 15 min post injection. (b) Raman spectra of the numbered regions in (a). (c) The comparison of BBT concentrations of PLNs between the calculated values and the actual ones following subcutaneous injection at different doses. The calculated BBT concentration was acquired by averaging the Raman scattering intensity (894 cm^{-1}) of all the pixels in the PLN in (a) followed by calculation using the standard curve in Figure 2f. The actual concentration was measured by fluorescence analysis of the extracts from the PLN homogenate following the Raman imaging. Raman measurement was carried out with 785-nm laser excitation, a ×5 objective, a laser power of 62.6 mW, one time accumulation and an acquisition time of 0.6 s. Data are expressed as mean ± SD (n = 3). ns, no significant difference between the compared groups analyzed by two-way ANOVA with Tukey's post-hoc test.

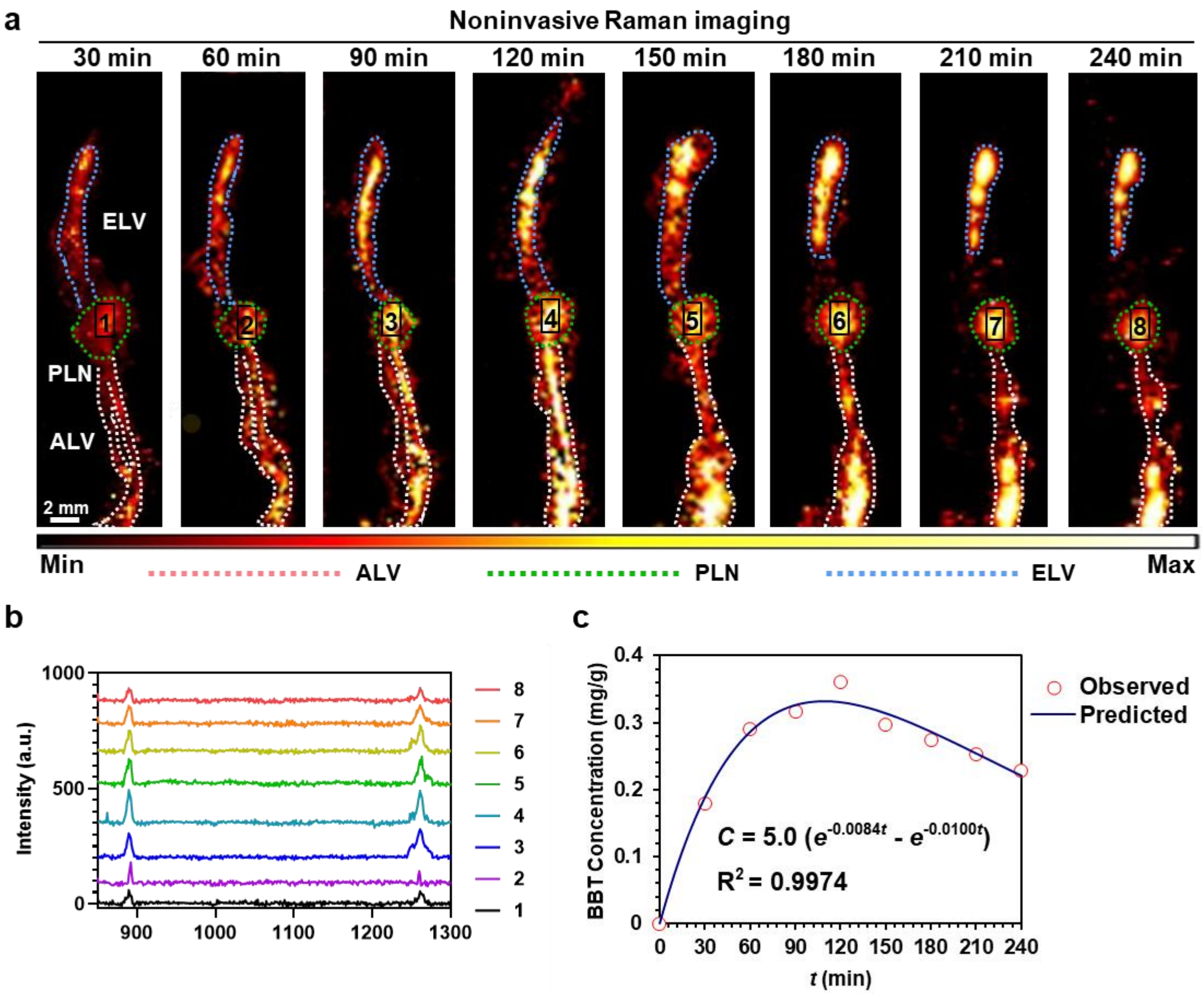

Figure S6. Continuously quantitative SICTERS imaging of Mouse 2 of PEI-100 micelles and the pharmacokinetics of micellar lymphatic transport via the PBPK model. (a) The non-invasive *in vivo* Raman imaging (894 cm^{-1}) was conducted at different time points after subcutaneous injection of 50 μL of PEI-100 micelles (3 mg/mL) into the mouse footpad. (b) Raman spectra of the numbered regions in the PLN in (a). (c) The concentration-time curves (predicted) of PEI-100 micelles in PLN by fitting the data of observed concertation at each time to Eq (**2**). Raman measurement was carried out with 785-nm laser excitation, a ×5 objective, a laser power of 62.6 mW, one time accumulation and an acquisition time of 0.6 s.

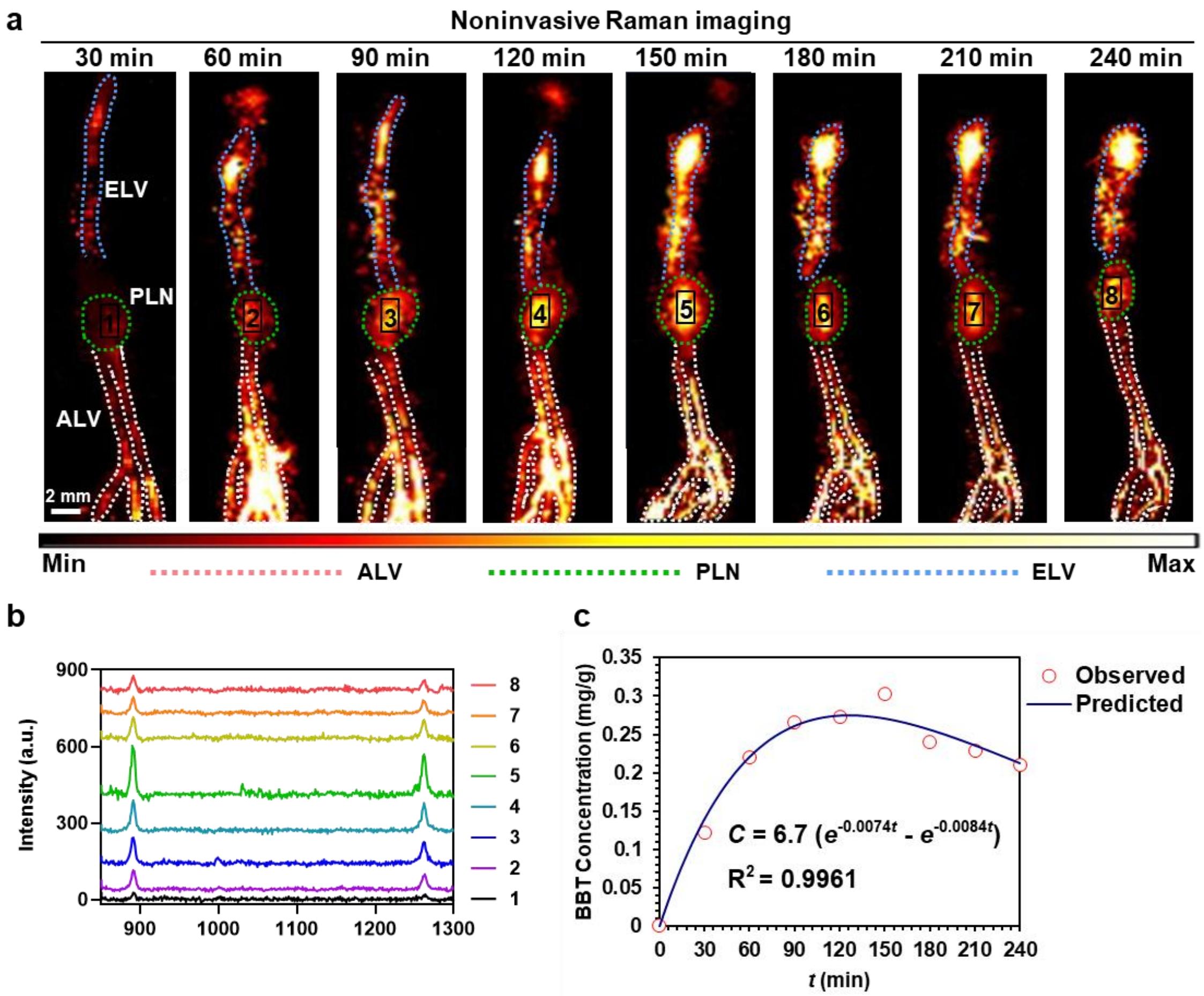

Figure S7. Continuously quantitative SICTERS imaging of Mouse 3 of PEI-100 micelles and the pharmacokinetics of micellar lymphatic transport via the PBPK model. (a) The non-invasive *in vivo* Raman imaging (894 cm^{-1}) was conducted at different time points after subcutaneous injection of 50 μL of PEI-100 micelles (3 mg/mL) into the mouse footpad. (b) Raman spectra of the numbered regions in the PLN in (a). (c) The concentration-time curves (predicted) of PEI-100 micelles in PLN by fitting the data of observed concentration at each time to Eq (**2**). Raman measurement was carried out with 785-nm laser excitation, a ×5 objective, a laser power of 62.6 mW, one time accumulation and an acquisition time of 0.6 s.

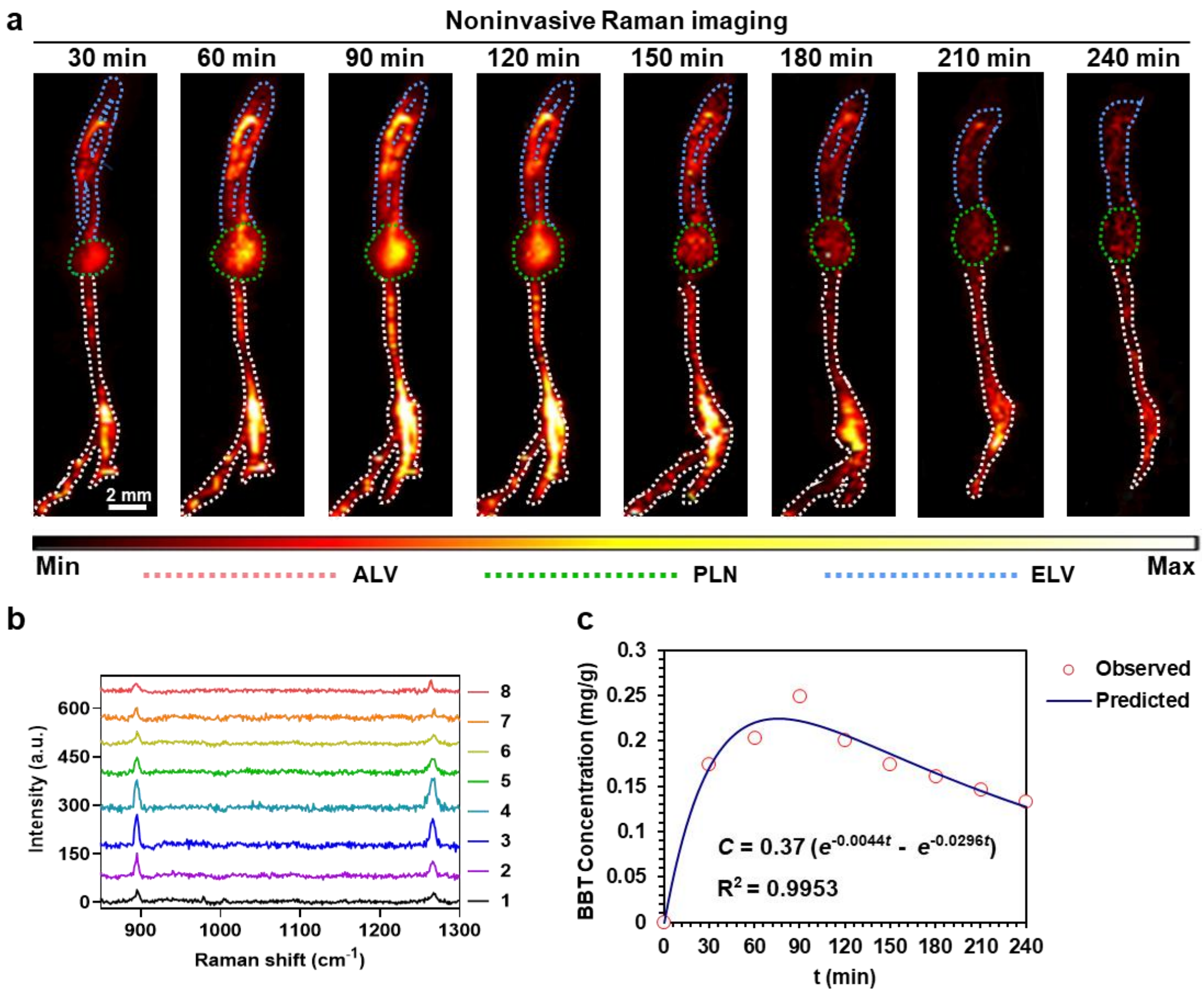

Figure S8. Continuously quantitative SICTERS imaging of Mouse 4 of PEI-170 micelles and the pharmacokinetics of micellar lymphatic transport via the PBPK model. (a) The non-invasive *in vivo* Raman imaging (894 cm^{-1}) was conducted at different time points after subcutaneous injection of 50 μL of PEI-170 micelles (3 mg/mL) into the mouse footpad. (b) Raman spectra of the numbered regions in the PLN in (a). (c) The concentration-time curves (predicted) of PEI-170 micelles in PLN by fitting the data of observed concentration at each time to Eq (**2**). Raman measurement was carried out with 785-nm laser excitation, a ×5 objective, a laser power of 62.6 mW, one time accumulation and an acquisition time of 0.6 s.

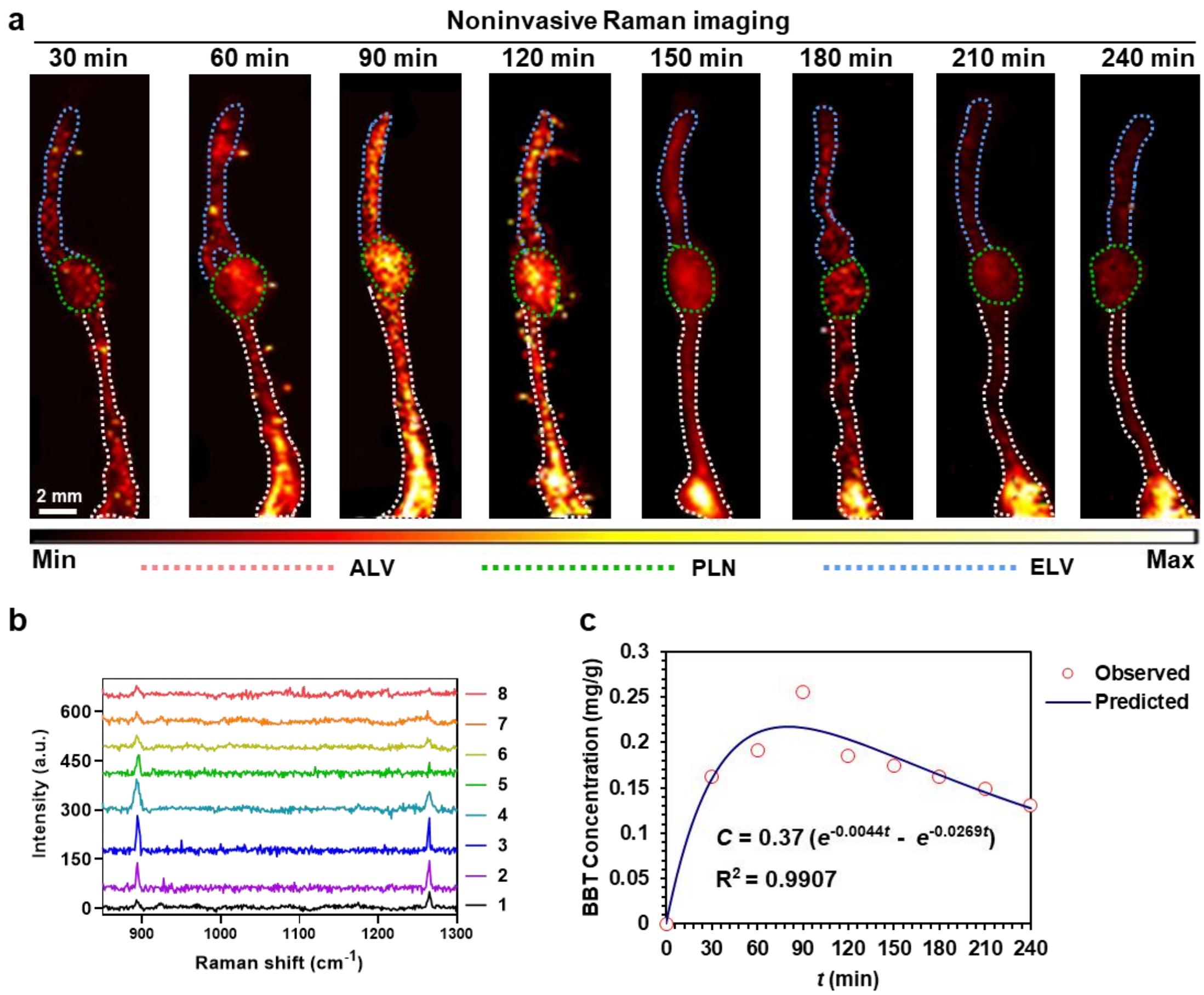

Figure S9. Continuously quantitative SICTERS imaging of Mouse 5 of PEI-170 micelles and the pharmacokinetics of micellar lymphatic transport via the PBPK model. (a) The non-invasive *in vivo* Raman imaging (894 cm^{-1}) was conducted at different time points after subcutaneous injection of 50 µL of PEI-170 micelles (3 mg/mL) into the mouse footpad. (b) Raman spectra of the numbered regions in the PLN in (a). (c) The concentration-time curves (predicted) of PEI-170 micelles in PLN by fitting the data of observed concentration at each time to Eq (**2**). Raman measurement was carried out with 785-nm laser excitation, a ×5 objective, a laser power of 62.6 mW, one time accumulation and an acquisition time of 0.6 s.

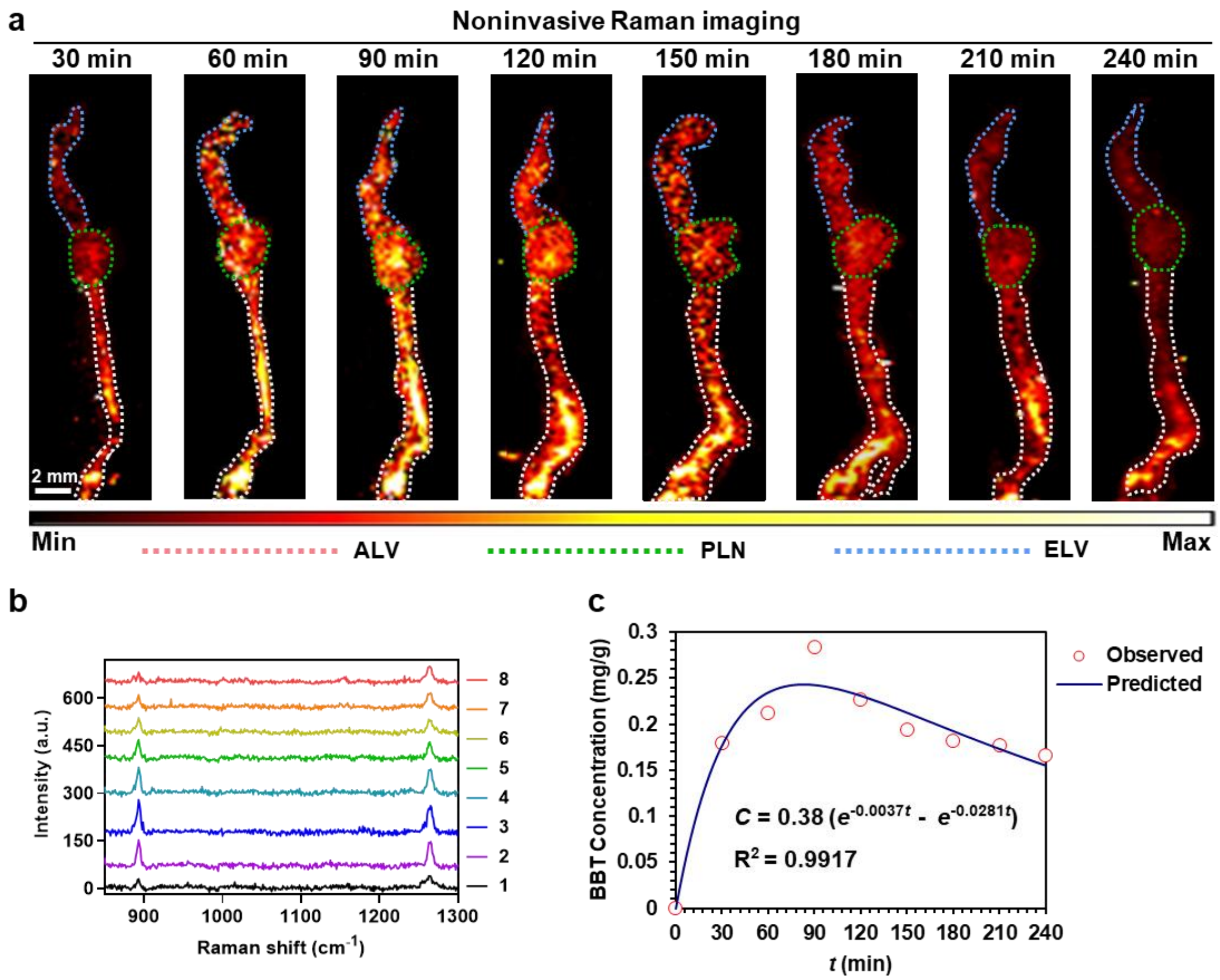

Figure S10. Continuously quantitative SICTERS imaging of Mouse 6 of PEI-170 micelles and the pharmacokinetics of micellar lymphatic transport via the PBPK model. (a) The non-invasive *in vivo* Raman imaging (894 cm^{-1}) was conducted at different time points after subcutaneous injection of 50 μL of PEI-170 micelles (3 mg/mL) into the mouse footpad. (b) Raman spectra of the numbered regions in the PLN in (a). (c) The concentration-time curves (predicted) of PEI-170 micelles in PLN by fitting the data of observed concentration at each time to Eq (**2**). Raman measurement was carried out with 785-nm laser excitation, a ×5 objective, a laser power of 62.6 mW, one time accumulation and an acquisition time of 0.6 s.

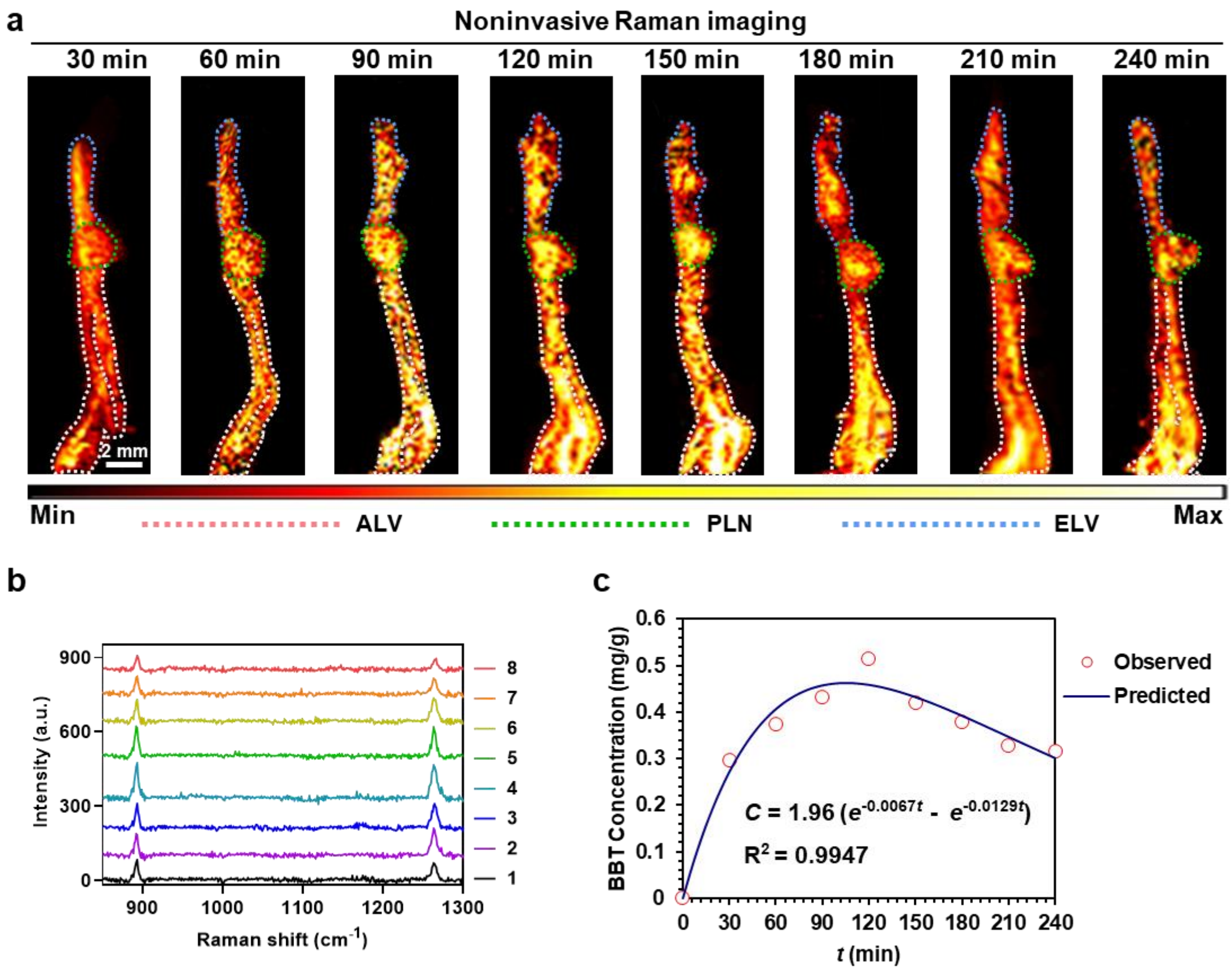

Figure S11. Continuously quantitative SICTERS imaging of Mouse 7 of COOH micelles and the pharmacokinetics of micellar lymphatic transport via the PBPK model. (a) The non-invasive *in vivo* Raman imaging (894 cm^{-1}) was conducted at different time points after subcutaneous injection of 50 μL of COOH micelles (3 mg/mL) into the mouse footpad. (b) Raman spectra of the numbered regions in the PLN in (a). (c) The concentration-time curves (predicted) of COOH micelles in PLN by fitting the data of observed concentration at each time to Eq (**2**). Raman measurement was carried out with 785-nm laser excitation, a ×5 objective, a laser power of 62.6 mW, one time accumulation and an acquisition time of 0.6 s.

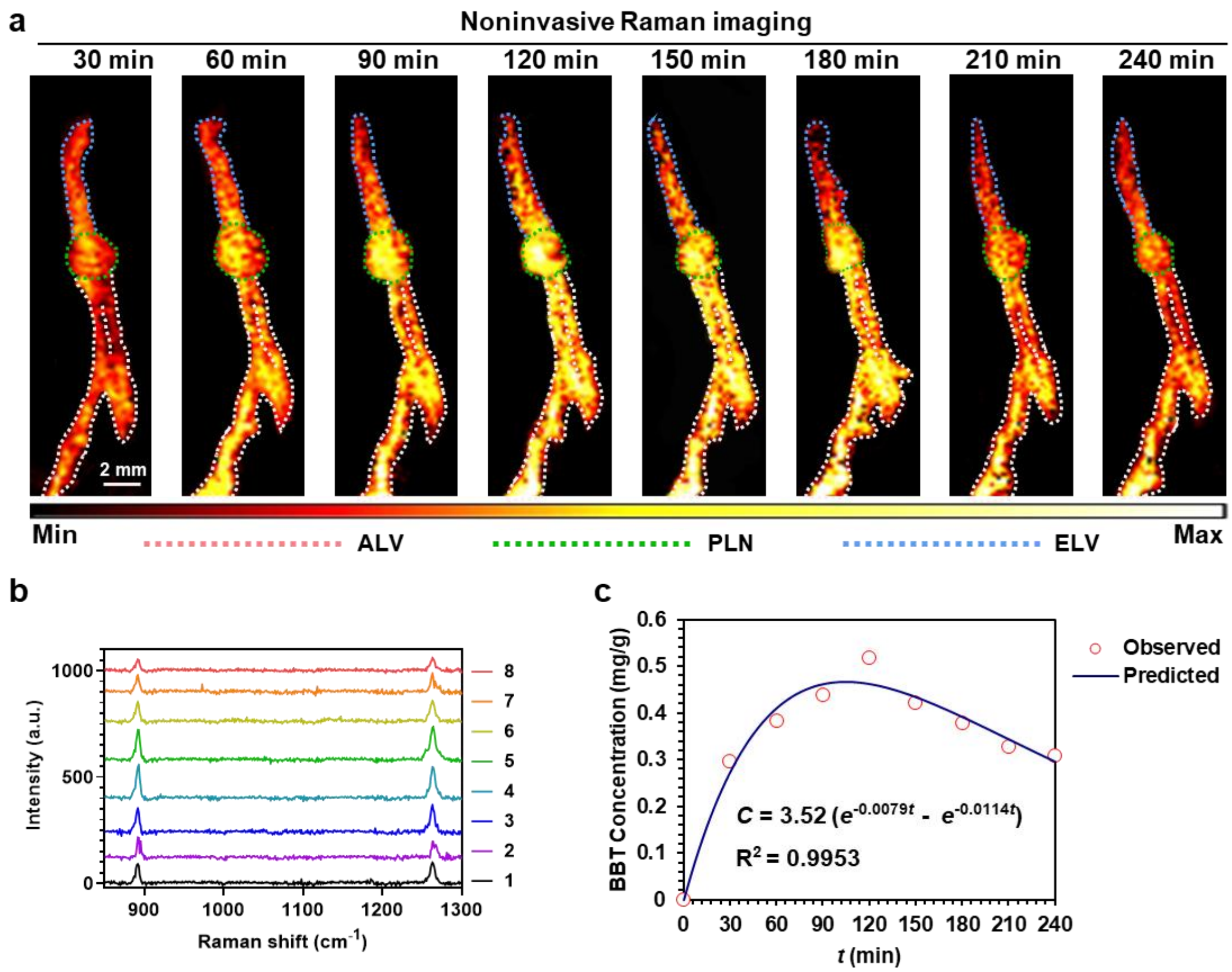

Figure S12. Continuously quantitative SICTERS imaging of Mouse 8 of COOH micelles and the pharmacokinetics of micellar lymphatic transport via the PBPK model. (a) The non-invasive *in vivo* Raman imaging (894 cm^{-1}) was conducted at different time points after subcutaneous injection of 50 μL of COOH micelles (3 mg/mL) into the mouse footpad. (b) Raman spectra of the numbered regions in the PLN in (a). (c) The concentration-time curves (predicted) of COOH micelles in PLN by fitting the data of observed concentration at each time to Eq (**2**). Raman measurement was carried out with 785-nm laser excitation, a ×5 objective, a laser power of 62.6 mW, one time accumulation and an acquisition time of 0.6 s.

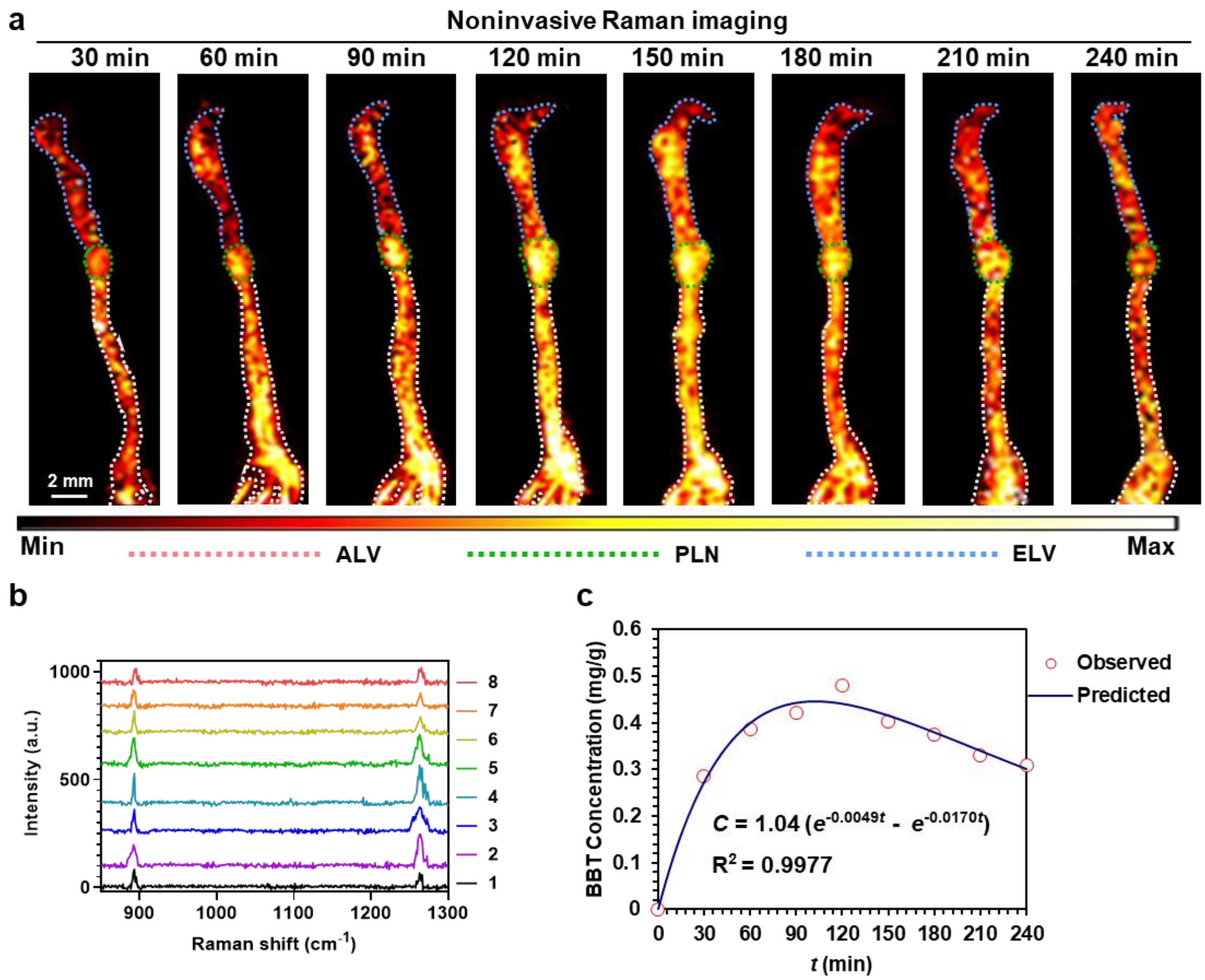

Figure S13. Continuously quantitative SICTERS imaging of Mouse 9 of COOH micelles and the pharmacokinetics of micellar lymphatic transport via the PBPK model. (a) The non-invasive *in vivo* Raman imaging (894 cm^{-1}) was conducted at different time points after subcutaneous injection of 50 μL of COOH micelles (3 mg/mL) into the mouse footpad. (b) Raman spectra of the numbered regions in the PLN in (a). (c) The concentration-time curves (predicted) of COOH micelles in PLN by fitting the data of observed concentration at each time to Eq (**2**). Raman measurement was carried out with 785-nm laser excitation, a ×5 objective, a laser power of 62.6 mW, one time accumulation and an acquisition time of 0.6 s.

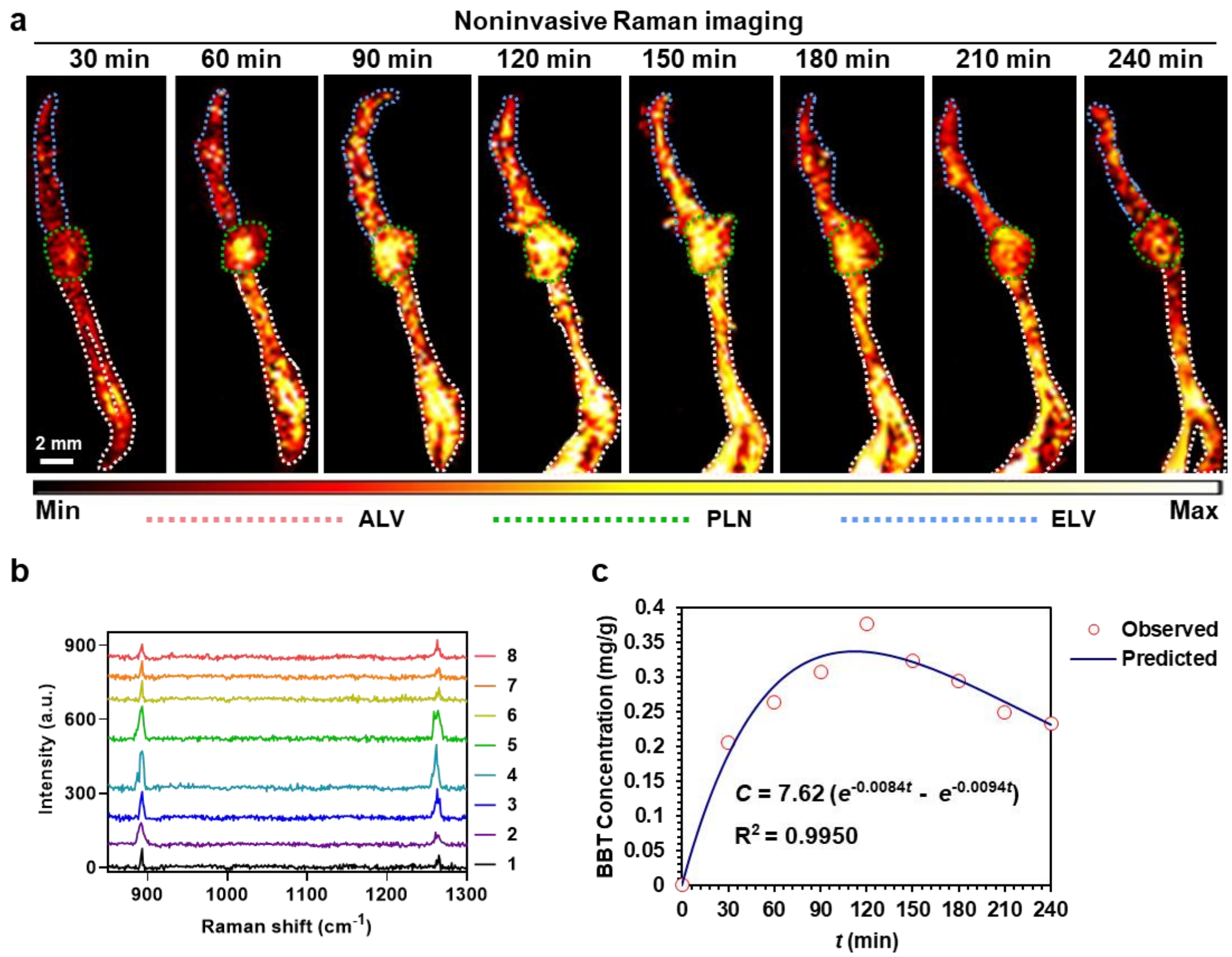

Figure S14. Continuously quantitative SICTERS imaging of Mouse 10 of micellar vaccines and the pharmacokinetics of micellar lymphatic transport via the PBPK model. (a) The non-invasive *in vivo* Raman imaging (894 cm^{-1}) was conducted at different time points after subcutaneous injection of 50 µL of micellar vaccines (3 mg/mL) into the mouse footpad. (b) Raman spectra of the numbered regions in the PLN in (a). (c) The concentration-time curves (predicted) of micellar vaccines in PLN by fitting the data of observed concentration at each time to Eq (**2**). Raman measurement was carried out with 785-nm laser excitation, a ×5 objective, a laser power of 62.6 mW, one time accumulation and an acquisition time of 0.6 s.

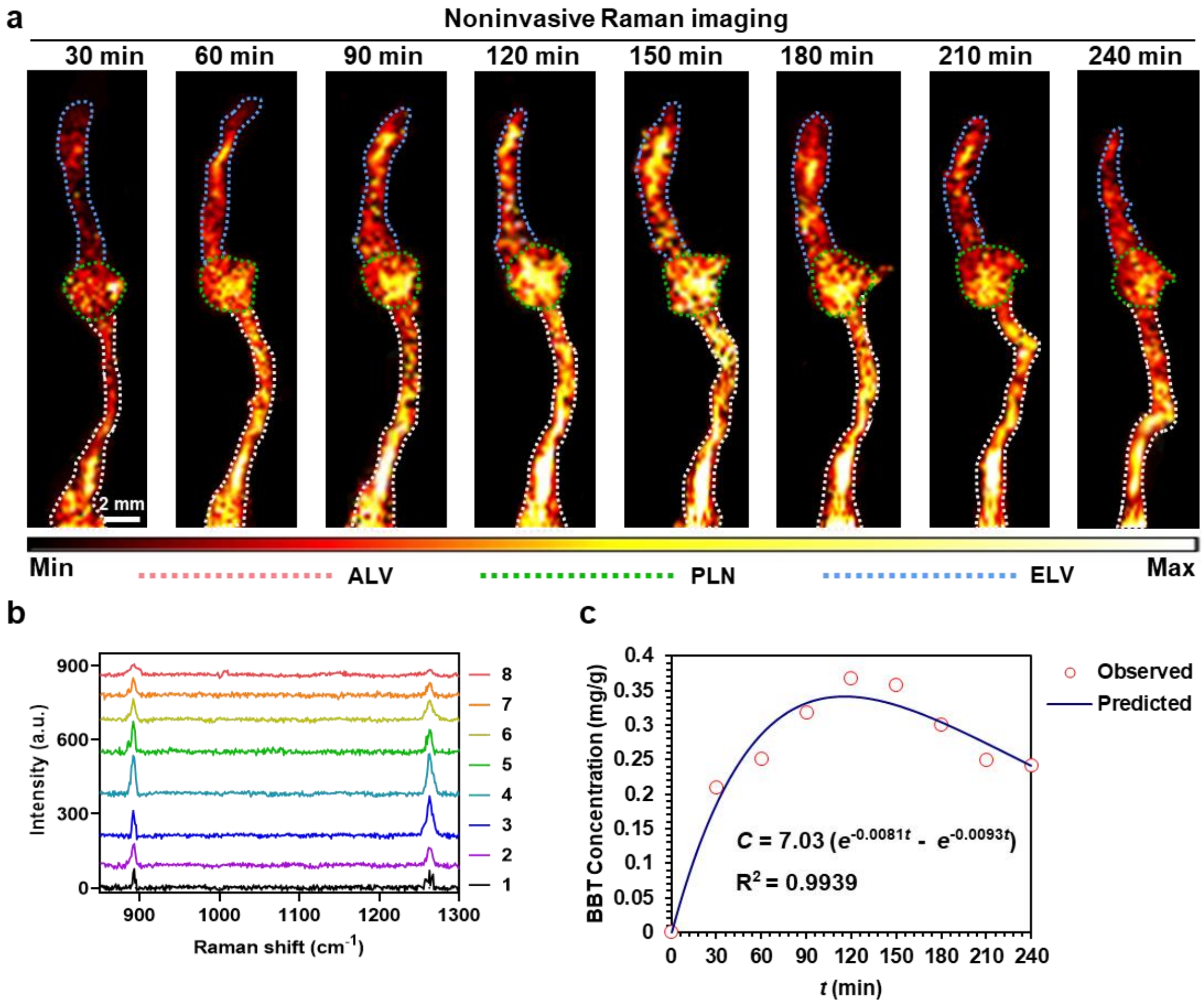

Figure S15. Continuously quantitative SICTERS imaging of Mouse 11 of micellar vaccines and the pharmacokinetics of micellar lymphatic transport via the PBPK model. (a) The non-invasive *in vivo* Raman imaging (894 cm^{-1}) was conducted at different time points after subcutaneous injection of 50 μL of micellar vaccines (3 mg/mL) into the mouse footpad. (b) Raman spectra of the numbered regions in the PLN in (a). (c) The concentration-time curves (predicted) of micellar vaccines in PLN by fitting the data of observed concentration at each time to Eq (**2**). Raman measurement was carried out with 785-nm laser excitation, a ×5 objective, a laser power of 62.6 mW, one time accumulation and an acquisition time of 0.6 s.

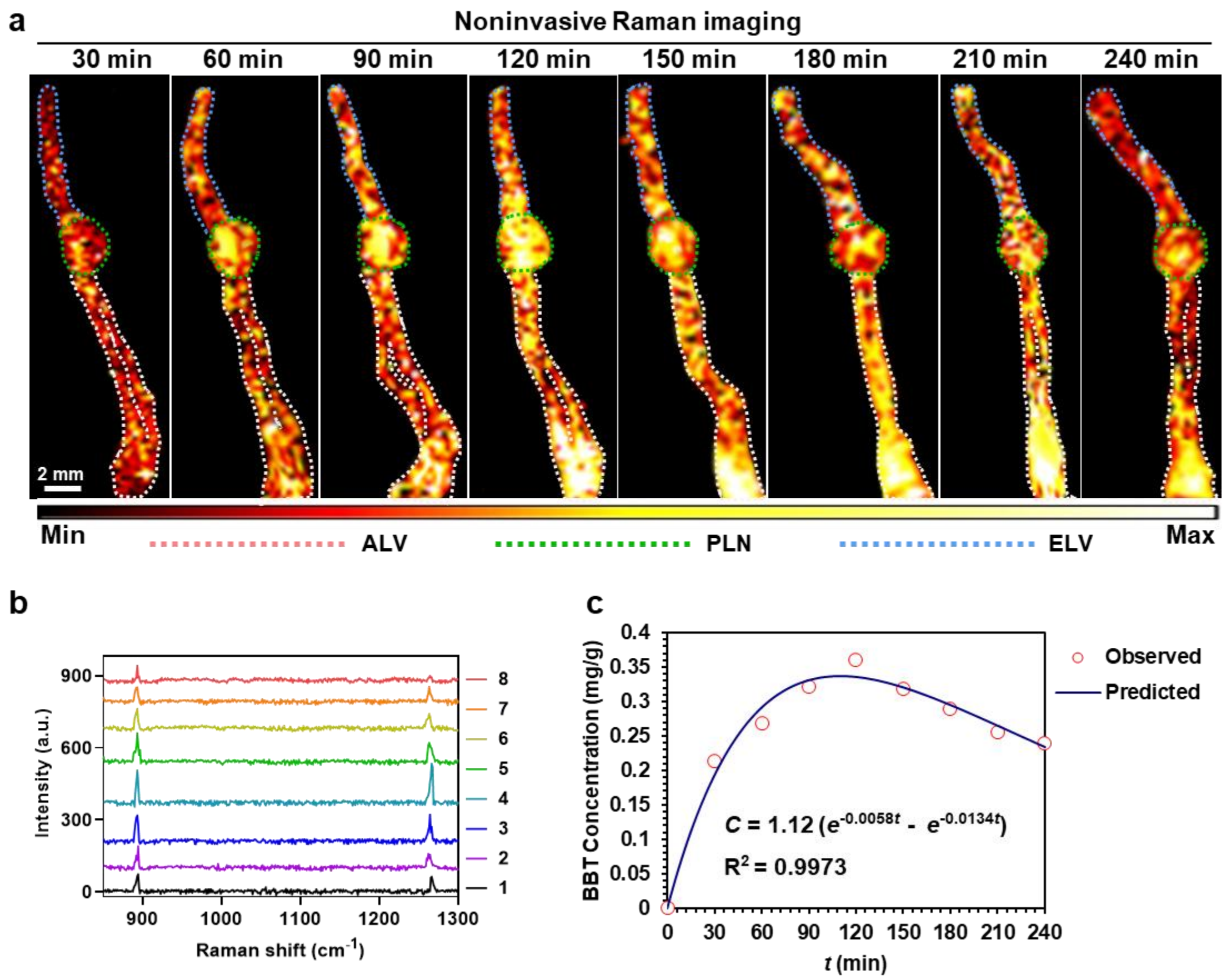

Figure S16. Continuously quantitative SICTERS imaging of Mouse 12 of micellar vaccines and the pharmacokinetics of micellar lymphatic transport via the PBPK model. (a) The non-invasive *in vivo* Raman imaging (894 cm^{-1}) was conducted at different time points after subcutaneous injection of 50 µL of micellar vaccines (3 mg/mL) into the mouse footpad. (b) Raman spectra of the numbered regions in the PLN in (a). (c) The concentration-time curves (predicted) of micellar vaccines in PLN by fitting the data of observed concentration at each time to Eq (**2**). Raman measurement was carried out with 785-nm laser excitation, a ×5 objective, a laser power of 62.6 mW, one time accumulation and an acquisition time of 0.6 s.

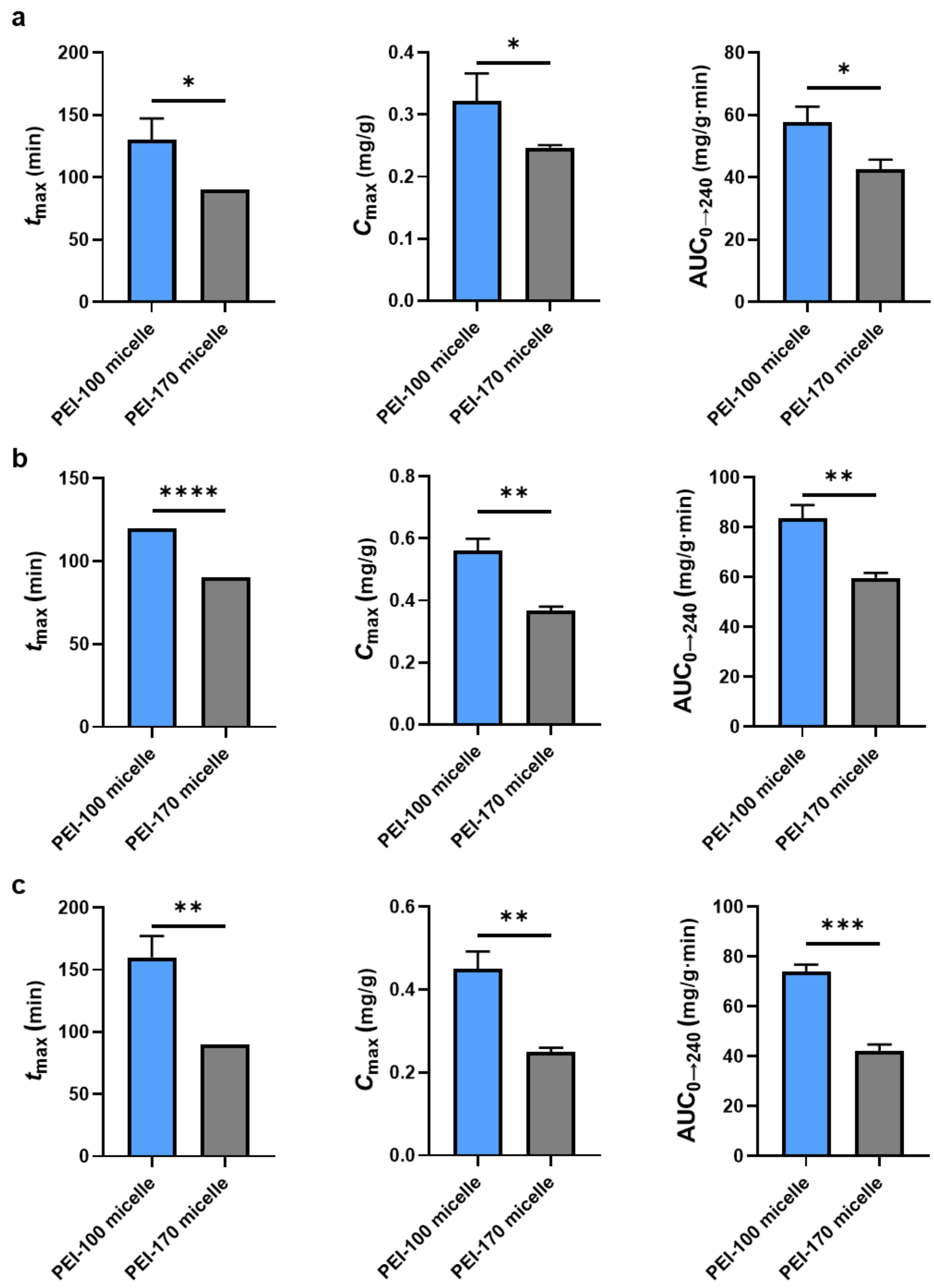

Figure S17. (a) The t_{max}, C_{max} and AUC values of PLN in Figure 3c of PEI-100 micelles and PEI-170 micelles using the non-compartmental model. (b) The t_{max}, C_{max} and AUC values of ALV in Figure 3d of PEI-100 micelles and PEI-170 micelles using the non-compartmental model. (c) The t_{max}, C_{max} and AUC values of ELV in Figure 3e of PEI-100 micelles and PEI-170 micelles using the non-compartmental model. Data are expressed as mean ± SD (n = 3). $^{*}P < 0.05$, $^{**}P < 0.01$, $^{***}P < 0.001$, $^{****}P < 0.0001$ analyzed by two-tailed Student's t-test.

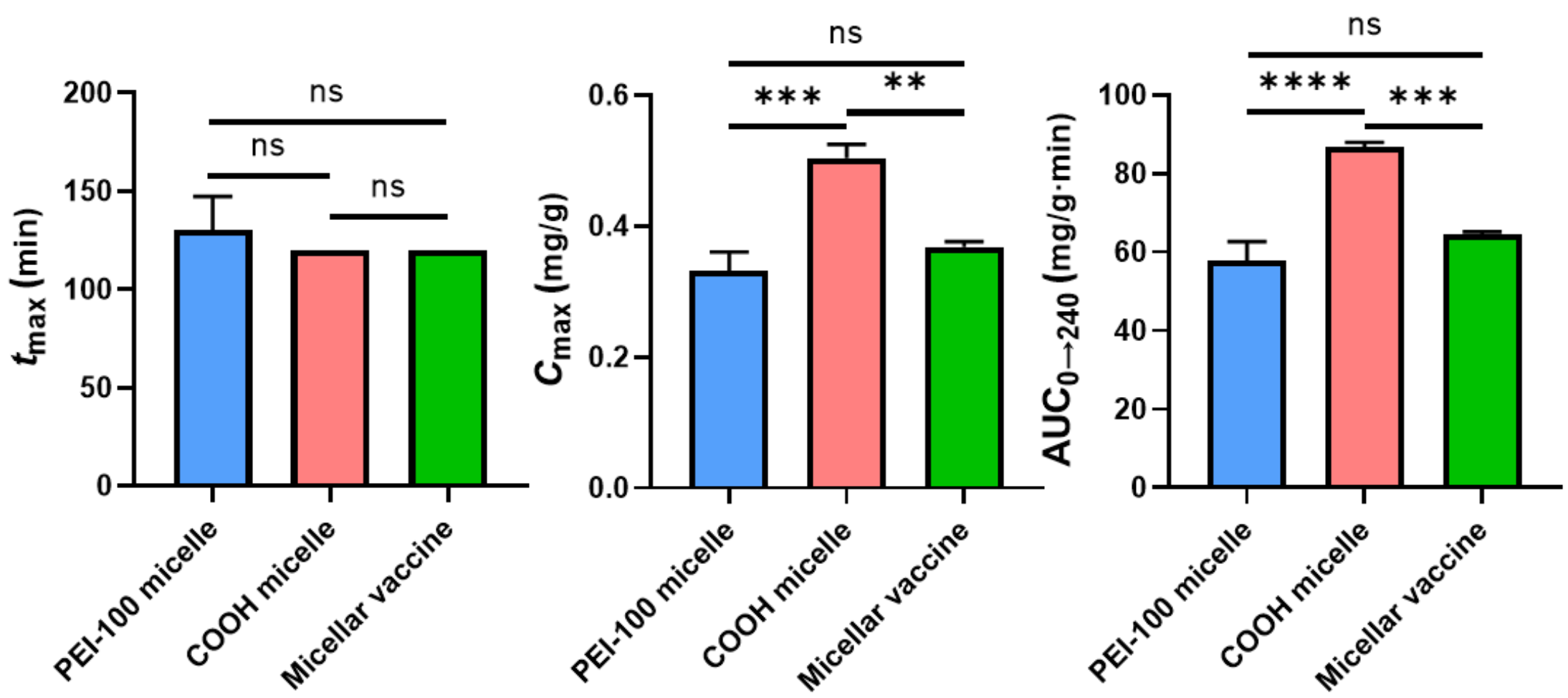

Figure S18. The t_{max}, C_{max} and AUC values of PLN in Figure 3c of PEI-100 micelles, COOH micelles and micellar vaccines using the non-compartmental model. Data are expressed as mean ± SD ($n = 3$). $^{**}P < 0.01$, $^{***}P < 0.001$, $^{****}P < 0.0001$, ns, no significant difference between the compared groups analyzed by one-way ANOVA with Tukey's post-hoc test.

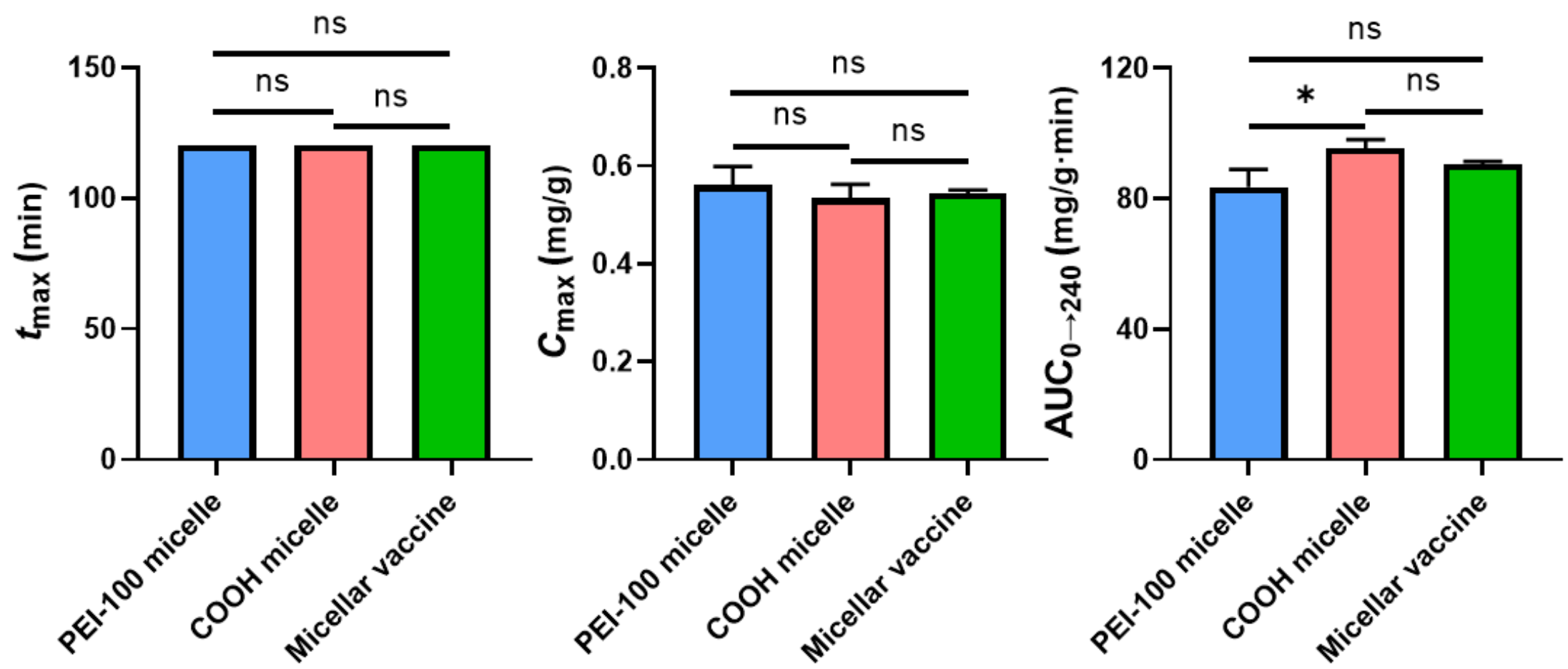

Figure S19. The t_{max}, C_{max} and AUC values of ALV in Figure 3d of PEI-100 micelles, COOH micelles and micellar vaccines using the non-compartmental model. Data are expressed as mean ± SD (n = 3). $^{*}P < 0.05$, ns, no significant difference between the compared groups analyzed by one-way ANOVA with Tukey's post-hoc test.

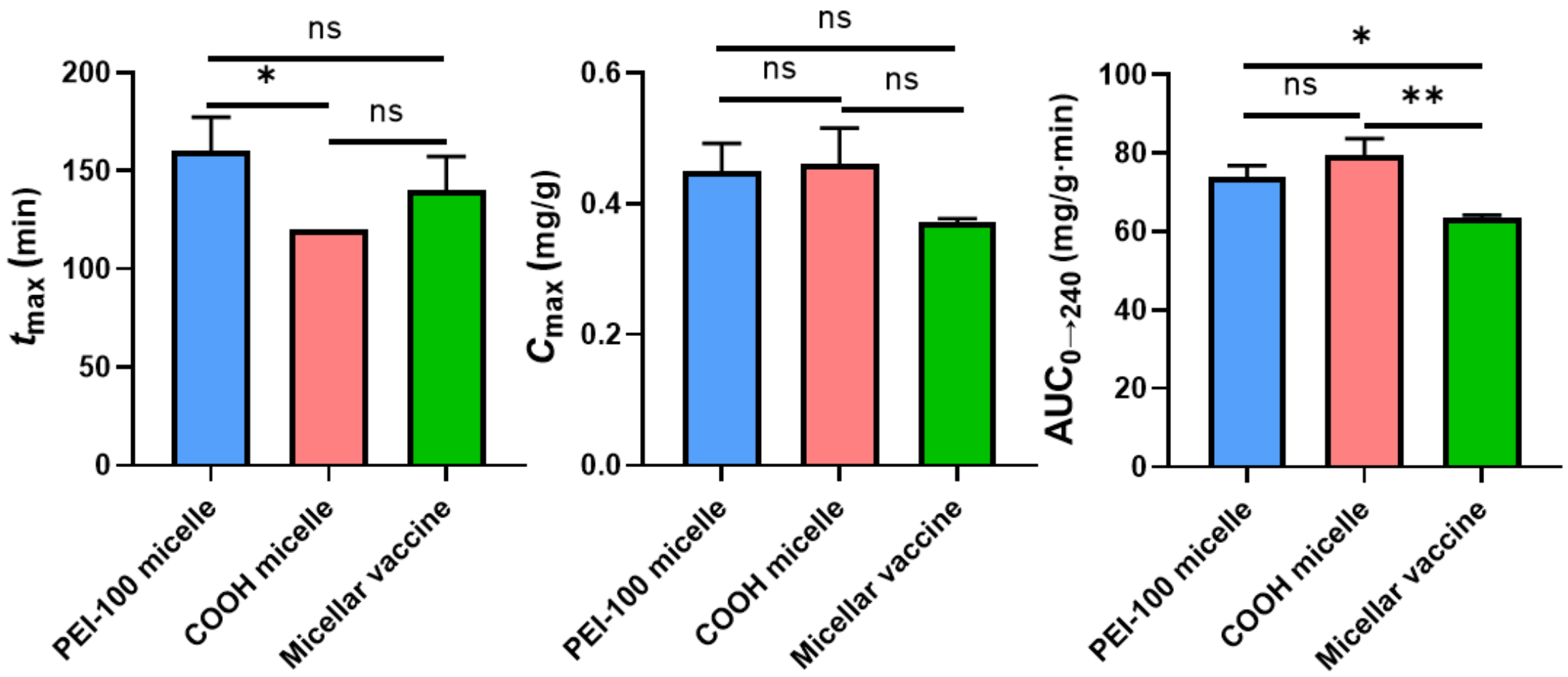

Figure S20. The t_{max}, C_{max} and AUC values of ELV in Figure 3e of PEI-100 micelles, COOH micelles and micellar vaccines using the non-compartmental model. Data are expressed as mean ± SD ($n = 3$). $^{*}P < 0.05$, $^{**}P < 0.01$, ns, no significant difference between the compared groups analyzed by one-way ANOVA with Tukey's post-hoc test.

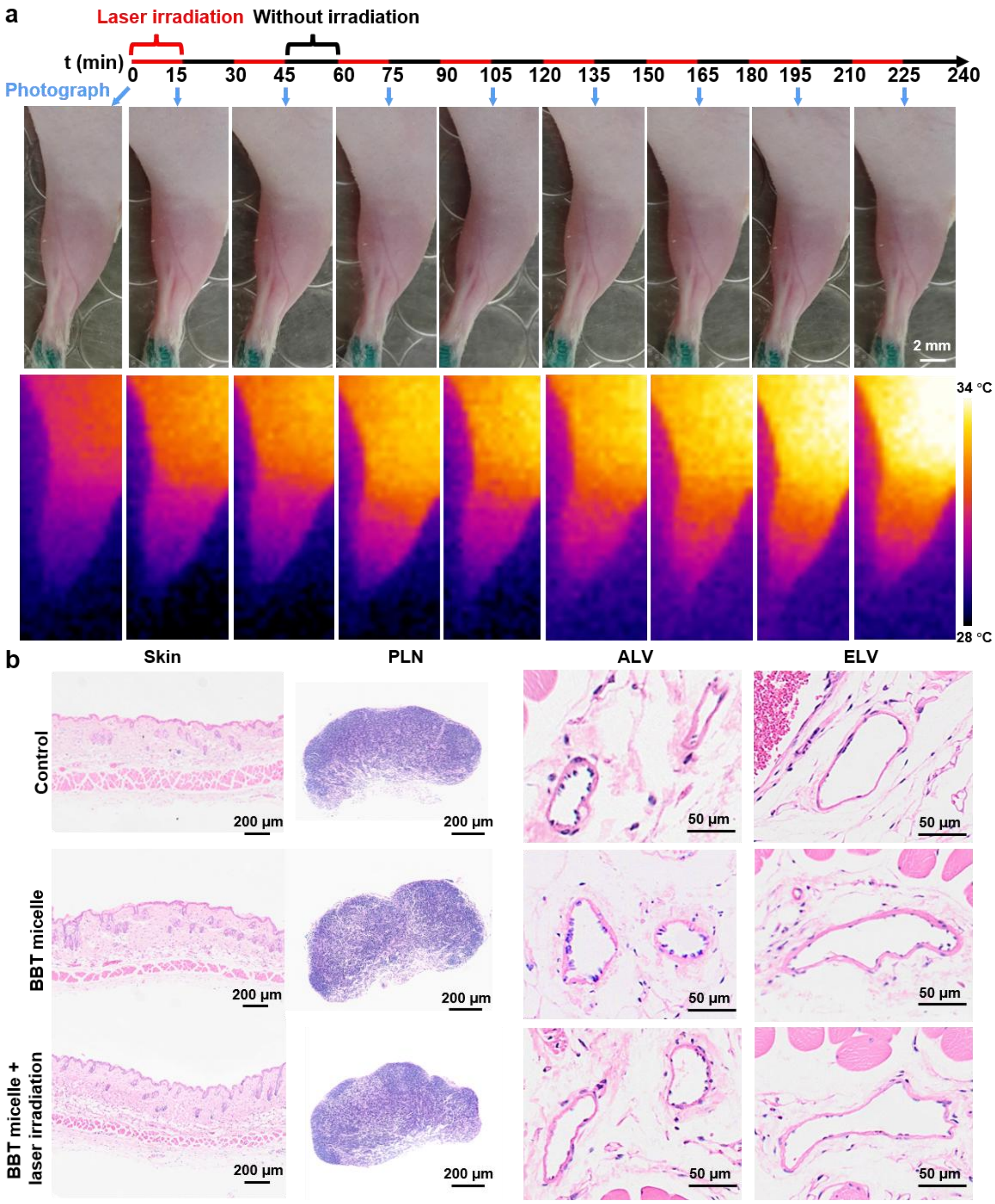

Figure S21. Biosafety evaluation of mice following repeated noninvasive Raman imaging. (a) Photographs (upper) and corresponding infrared thermal images (lower) of mouse legs with subcutaneous injection of PEI-100 micelles (150 μg BBT) during 8 cycles of noninvasive Raman imaging. Each cycle consisted of 15 min of Raman imaging with laser irradiation (red lines) followed by 15 min without imaging (black lines). Blue arrows, time points for photography and infrared thermal imaging. (b) Histologic analysis of skin, PLNs, ALVs and ELVs following repeated noninvasive Raman imaging at 240 min with H&E staining, respectively. Raman measurement was carried out with a 5 × objective, 785-nm laser excitation, a laser power of 62.6 mW, acquisition time of 0.6 s and one time accumulation.

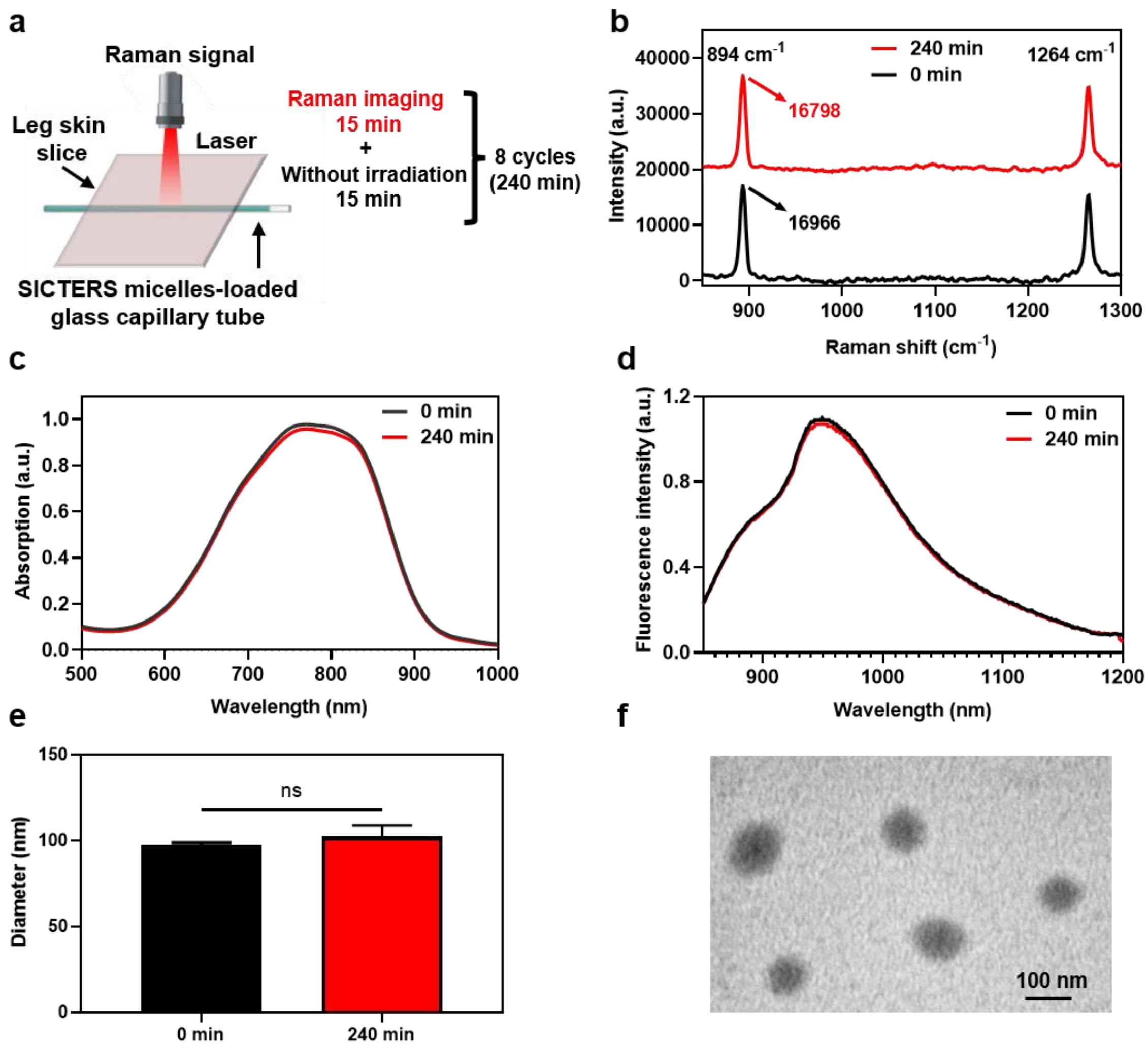

Figure S22. The photostability and micellar stability of SICTERS micelles after repeated Raman imaging over a 4 h period. (a) Raman imaging of capillary glass tube (inner diameter = 1 mm) containing PEI-100 micelles containing 0.4 mg/g of BBT covered with the leg skin of mice on the top. The sample received 8 cycles of Raman imaging. Each cycle consisted of Raman imaging with laser irradiation followed by another 15 min without irradiation. (b) Raman signal of the PEI-100 micelle solution at 0 min before the imaging and at 240 min after 8 cycles of repeated imaging in (a). Red Arrow, Raman peak at 894 cm^{-1}. (c) Absorption spectra of the PEI-100 micelle solution with 1:20 dilution (20 μg/g of BBT) at 0 min before Raman imaging and at 240 min after 8 cycles of Raman imaging in (a). (d) Fluorescence emission spectra of the PEI-100 micelle solution dissolved in tetrahydrofuran at 0 min before Raman imaging and at 240 min after 8 cycles of Raman imaging in (a). The fluorescence was excited at 808 nm following passing through an 850 nm long-pass filter. (e) Average particle size of the PEI-100 micelle solution at 0 min before Raman imaging and at 240 min after 8 cycles of Raman imaging, respectively. Mean ± SD (n = 3). ns, no significant difference analyzed by two-tailed Student's t-test. (f) Representative transmission electron micrograph of PEI-100 micelles at 240 min, after 8 cycles of Raman imaging. Scale bars, 100 nm. Raman measurement was carried out with a 5 × objective, 785-nm laser excitation, a laser power of 62.6 mW, acquisition time of 0.3 s and 3 times accumulation.

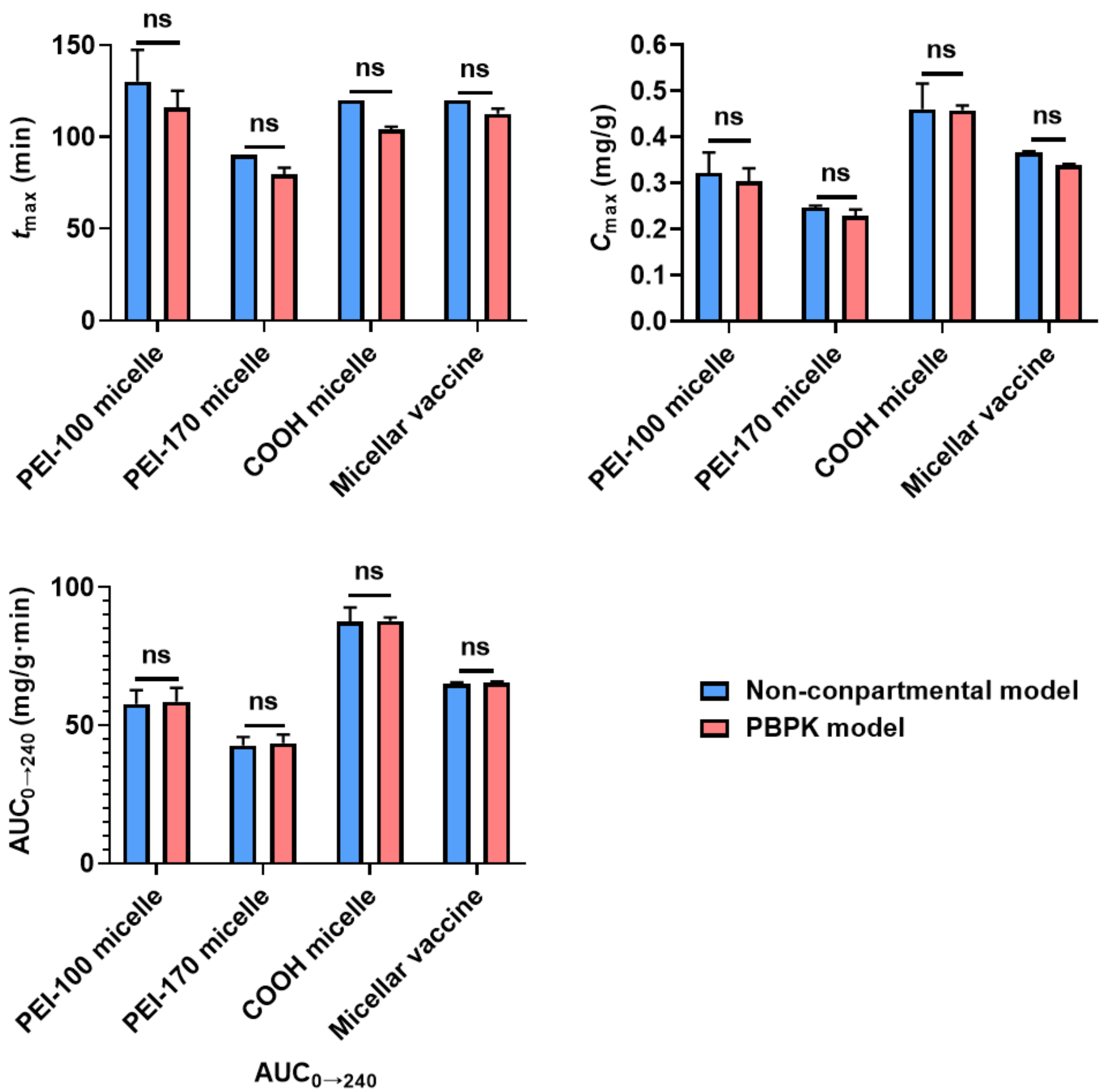

Figure S23. The t_{max}, C_{max} and AUC values of PLN calculated between using the non-compartmental model and by the PBPK model. Data are expressed as mean ± SD (n = 3). ns, no significant difference analyzed by two-way ANOVA with Tukey's post-hoc test.

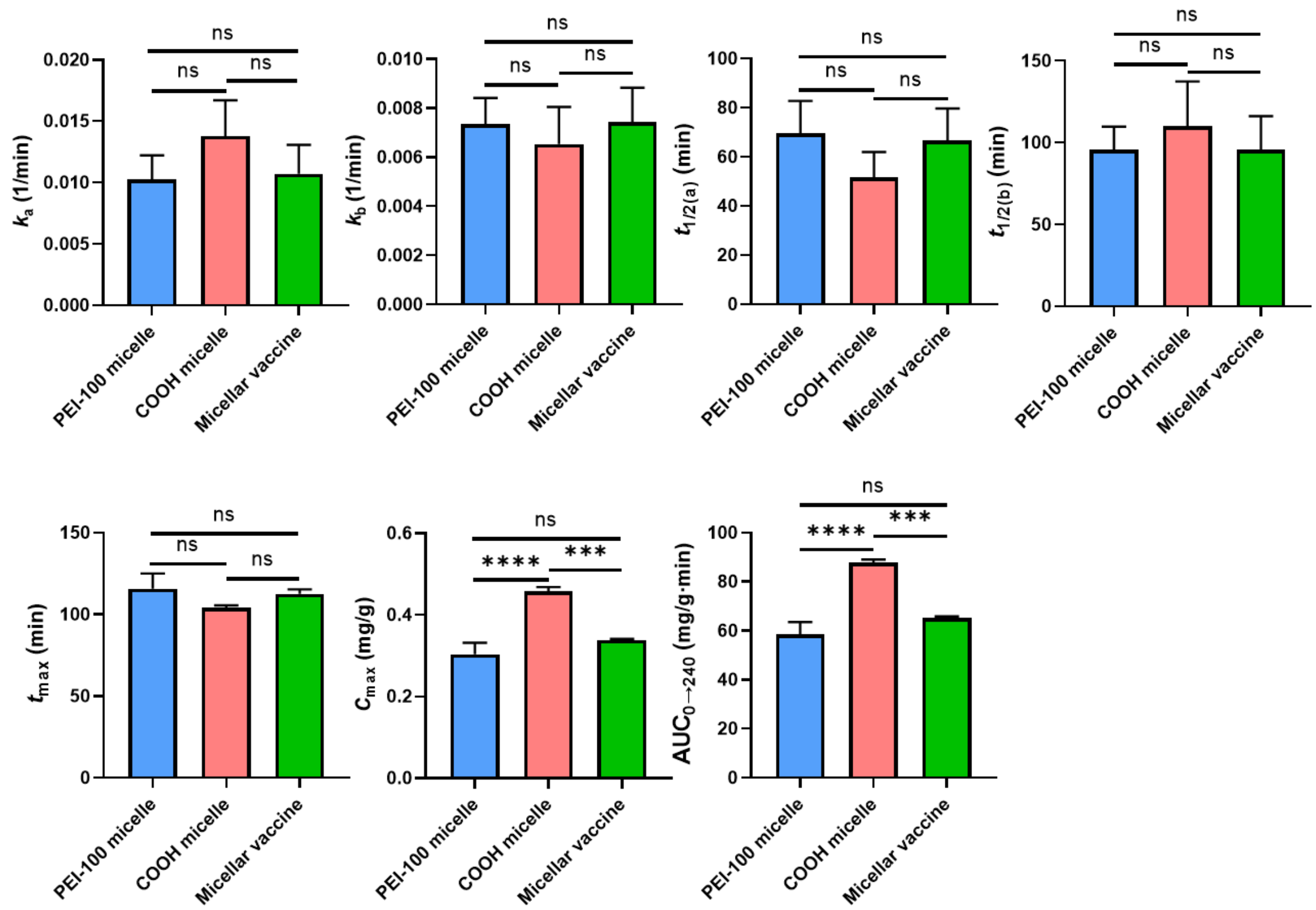

Figure S24. Pharmacokinetic parameters of PEI-100 micelles, COOH micelles or micellar vaccines obtained from Figure 4b, S4-S5, S9-S14. $t_{1/2(a)}$, influx half-life; $t_{1/2(b)}$, efflux half-life; t_{max}, time of the maximum concentration in PLN; C_{max}, maximum concentration in PLN; $AUC_{0\rightarrow240}$, AUC from 0 min to 240 min. Data are expressed as mean ± SD (n = 3). $^{***}P < 0.001$, $^{****}P < 0.0001$, ns, no significant difference between the compared groups analyzed by one-way ANOVA with Tukey's post-hoc test.

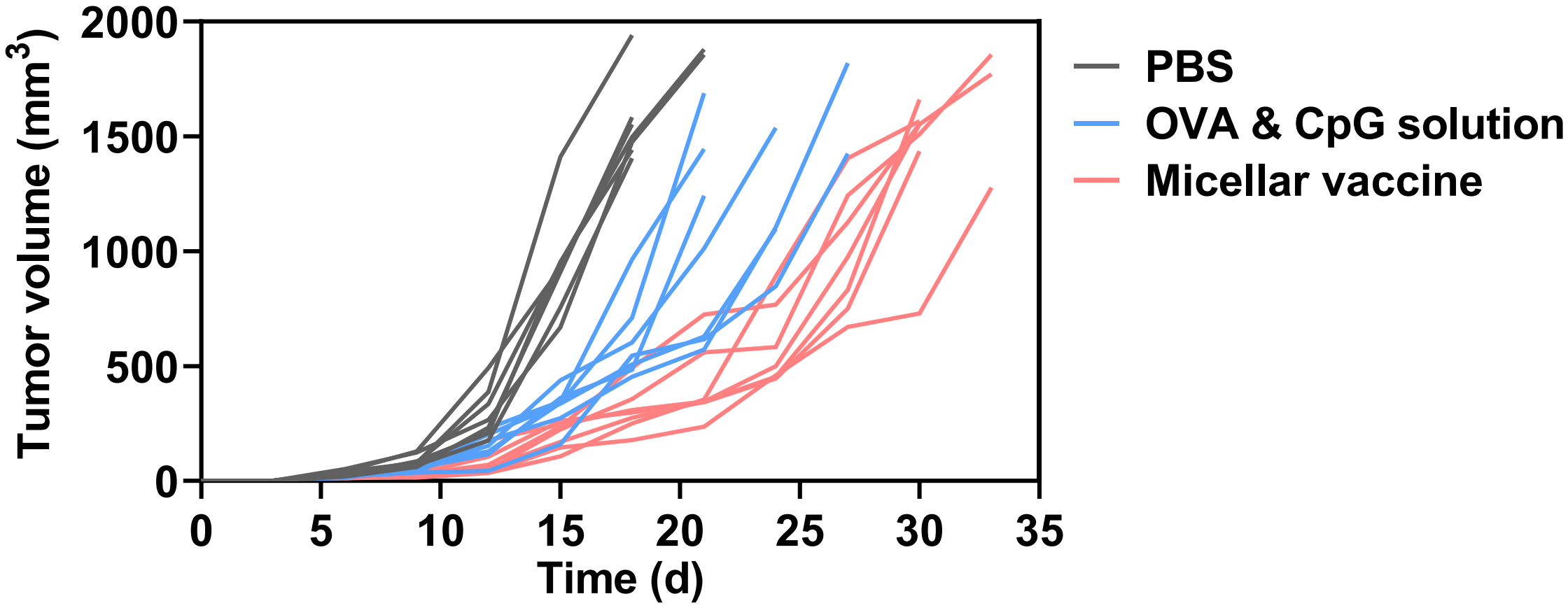

Figure S25. The individual tumor volumes of each group in Figure 5b (n = 7).

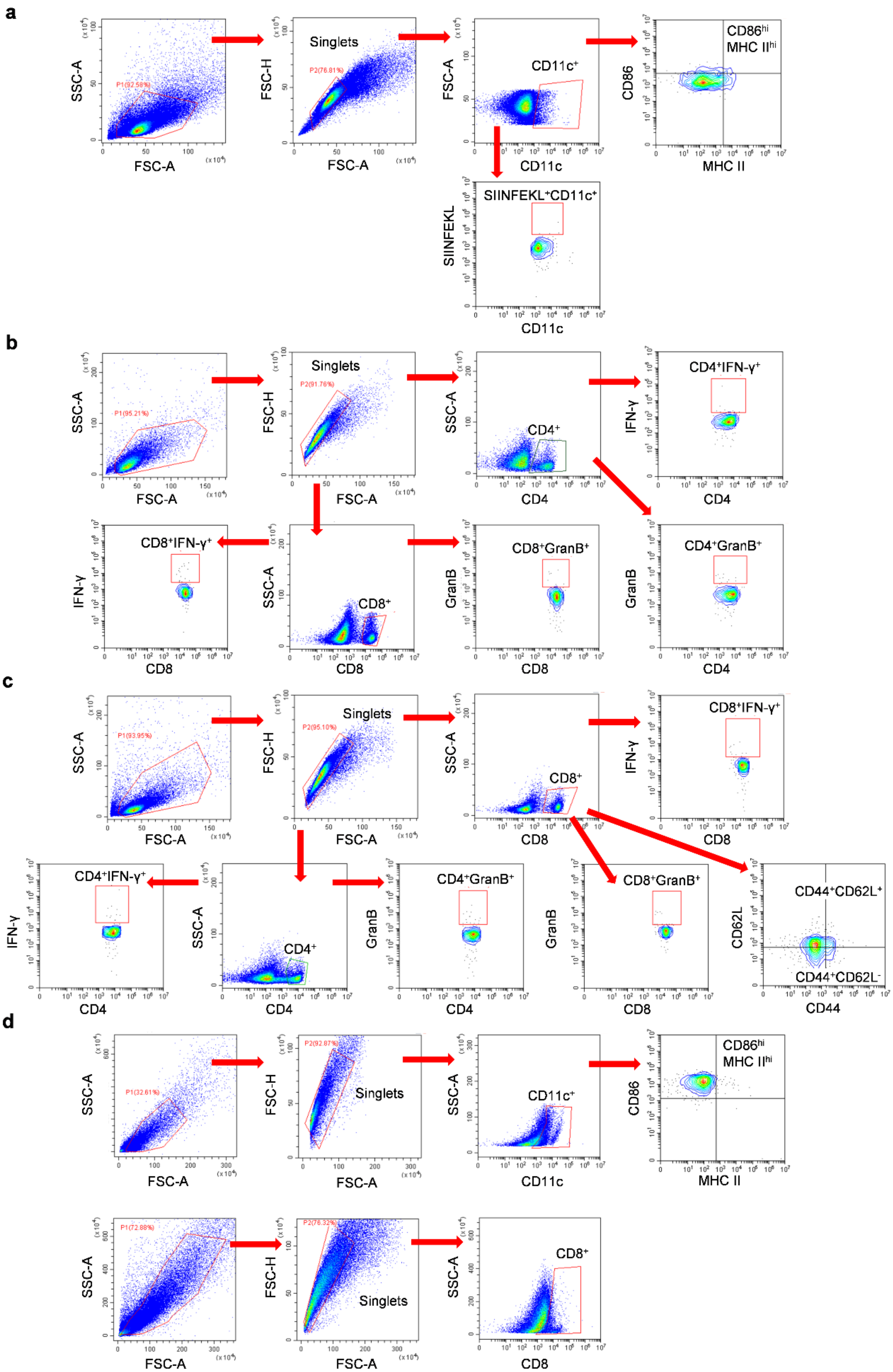
a
SSC-A
FSC-A
FSC-H
Singlets
FSC-A
CD11c⁺
CD11c
CD86
CD86hi
MHC IIhi
MHC II
SIINFEKL
SIINFEKL⁺CD11c⁺
CD11c
b
Singlets
CD4⁺
CD4⁺IFN-γ⁺
IFN-γ
CD4
CD8⁺IFN-γ⁺
CD8⁺
CD8
CD8⁺GranB⁺
GranB
CD4⁺GranB⁺
c
CD8⁺IFN-γ⁺
CD4⁺IFN-γ⁺
CD4⁺GranB⁺
CD8⁺GranB⁺
CD62L
CD44⁺CD62L⁺
CD44⁺CD62L⁻
CD44
d
CD11c⁺
CD86hi
MHC IIhi
CD8⁺
Singlets

Figure S26. Flow cytometry gating strategy. The general gating strategy started with FSC-A/SSC-A to exclude cell debris, followed by FSC-A/FSC-H to confirm singlets. (a) Gating strategy for analyzing matured ($CD11c^{+}CD86^{hi}MHC\ II^{hi}$) and antigen presentation ($SIINFEKL^{+}CD11c^{+}$) dendritic cells (DCs) in PLNs in Figure 5c and in Figure S27 a-b. (b) Gating strategy for analyzing $CD4^{+}$ T cells, $CD8^{+}$ T cells, IFN-$\gamma^{+}CD4^{+}$ T cells, IFN-$\gamma^{+}CD8^{+}$ T cells (CTLs), $GranB^{+}CD4^{+}$ T cells and $GranB^{+}CD8^{+}$ T cells in PLNs in Figure 5d-f and in Figure S27 c-g. (c) Gating strategy for analyzing $CD4^{+}$ T cells, $CD8^{+}$ T cells, IFN-$\gamma^{+}CD4^{+}$ T cells, CTLs, $GranB^{+}CD4^{+}$ T cells, $GranB^{+}CD8^{+}$ T cells, $CD8^{+}CD44^{+}CD62L^{+}$ T cells (central memory, CM) and $CD8^{+}CD44^{+}CD62L^{-}$ T cells (effector memory, EM) in spleen in Figure S28. (d) Gating strategy for analyzing matured ($CD11c^{+}CD86^{hi}MHC\ II^{hi}$) DCs and $CD8^{+}$ T cells in tumor in Figure S29.

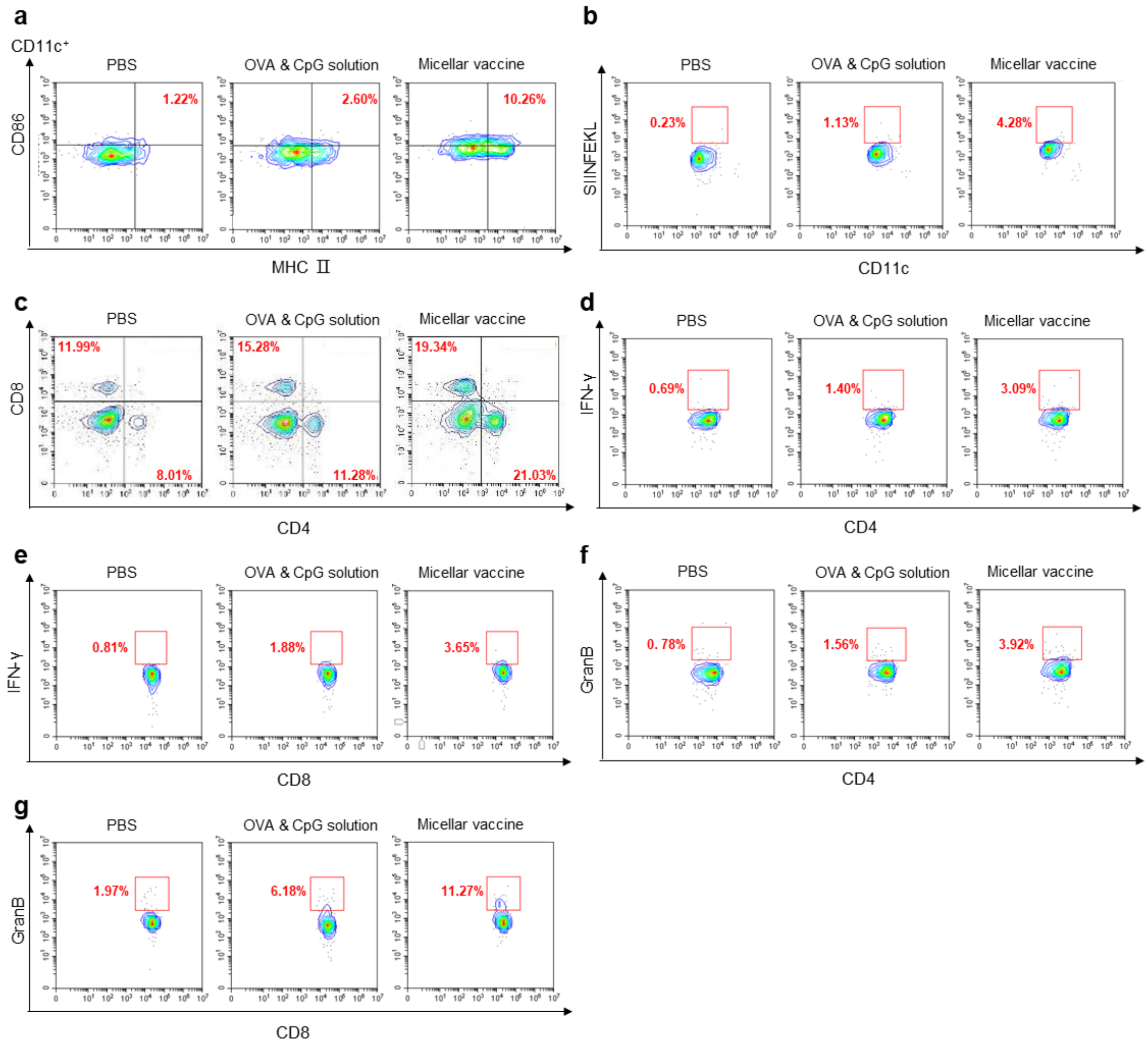

Figure S27. Representative flow cytometric plots of the results in Figure 5c-f. (a-b) The representative plots of matured ($CD11c^{+}CD86^{hi}MHC\ II^{hi}$) and antigen presentation ($SIINFEKL^{+}CD11c^{+}$) dendritic cells (DCs) in Figure 5c. (c) The representative plots of $CD4^{+}$ T cells and $CD8^{+}$ T cells in Figure 5d. (d-e) The representative plots of IFN-$\gamma^{+}$$CD4^{+}$ T cells and IFN-$\gamma^{+}$$CD8^{+}$ T cells (CTLs) in Figure 5e. (f-g) The representative plots of $GranB^{+}CD4^{+}$ T cells and $GranB^{+}CD8^{+}$ T cells in Figure 5f.

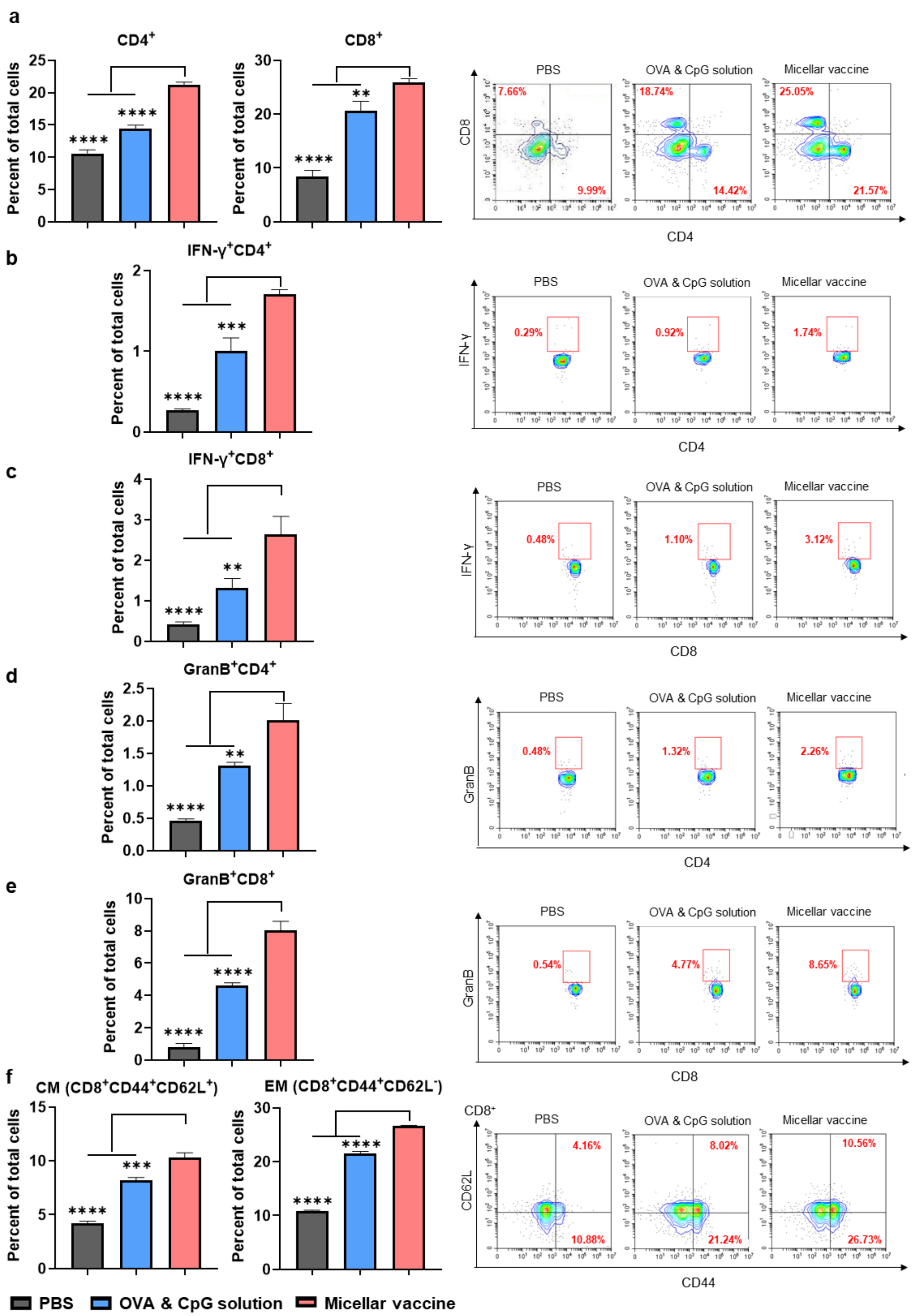

Figure S28. Flow cytometry analysis of different immune cells in spleens of B16-OVA-bearing mice at Day 21 after tumor inoculation. The mice were vaccinated with different

formulations according to the regimen in Figure 5a. (a) Flow cytometry analysis of $CD4^+$ T cells and $CD8^+$ T cells and their representative plots. (b) Flow cytometry analysis of IFN-γ^+CD4^+ T cells and their representative plots. (c) Flow cytometry analysis of IFN-γ^+CD8^+ T cells and their representative plots. (d) Flow cytometry analysis of $GranB^+CD4^+$ T cells and their representative plots. (e) Flow cytometry analysis of $GranB^+CD8^+$ T cells and their representative plots. (f) Flow cytometry analysis of $CD8^+CD44^+CD62L^+$ T cells (central memory, CM) and $CD8^+CD44^+CD62L^-$ T cells (effector memory, EM) and their representative plots. Data are expressed as mean ± SD ($n = 3$). $^{**}P < 0.01$, $^{***}P < 0.001$, $^{****}P < 0.0001$ between the compared groups analyzed by one-way ANOVA with Tukey's post-hoc test.

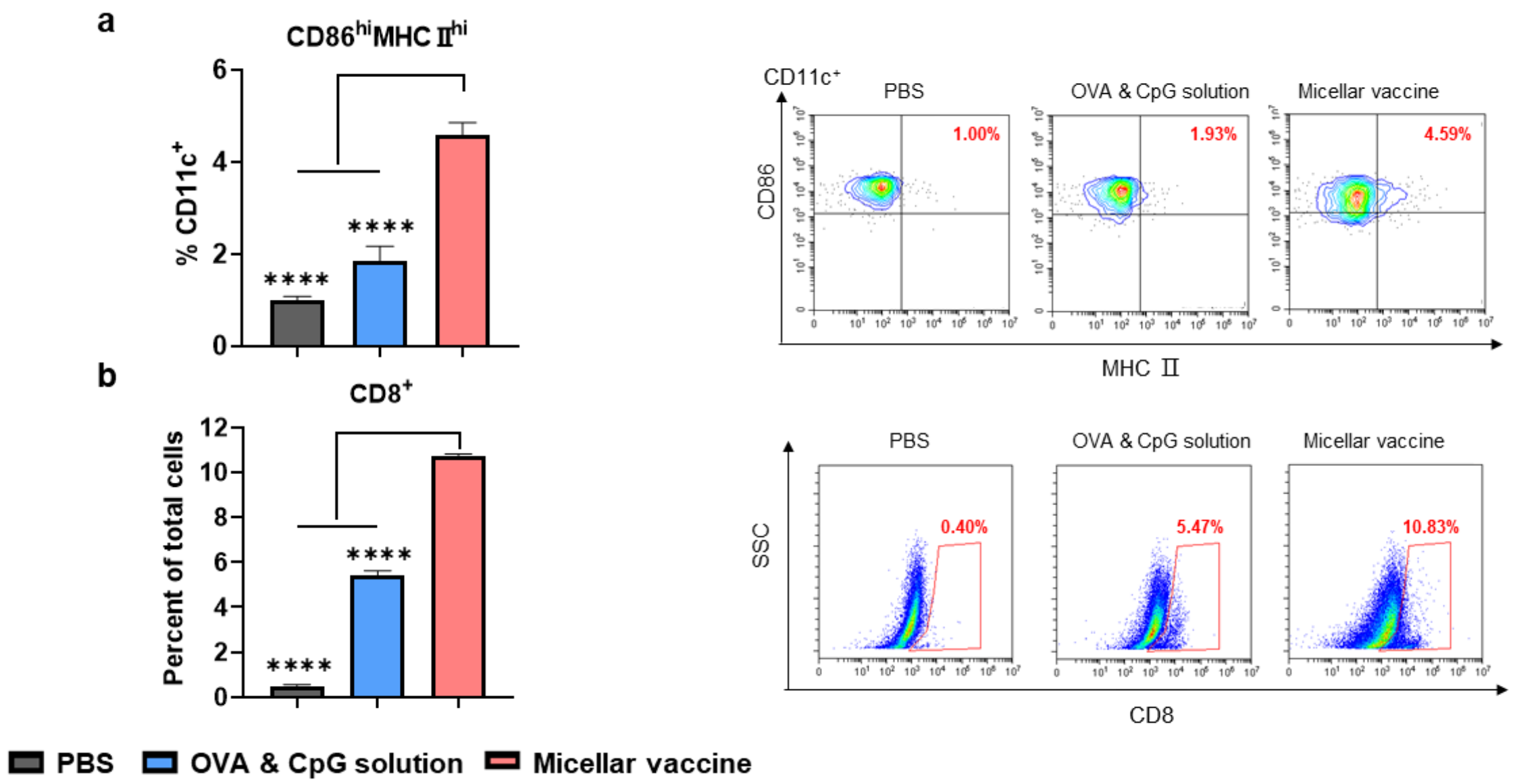

Figure S29. Flow cytometry analysis of different immune cells in the tumors of B16-OVA-bearing mice at Day 21 after tumor inoculation. (a) Flow cytometry analysis of matured ($CD11c^{+}CD86^{hi}MHC\ II^{hi}$) DCs and their representative plots. (b) Flow cytometry analysis of $CD8^{+}$ T cells and their representative plots. Data are expressed as mean ± SD (n = 3). $^{****}P < 0.0001$ between the compared groups analyzed by one-way ANOVA with Tukey's post-hoc test.

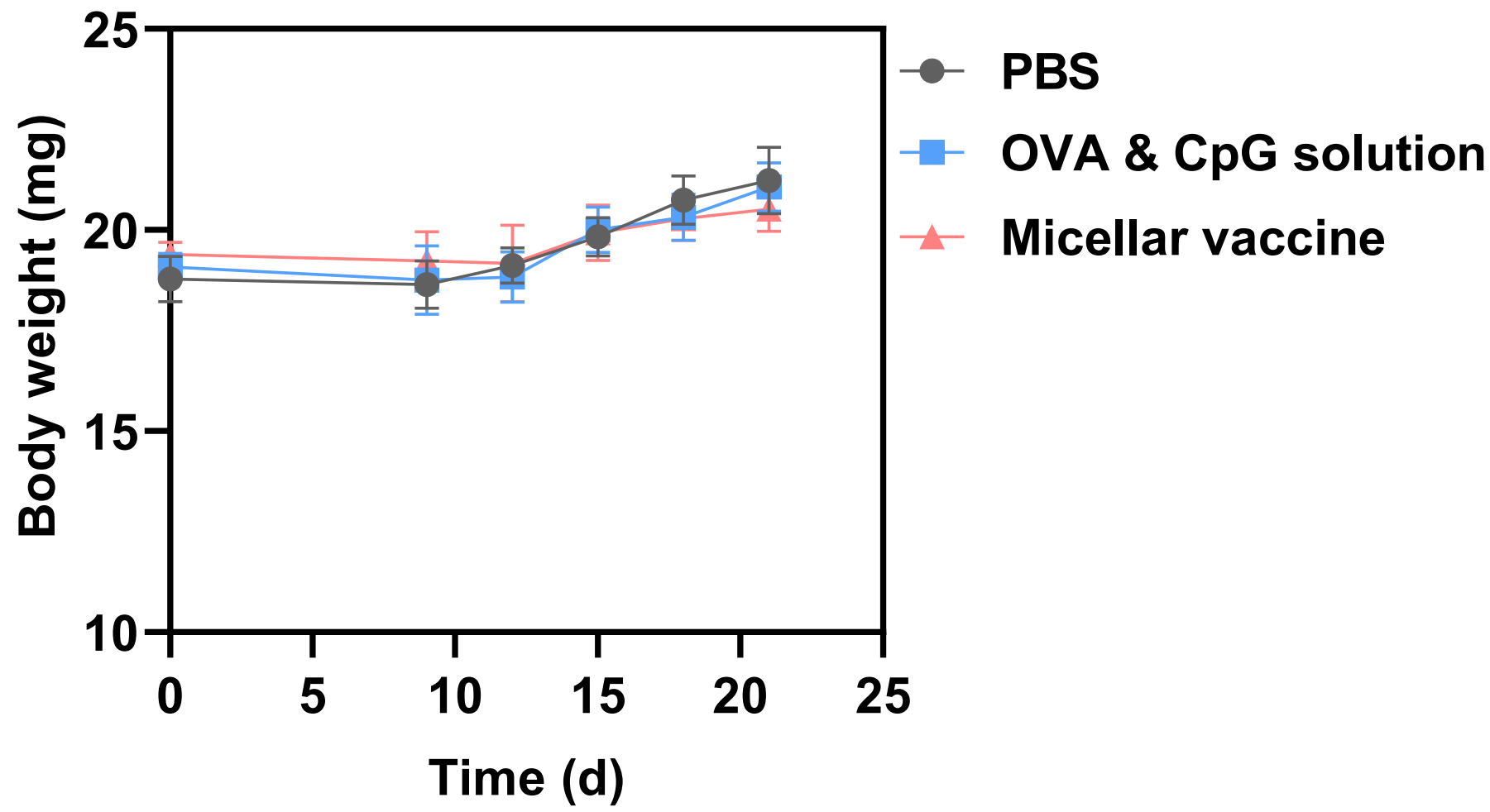

Figure S30. Body weight of B16-OVA-bearing melanoma mice immunized with different vaccines. Data are expressed as mean ± SD (*n* = 7).

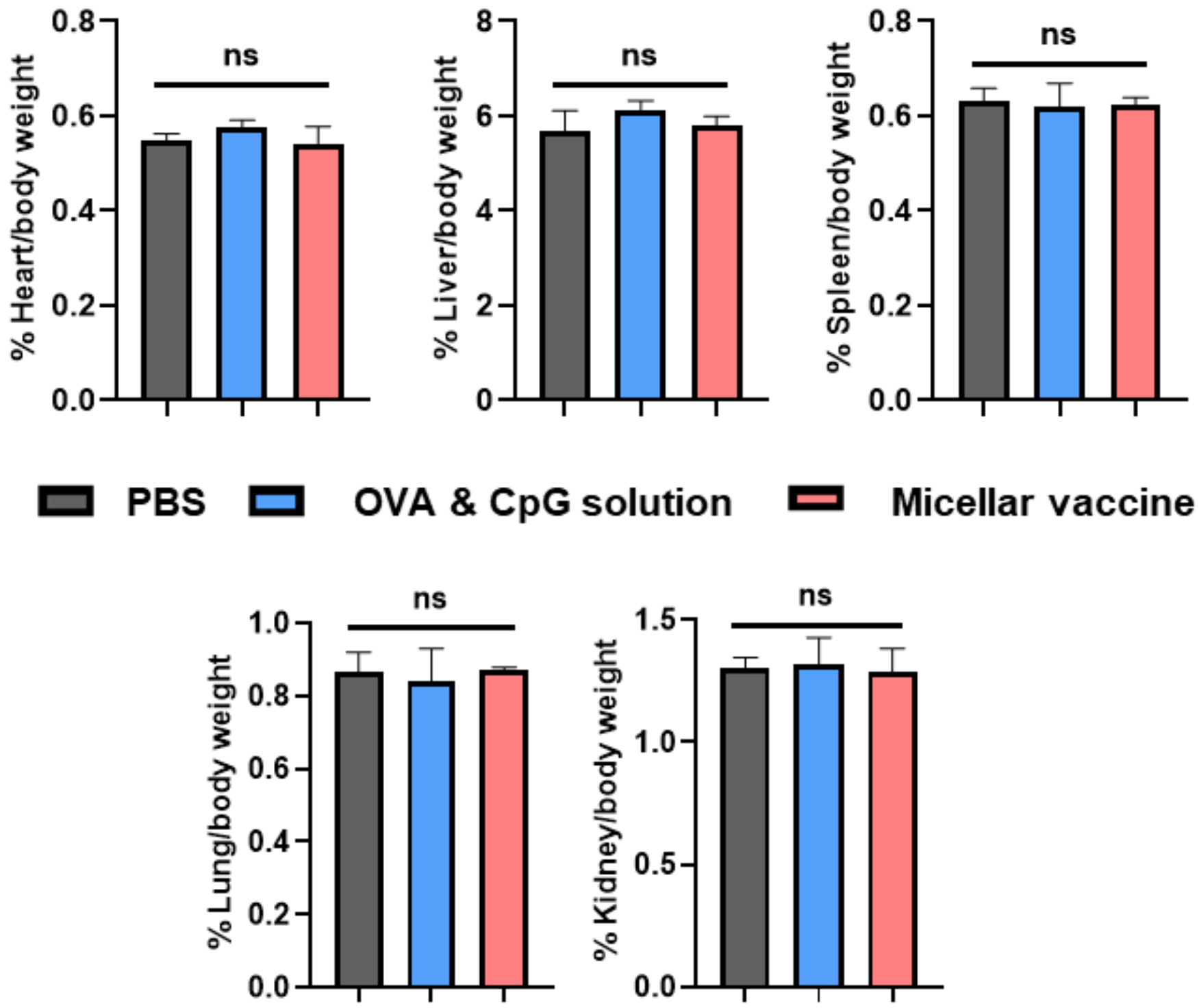

Figure S31. Organ coefficient of heart, liver, spleen, lung, kidney of healthy BALB/c mice following vaccination. PBS group, BALB/c mice were injected with PBS subcutaneously into footpad every seven days starting at Day 0 for 3 times. OVA & CpG solution group, mice were injected subcutaneously into footpad with solutions containing 100 μg of OVA and 25 μg of CpG every seven days starting at Day 0 for 3 times. Micellar vaccines group, mice were injected with micellar vaccines with 100 μg of OVA and 25 μg of CpG subcutaneously into footpad every seven days starting at Day 0 for 3 times. The organs were dissected at Day 15 post-primary immunization, for analysis. Data are expressed as mean ± SD (n = 3). ns, no significant difference analyzed by one-way ANOVA with Tukey's post-hoc test.

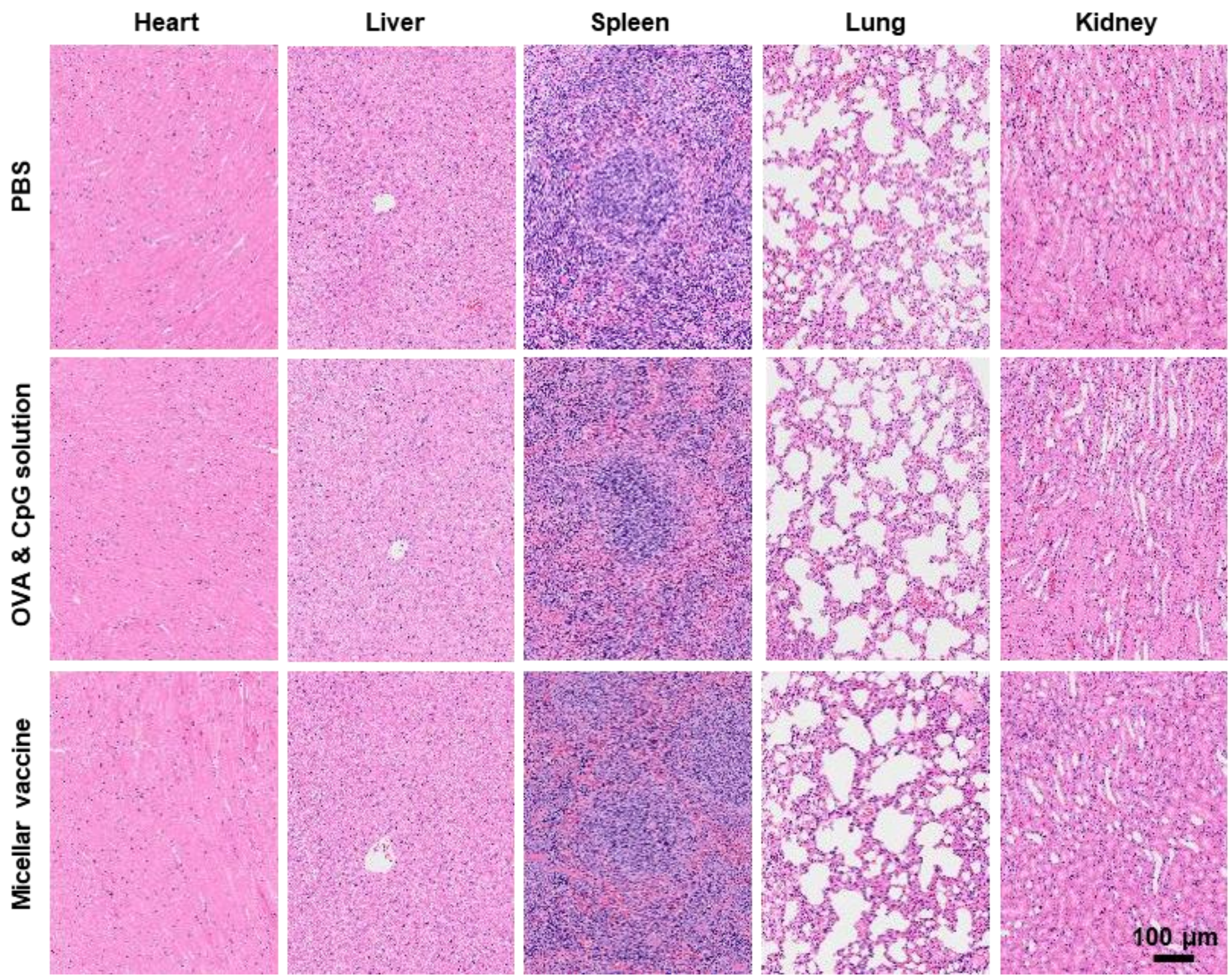

Figure S32. Histologic analysis of major organs of BALB/c mice following vaccination with different formulations. The organs were dissected at Day 15 post-primary immunization for sectioning and H&E staining.

Tables

Table S1. Vendors and catalog number of antibodies used

Antibody	Catalog number	Company
Anti-mouse CD86-FITC	11-0862-82	Invitrogen
Anti-mouse CD62L-PE	17-0621-81	Invitrogen
Anti-mouse CD11c-PerCP-Cyanine5.5	45-0114-82	Invitrogen
Anti-mouse CD44-PerCP-Cyanine5.5	45-0441-82	Invitrogen
Anti-mouse CD4-APC	17-0041-82	Invitrogen
Anti-mouse MHC II-APC	17-5321-82	Invitrogen
Anti-mouse IFN-γ- PerCP-Cyanine5.5	45-7311-80	Invitrogen
Anti-mouse granzyme B-FITC	396404	Biolegend
Anti-mouse CD8- PE	100708	Biolegend
Anti-mouse SIINFEKL-PE	141604	Biolegend

Table S2. Blood chemistry and hematologic analysis of mice[a]

Item	Reference range	PBS	Micellar vaccines
WBC (10^9/L)	0.8-10.6	1.5 ± 0.62	1.43 ± 0.33
LYMPH (10^9/L)	0.6-8.9	1.17 ± 0.06	0.9 ± 0.08
MONO (10^9/L)	0.04-1.4	0.1 ± 0.1	0.1 ± 0
NEUT (10^9/L)	0.23-3.6	0.27 ± 0.06	0.37 ± 0.09
LYMPH (%)	40-92	54.23 ± 4.72	51.93 ± 5.69
MONO (%)	0.9-18	5.2 ± 0.1	5.17 ± 0.12
NEUT (%)	6.5-50	39.8 ± 3.99	37.67 ± 4.92
RBC (1012/L)	6.5-11.5	7.36 ± 0.3	7.42 ± 0.38
HGB (g/dL)	110-165	113.67 ± 2.31	112 ± 5.1
HCT (%)	30-55	35.3 ± 0.44	34.5 ± 1.21
MCV (fL)	41-55	45.67 ± 0.64	46.03 ± 0.98
MCH (pg)	13-18	15.53 ± 0.25	15.5 ± 0.36
MCHC (g/dL)	300-360	330.67 ± 5.51	330.67 ± 3.68
RDW (%)	12-19	15.53 ± 0.06	15.33 ± 0.19
PLT (109/L)	400-1600	760 ± 16.52	746 ± 15.3
MPV (fL)	4.0-6.2	5.83 ± 0.31	5.83 ± 0.34
PDW	12.0-17.5	16.33 ± 0.31	16.43 ± 0.25
PCT (%)	0.100-0.780	0.46 ± 0.07	0.43 ± 0.02
ALT (U/L)	22.5-474.35	143.77 ± 11.1	154.17 ± 22.09
AST (U/L)	67-381	438.34 ± 21.1	449.27 ± 55.05
BUN (mg/dL)	7-31	22.2 ± 2	21.8 ± 0.97

[a] Hematologic analysis indicators include white blood cells (WBC), lymphocytes (LYMPH), monocytes (MONO), neutrophils (NEUT), the percentage of lymphocytes (LYMPH%), the percentage of monocytes (MONO%), the percentage of neutrophils (NEUT%), red blood cells (RBC), white blood cells (WBC). Hemoglobin (HGB), red blood cell backlog (HCT), mean red blood cell volume (MCV), mean red blood cell hemoglobin content (MCH), mean red blood cell hemoglobin concentration (MCHC), red blood cell distribution width (RDW), platelet number (PLT), mean platelet volume (MPV), platelet distribution width (PDW), platelet volume (PCT). Serum blood chemistry includes alkaline phosphatase (ALP), aspartate aminotransferase (AST) and blood urea (BUN). Hematological reference intervals for healthy female BALB/c mice were established based on standardized data from Charles River Laboratories: (http://www.criver.com/). Data are expressed as mean ± SD (n = 3).

Table S3. Representative SERS-based *in vivo* imaging

SERS probe	Penetration depth (mm)	Reference
Ag nanocubes	0.6-1.1 mm	[1]
SERS nanoparticles	0-1.5 mm	[2]
C-GERTs	< 1 mm	[3]
AuAg nanoshells	< 2 mm	[4]
Single-walled carbon nanotubes	< 2 mm	[5]
Pegylated SERS nanotags	< 2 mm	[6]
Au@Ag core-shell nanoparticles	< 2 mm	[7]
Au nanoshell SERS probe	1-2 mm	[8]
NIR-resonant SERS nanotags	1-2 mm	[9]

References:

1. C. H. Moran, M. Rycenga, X. Xia, et al., "Using Well-Defined Ag Nanocubes as Substrates to Quantify the Spatial Resolution and Penetration Depth of Surface-Enhanced Raman Scattering Imaging," *Nanotechnology* 25 (2014): 014007.
2. L. Lin, H. He, R. Xue, et al., "Direct and Quantitative Assessments of near-Infrared Light Attenuation and Spectroscopic Detection Depth in Biological tissues Using Surface-Enhanced Raman Scattering," *Med-X* 1 (2023): 9.
3. Y. Zhang, Z. Liu, B. D. Thackray, et al., "Intraoperative Raman-Guided Chemo-Photothermal Synergistic Therapy of Advanced Disseminated Ovarian Cancers," *Small* 14 (2018): 1801022.
4. L. Li, R. Jiang, B. Shan, et al., "Near-Infrared II Plasmonic Porous Cubic Nanoshells for in Vivo Noninvasive Sers Visualization of Sub-Millimeter Microtumors," *Nature Communications* 13 (2022): 5249.
5. S. Keren, C. Zavaleta, Z. Cheng, et al., "Noninvasive Molecular Imaging of Small Living Subjects Using Raman Spectroscopy," *Proceedings of the National Academy of Sciences* 105 (2008): 5844-9.
6. X. Qian, X.-H. Peng, D. O. Ansari, et al., "In Vivo Tumor Targeting and Spectroscopic Detection with Surface-Enhanced Raman Nanoparticle Tags," *Nature Biotechnology* 26 (2008): 83-90.
7. C. Qiu, W. Zhang, Y. Zhou, et al., "Highly Sensitive Surface-Enhanced Raman Scattering (Sers) Imaging for Phenotypic Diagnosis and Therapeutic Evaluation of Breast Cancer," *Chemical Engineering Journal* 459 (2023): 141502.
8. B. Deng, Y. Wang, X. Bu, et al., "Sentinel Lymph Node Identification Using near-Infrared II Ultrabright Raman Nanotags on Preclinical Models," *Biomaterials* 308 (2024): 122538.
9. J. H. Yu, I. Steinberg, R. M. Davis, et al., "Noninvasive and Highly Multiplexed Five-Color Tumor Imaging of Multicore near-Infrared Resonant Surface-Enhanced Raman Nanoparticles in Vivo," *ACS Nano* 15 (2021): 19956-69.
